# Impact of empiric potassium supplementation on mortality, sudden cardiac arrest and stroke in furosemide initiators

**DOI:** 10.1002/bcp.70584

**Published:** 2026-05-03

**Authors:** Thanh Phuong Pham Nguyen, Sean Hennessy, Colleen M. Brensinger, Warren B. Bilker, Laura M. Dember, Todd A. Miano, Tat‐Thang Vo, Allison W. Willis, Charles E. Leonard

**Affiliations:** ^1^ Department of Biostatistics, Epidemiology, and Informatics University of Pennsylvania Perelman School of Medicine Philadelphia Pennsylvania USA; ^2^ Department of Neurology Translational Center for Excellence for Neuroepidemiology and Neurological Outcomes Research University of Pennsylvania Perelman School of Medicine Philadelphia Pennsylvania USA; ^3^ Center for Real‐World Effectiveness and Safety of Therapeutics University of Pennsylvania Perelman School of Medicine Philadelphia Pennsylvania USA; ^4^ Center for Clinical Epidemiology and Biostatistics University of Pennsylvania Perelman School of Medicine Philadelphia Pennsylvania USA; ^5^ Leonard Davis Institute of Health Economics University of Pennsylvania Philadelphia Pennsylvania USA; ^6^ Renal‐Electrolyte and Hypertension Division, Department of Medicine University of Pennsylvania Perelman School of Medicine Philadelphia Pennsylvania USA; ^7^ Department of Statistics and Data Science, The Wharton School University of Pennsylvania Philadelphia Pennsylvania USA; ^8^ Department of Neurology University of Pennsylvania Perelman School of Medicine Philadelphia Pennsylvania USA

**Keywords:** empiric potassium supplementation, loop diuretic, mortality, pharmacoepidemiology, stroke, sudden cardiac arrest, ventricular arrhythmia

## Abstract

**Aim:**

A prior non‐randomized study suggests that potassium supplementation may improve survival among furosemide initiators, and a randomized trial suggests that salt substitutes containing potassium might lower stroke risk. We conducted a retrospective cohort study using health‐care data to confirm or refute these associations among new users of furosemide.

**Methods:**

The exposure of interest was empiric potassium dispensing (yes/no) concurrent with furosemide initiation. Outcomes were all‐cause mortality, sudden cardiac arrest/ventricular arrhythmia (SCA/VA), and stroke. Primary as‐started and secondary as‐treated analyses were performed with Cox proportional hazards regression. We used inverse probability of treatment weighting (IPTW) to control for confounding, with weights calculated from high‐dimensional propensity scores.

**Results:**

We identified 511 462 and 320 703 initiators of furosemide <40 and ≥40 mg/day with 21.5% and 35.3%, respectively, starting empiric potassium supplementation. In initiators of furosemide <40 mg/day with (*vs*. without) empiric potassium, as‐started IPTW‐hazard ratios (HRs) were 1.02 (95%CI 1.01–1.04) for death, 1.00 (0.94–1.04) for SCA/VA, and 1.03 (1.00–1.06) for stroke. Similarly, in initiators of furosemide ≥40 mg/day with (*vs*. without) empiric potassium, as‐started IPTW‐HRs were 1.02 (1.00–1.03) for death, 0.98 (0.94–1.03) for SCA/VA, and 1.01 (0.98–1.04) for stroke.

**Conclusion:**

We did not observe associations suggesting a clinically meaningful effect of empiric potassium supplementation among furosemide users. However, given the high prevalence of furosemide use among highly heterogeneous patient populations, a large pragmatic trial may be warranted to more definitively evaluate the potential benefits and harms of empiric potassium supplementation among furosemide initiators.

**What is already known about this subject**

- Previous observational studies and open‐label trials suggest potential benefits of empiric potassium supplementation or potassium‐containing salt substitutes on mortality or stroke.

**What this study adds**

- This study investigates whether empiric potassium supplementation affects mortality, arrhythmias, or stroke in new furosemide users.
- Observed associations were minimal and analytically sensitive, and given study limitations are unlikely to translate into clinically important effects.
- Because of furosemide's broad use across heterogenous populations, a large‐scale pragmatic trial might be needed to more definitively evaluate associated outcomes.

## 1 INTRODUCTION

Furosemide is a widely used loop diuretic to treat oedema or fluid overload in conditions such as heart failure (HF) and cirrhosis.^1^ Hypokalaemia is one of the most common adverse effects of furosemide,^1^, ^2^ occurring in 4–14% of individuals on loop diuretics.^3^ This electrolyte disturbance can lead to an increased risk of life‐threatening arrhythmias and mortality.^4^ In 2000, the National Council on Potassium in Clinical Practice recommended routine consideration of potassium supplementation for individuals with hypertension receiving a non‐potassium‐sparing diuretic and those with HF, even in the absence of hypokalaemia.^5^ A study using U.S. Medicaid data found that empiric potassium supplementation upon initiating a loop diuretic was associated with improved survival, with a greater apparent benefit seen among people on an initial furosemide dose of ≥40 mg/day.^6^ Furthermore, previous observational studies, randomized trials and meta‐analyses have shown that a greater potassium intake (through potassium salt substitution, potassium‐containing food, or potassium supplementation) was associated with a lower stroke frequency.^7^, ^8^ Nevertheless, more recent guidelines for hypertension, HF, and ascites do not recommend routine empiric potassium supplementation despite emphasizing the importance of adequate potassium consumption, preferably through dietary modification.^9^, ^10^, ^11^, ^12^, ^13^ As data from randomized trials are unavailable to examine the potential benefits and harms of empiric potassium supplementation, we used health‐care data to conduct a retrospective cohort study, focusing on the most frequently prescribed loop diuretic—furosemide. The objective of this study was to confirm or refute the real‐world effect of empiric potassium supplementation, initiated proactively at the same time with furosemide, in reducing long‐term (1) all‐cause mortality, (2) sudden cardiac arrest/ventricular arrhythmia (SCA/VA), and (3) stroke (both ischaemic and haemorrhagic, considered as a composite outcome) risk in two distinct cohorts of new furosemide users (<40 mg/day and ≥40 mg/day).

## 2 METHODS

### 2.1 Ethics statement

The University of Pennsylvania Office of Regulatory Affairs granted an Institutional Review Board exemption for this study. All methods were performed according to relevant guidelines and regulations.

### 2.2 Overview and data source

We performed a retrospective cohort study examining all‐cause mortality, SCA/VA and stroke risk among new users of furosemide, comparing those initiating *vs*. not initiating empiric potassium supplementation at the same time. We used inverse probability treatment weighting (IPTW) of high‐dimensional propensity score (hdPS) to mitigate confounding. We used 2007–2019 data from Optum's de‐identified Clinformatics® Data Mart Database (CDM). CDM contains health‐care claims data from >70 million commercially insured Americans (Supporting Information). The study design is illustrated in Figure S1.

### 2.3 Study population

#### 2.3.1 Identification of furosemide new users and definition of furosemide courses

We identified new users of furosemide from 1 January 2007 through 31 December 2019 using National Drug Codes (NDCs) from Lexicon Plus (Oracle Corporation, Austin, Texas). New furosemide users were defined by having no prescription fills for any loop diuretic in a 12‐month continuous baseline (hereafter baseline) period before initiating furosemide; the date on which new user criteria were met was the index date. We excluded people with a baseline potassium supplement dispensing or diagnosis of hypokalaemia, hyperkalaemia, or dialysis‐dependent chronic kidney disease (CKD)^14^ to minimize capturing potassium prescriptions potentially intended for therapeutic correction rather than empiric supplementation.

We categorized new users of furosemide into two cohorts based on initial daily furosemide dose—cohort 1: <40 mg/day and cohort 2: ≥40 mg/day, as 40 mg/day is the median initial daily dose recommended for the treatment of chronic HF congestion,^9^ the most common indication for furosemide.^15^ Furosemide courses began with the first qualifying furosemide dispensing and ended with the earliest among (1) an outcome; (2) death (for the SCA/VA and stroke analyses); (3) end of database (31 December 2019); or (4) disenrollment from the health plan, with a maximum enrolment gap of 30 days, for primary ‘as‐started’ analyses, which emulate intention‐to‐treat analyses in a trial with treatment assignment by an investigator.^16^, ^17^ Additional censoring criteria for secondary ‘as‐treated’ analyses included (5) switching to another diuretic and (6) furosemide discontinuation (i.e. no new furosemide dispensing after permitting a grace period corresponding to 20% of the days' supply of the latest furosemide prescription).

#### 2.3.2 Ascertainment of exposure to empiric potassium and baseline covariate selection and measurement

The exposure of interest was the dispensing of an oral potassium supplement on the *same* day as the initial furosemide prescription, which suggests a clinical strategy to supplement with potassium empirically. The exposure definition did not consider later oral potassium supplement dispensing, as these occurrences may be reactive to laboratory values or signs/symptoms of hypokalaemia rather than reflective of empiric decision‐making. The reference exposure was the absence of oral potassium supplement dispensing on the same day as the initial furosemide prescription.

We used the data‐adaptive hdPS method^18^, ^19^, ^20^ to identify potential confounders (Tables S1 and S2). The PS—the predicted probability of receiving (*vs*. not receiving) empiric potassium conditional on baseline covariates—was calculated for individuals in each furosemide cohort using logistic regression models. We then weighted subjects by the inverse probability of treatment received using the PS. IPTW created a synthetic sample where treatment assignment was independent of measured baseline covariates and allowed for unbiased estimation of average treatment effects under the assumptions of exchangeability, positivity, consistency, and no misspecification of the PS model.^21^

#### 2.3.3 Ascertainment of outcomes

The primary outcome was all‐cause mortality. Our dataset contained only the month and year of death, so we assumed death occurred on the month's last day.^22^, ^23^ Secondary outcomes were (1) SCA/VA and (2) stroke (i.e. haemorrhagic, ischaemic, and transient ischaemic attacks), resulting in an ED visit or hospital admission after furosemide initiation (Table S3).

### 2.4 Statistical analyses

We compared baseline characteristics of furosemide initiators (stratified by dose) with *vs*. without empiric potassium supplementation before and after PS weighting. We prespecified an absolute standardized difference >0.10 as indicating a meaningful imbalance.^24^

#### 2.4.1 Primary and sensitivity analyses

We conducted primary as‐started analyses^16^, ^17^ using IPTW with weights derived from the PS to estimate the effect of empiric potassium supplementation on the hazards of death, SCA/VA or stroke. We conducted a prespecified sensitivity analysis in which we trimmed extremes from the PS distribution (Supporting Information Methods).^25^ As‐started IPTW‐adjusted Kaplan–Meier curves^26^, ^27^ were used to illustrate survival probability, SCA/VA free‐probability, and stroke‐free probability over time in persons with and without empiric potassium within each furosemide dose cohort. This approach allows for a graphical depiction of Kaplan–Meier‐type survival curves that are standardized to the entire population under treatment with empiric potassium *vs*. without empiric potassium while accounting for confounding by multiple covariates.^27^

Cox proportional hazards regression models, weighted by IPTWs, were used with a robust variance estimator.^28^, ^29^ Each outcome was regressed on an indicator variable denoting receipt of empiric potassium. We assessed the proportional hazards assumption by visual examination of IPTW‐adjusted Kaplan–Meier curves.^26^ Similar IPTW‐adjusted Cox proportional hazards models were performed for secondary as‐treated analyses.

#### 2.4.2 Subgroup and instrumental variable (IV) analyses

See details in Supporting Information Methods.

### 2.5 Nomenclature of targets and ligands

Key protein targets and ligands in this article are hyperlinked to corresponding entries in http://www.guidetopharmacology.org, and are permanently archived in the Concise Guide to PHARMACOLOGY 2021/22.^30^

## 3 RESULTS

### 3.1 Cohort composition and baseline characteristics

Among 832 165 persons meeting inclusion criteria and initiating therapy with furosemide during the study period, 511 462 (61.5%) initiated a dose of <40 mg/day, and 320 703 (38.5%) initiated ≥40 mg/day (Table 1). The mean follow‐up duration for was 928.2 ± 894.9 days, with a median of 634 days (interquartile range: 244–1339). In the furosemide <40 mg/day cohort, the proportion of follow‐up days covered by an active potassium dispensing was 34.0% (as‐started) and 83.8% (as‐treated) among empiric potassium users, compared with 5.1% (as‐started) and 3.6% (as‐treated) among reference ‘non‐users’. Similarly, in the furosemide ≥40 mg/day cohort, potassium coverage was 34.6% (as‐started) and 83.7% (as‐treated) among empiric potassium users, *vs*. 6.8% (as‐started) and 5.2% (as‐treated) among ‘non‐users’. Before IPTW, cohorts included persons similarly aged—with medians ranging from 72.3 to 72.9 years (<40 mg/day) and 70.6 to 70.9 years (≥40 mg/day). The <40 mg/day cohort included a greater proportion of females (59.9–60.8% *vs*. 48.5–50.2%). Before IPTW, a greater proportion of people starting on empiric potassium (*vs*. not) were residing in long‐term care or hospitalized on index date in both furosemide cohorts (15.3% *vs*. 9.5% in the furosemide <40 mg/day cohort, and 27.1% *vs*. 17.3% in the furosemide ≥40 mg/day cohort). We also observed a similar proportion experiencing hospitalization within 30 days before the index date in both cohorts (24.6% *vs*. 17.5% in the furosemide <40 mg/day cohort, and 38.7% *vs*. 27.4% in the furosemide ≥40 mg/day cohort). Among initiators of furosemide ≥40 mg/day, a smaller proportion of people who started empiric potassium (*vs*. not) had CKD (21.8% *vs*. 28.2%), cirrhosis (1.1% *vs*. 2.9%) or ascites (1.8% *vs*. 3.6%), and had a history of aldosterone antagonist use (2.1% *vs*. 4.6%) before IPTW.

**TABLE 1:** Baseline characteristics among individuals initiating furosemide <40 mg/day or ≥40 mg/day with and without empiric potassium.

Characteristics	Furosemide <40 mg/day						Furosemide ≥40 mg/day					
	Before IPTW			After IPTW			Before IPTW			After IPTW		
	K^+^ = NO (*n* = 401 540)	K^+^ = YES (*n* = 109 922)	SDiff^a^	K^+^ = NO (*n* = 511 899^b^)	K^+^ = YES (*n* = 508 872^b^)	Weighted SDiff^a^	K^+^ = NO (*n* = 207 459)	K^+^ = YES (*n* = 113 244)	SDiff^a^	K^+^ = NO (*n* = 321 377^b^)	K^+^ = YES (*n* = 320 127^b^)	Weighted SDiff^a^
Propensity score, mean (SD)	0.20 (0.10)	0.27 (0.13)	**0.64**	0.22 (0.13)	0.22 (0.23)	0.08	0.31 (0.14)	0.43 (0.19)	**0.74**	0.35 (0.21)	0.35 (0.28)	0.00
Demographic												
Age in years, median (IQR)	72.3 (60.8–80.9)	72.9 (61.6–81.1)	0.04	72.4 (60.9–81.0)	72.4 (60.8–81.0)	−0.00	70.6 (59.7–79.2)	70.9 (60.4–79.2)	0.03	70.7 (60.0–79.2)	70.8 (59.9–79.3)	0.00
Age groups, % col			**0.11**			0.07			0.10			0.00
<35 years	2.6%	1.7%		2.4%	2.5%		2.5%	1.5%		2.2%	2.3%	
35–44 years	4.9%	4.5%		4.8%	4.9%		4.7%	4.2%		4.5%	4.5%	
45–54 years	9.6%	9.4%		9.5%	9.5%		10.4%	10.2%		10.3%	10.2%	
55–64 years	14.8%	15.0%		14.9%	14.9%		17.7%	18.0%		17.8%	17.6%	
65–74 years	25.7%	25.3%		25.6%	25.5%		27.7%	28.1%		27.8%	27.8%	
75–84 years	29.2%	31.0%		29.6%	29.7%		26.9%	28.5%		27.6%	27.6%	
85 + years	13.2%	13.1%		13.2%	13.0%		10.1%	9.5%		9.9%	10.0%	
Female sex, %col	60.8%	59.9%	−0.02	60.6%	60.7%	0.00	50.2%	48.5%	−0.03	49.6%	50.0%	0.01
Race, %col			**0.11**			0.00			0.10			0.00
White	66.8%	69.8%		67.4%	67.2%		65.0%	69.7%		66.7%	66.7%	
Black	11.6%	9.6%		11.2%	11.2%		14.2%	10.9%		13.0%	13.0%	
Asian	2.5%	2.4%		2.5%	2.7%		2.1%	1.9%		2.0%	2.0%	
Hispanic	10.4%	9.5%		10.2%	10.1%		9.7%	8.7%		9.4%	9.3%	
Unknown	8.7%	8.7%		8.7%	8.7%		9.1%	8.7%		8.9%	9.0%	
Medicare advantage enrolment, %col	67.8%	68.3%	0.01	67.9%	67.8%	−0.00	65.0%	64.6%	−0.01	64.9%	65.1%	0.00
Residence in long‐term care or hospitalization on index date, %col	9.5%	15.3%	**0.17**	10.7%	10.7%	−0.00	17.3%	27.1%	**0.24**	20.6%	20.8%	0.01
Diseases and frailty in 1 year prior to index date, %col												
Atrial fibrillation	7.8%	7.9%	0.00	7.9%	7.9%	0.00	8.7%	9.0%	0.01	8.9%	9.0%	0.00
SCA/VA	0.5%	0.7%	0.02	0.6%	0.6%	0.00	0.9%	1.1%	0.02	1.0%	1.0%	−0.00
Heart failure	14.4%	16.5%	0.06	14.9%	15.3%	0.01	24.9%	26.0%	0.02	25.5%	26.0%	0.01
Hypertension	67.2%	66.9%	−0.01	67.2%	67.3%	0.00	70.4%	71.2%	0.02	70.8%	70.9%	0.00
CKD	22.6%	18.8%	−0.09	21.9%	22.0%	0.04	28.2%	21.8%	**−0.15**	26.1%	26.1%	0.00
Stroke	5.1%	5.4%	0.01	5.2%	5.3%	0.01	5.2%	5.7%	0.02	5.5%	5.5%	0.00
Cirrhosis	1.7%	0.9%	−0.07	1.5%	1.6%	0.01	2.9%	1.1%	**−0.13**	2.3%	2.4%	0.00
Ascites	1.9%	1.5%	−0.04	1.8%	1.9%	0.00	3.6%	1.8%	**−0.11**	2.9%	3.0%	0.00
Diabetes insipidus	0.1%	0.0%	−0.00	0.1%	0.0%	−0.00	0.1%	0.1%	−0.01	0.1%	0.1%	−0.005
Oedema	24.7%	26.5%	0.04	25.1%	25.4%	0.01	25.6%	25.0%	−0.01	25.6%	25.7%	0.00
Glaucoma	8.7%	8.1%	−0.02	8.6%	8.6%	0.00	7.8%	7.4%	−0.01	7.7%	7.7%	0.00
Nocturia	2.9%	2.8%	−0.01	2.8%	2.8%	0.00	2.8%	2.7%	−0.00	2.7%	2.8%	0.00
Osteoporosis	5.7%	6.3%	0.02	5.9%	5.9%	0.00	4.2%	4.5%	0.02	4.3%	4.4%	0.00
Pulmonary congestion and hypostasis or pulmonary oedema	4.3%	6.0%	0.08	4.7%	4.8%	0.00	7.7%	9.9%	0.08	8.7%	8.9%	0.01
Nephrolithiasis	3.1%	2.9%	−0.01	3.0%	3.1%	0.01	3.2%	3.0%	−0.01	3.1%	3.2%	0.00
Metabolic alkalosis	0.3%	0.3%	0.01	0.3%	0.3%	0.00	0.5%	0.5%	0.00	0.5%	0.5%	0.00
Cushing's syndrome	0.1%	0.1%	0.01	0.1%	0.1%	0.00	0.1%	0.1%	0.00	0.1%	0.1%	0.00
Hyperaldosteronism	0.1%	0.0%	−0.01	0.1%	0.1%	−0.00	0.1%	0.0%	−0.02	0.1%	0.1%	0.00
Adrenogenital disorders	0.0%	0.0%	−0.00	0.0%	0.0%	−0.00	0.0%	0.0%	0.00	0.0%	0.0%	−0.00
Other corticoadrenal overactivity or ACTH‐producing bronchogenic tumour	0.0%	0.0%	0.00	0.0%	0.0%	0.00	0.0%	0.0%	−0.00	0.0%	0.0%	−0.00
Hyperthyroidism	1.5%	1.5%	−0.00	1.5%	1.4%	−0.00	1.4%	1.4%	0.00	1.4%	1.4%	0.00
Pyloric stenosis	0.1%	0.1%	0.00	0.1%	0.1%	0.00	0.1%	0.1%	−0.00	0.1%	0.1%	−0.00
Alcoholism or delirium tremens	1.9%	1.7%	−0.01	1.8%	1.8%	0.00	3.0%	2.3%	−0.04	2.7%	2.8%	0.00
Leukaemia	0.5%	0.6%	0.00	0.6%	0.6%	0.00	0.5%	0.5%	−0.00	0.5%	0.5%	0.00
Systemic lupus erythematosus	0.7%	0.7%	−0.00	0.7%	0.7%	0.00	0.7%	0.6%	−0.01	0.7%	0.7%	0.01
Amyloidosis	0.1%	0.1%	−0.01	0.1%	0.1%	0.00	0.1%	0.1%	−0.01	0.1%	0.1%	−0.00
Corticoadrenal insufficiency	0.3%	0.3%	0.00	0.3%	0.3%	0.00	0.3%	0.3%	0.00	0.3%	0.3%	0.00
Hyperosmolality	0.7%	0.8%	0.01	0.7%	0.8%	0.00	0.9%	0.9%	−0.00	1.0%	1.0%	0.00
Acidosis	2.1%	2.2%	0.01	2.1%	2.2%	0.00	3.4%	3.0%	−0.02	3.3%	3.3%	0.00
Obstructive uropathy	0.7%	0.6%	−0.00	0.6%	0.6%	−0.00	0.8%	0.6%	−0.01	0.7%	0.8%	0.00
Sickle cell disease	0.0%	0.0%	−0.01	0.0%	0.0%	0.00	0.0%	0.0%	−0.00	0.0%	0.0%	0.00
HIV/AIDS	0.2%	0.2%	−0.01	0.2%	0.2%	0.00	0.3%	0.2%	−0.02	0.3%	0.2%	−0.00
Renal transplantation	0.2%	0.1%	−0.04	0.2%	0.2%	−0.00	0.4%	0.1%	−0.07	0.3%	0.3%	0.00
Periodic paralysis	0.0%	0.0%	−0.00	0.0%	0.0%	0.00	0.0%	0.0%	−0.00	0.0%	0.0%	−0.00
Disorders of magnesium metabolism	1.6%	1.7%	0.01	1.6%	1.7%	0.01	2.0%	1.8%	−0.01	1.9%	2.0%	0.00
Claims‐based frailty index, mean (SD)	0.19 (0.07)	0.19 (0.07)	0.06	0.19 (0.08)	0.19 (0.14)	0.01	0.19 (0.07)	0.19 (0.06)	0.01	0.19 (0.08)	0.19 (0.11)	0.01
Drug markers of diseases in1 year prior to index date, %col												
ACEI/ARB	52.7%	49.2%	−0.07	51.9%	51.9%	−0.00	53.3%	51.8%	−0.03	52.8%	52.8%	−0.00
Aliskiren	0.2%	0.2%	−0.01	0.2%	0.2%	−0.00	0.3%	0.3%	−0.01	0.3%	0.3%	0.00
Potassium‐sparing diuretics	4.5%	5.7%	0.05	4.8%	4.8%	0.00	4.8%	6.4%	0.07	5.4%	5.4%	0.00
Aldosterone antagonists	3.3%	1.8%	−0.10	2.9%	2.9%	−0.00	4.6%	2.1%	**−0.14**	3.7%	3.7%	0.00
Beta‐2 agonists	21.3%	21.7%	0.01	21.4%	21.6%	0.00	21.0%	21.0%	−0.00	21.1%	21.2%	0.00
Anorexiants/antiobesity agents	0.1%	0.1%	−0.01	0.1%	0.1%	0.00	0.1%	0.1%	−0.01	0.1%	0.1%	0.00
Antiadrenergic agents	16.0%	15.6%	−0.01	16.0%	16.0%	0.00	17.4%	16.5%	−0.02	17.2%	17.2%	0.00
Antiarrhythmics, type I, except lidocaine and phenytoin	0.9%	1.2%	0.03	1.0%	1.0%	0.00	0.8%	1.2%	0.03	1.0%	1.0%	−0.00
Antiarrhythmics, type III	3.3%	4.0%	0.04	3.5%	3.6%	0.01	3.7%	5.0%	0.07	4.3%	4.3%	−0.00
Beta blockers, systemic	40.9%	40.7%	−0.01	40.9%	40.9%	−0.00	42.7%	43.3%	0.01	43.1%	42.9%	−0.00
Calcium channel blockers, dihydropyridines	26.1%	23.6%	−0.06	25.6%	25.7%	0.00	26.4%	23.3%	−0.07	25.3%	25.2%	−0.00
Calcium channel blocker, non‐dihydropyridines	7.2%	8.0%	0.03	7.4%	7.4%	0.00	7.4%	8.3%	0.03	7.8%	7.8%	−0.00
Antidiabetic agents	21.8%	19.7%	−0.05	21.3%	21.3%	−0.00	25.3%	23.2%	−0.05	24.6%	24.6%	0.00
Insulin	8.3%	6.8%	−0.06	8.0%	8.1%	0.00	11.6%	8.7%	−0.09	10.6%	10.6%	−0.00
Warfarin	8.3%	9.7%	0.05	8.6%	8.7%	0.00	9.0%	10.1%	0.04	9.6%	9.6%	−0.00
DOAC	5.0%	4.9%	−0.01	5.0%	5.0%	0.00	4.6%	4.5%	−0.00	4.6%	4.6%	0.00
Corticosteroids, inhaled	18.9%	18.9%	0.00	18.9%	19.1%	0.00	17.2%	17.3%	0.00	17.2%	17.4%	0.00
Corticosteroids, oral	25.9%	26.5%	0.01	26.1%	26.4%	0.01	24.1%	24.5%	0.01	24.4%	24.5%	0.00
Digoxin, oral	2.7%	3.3%	0.03	2.8%	2.9%	0.00	3.2%	3.5%	0.02	3.3%	3.3%	0.00
Immunosuppressants for organ transplant	1.8%	1.5%	−0.02	1.7%	1.8%	0.00	1.8%	1.2%	−0.05	1.6%	1.6%	0.00
Lipid‐lowering agents	49.3%	48.4%	−0.02	49.2%	49.1%	−0.00	48.4%	50.3%	0.04	49.2%	49.2%	−0.00
Nitrates	7.2%	8.3%	0.04	7.4%	7.5%	0.00	8.2%	10.4%	0.08	9.0%	9.0%	−0.00
Vasodilators, non‐nitrates	3.3%	2.8%	−0.03	3.2%	3.2%	0.00	4.5%	3.1%	−0.07	4.0%	4.1%	0.00
Thyroid hormones	18.6%	19.2%	0.02	18.7%	18.7%	−0.00	15.8%	16.1%	0.01	16.0%	16.0%	−0.00
Xanthine oxidase inhibitors	4.4%	3.7%	−0.03	4.3%	4.3%	0.00	5.4%	4.2%	−0.06	5.0%	5.0%	0.00
Antiglaucoma agents, ophthalmic	6.6%	6.3%	−0.01	6.5%	6.5%	−0.00	5.8%	5.6%	−0.01	5.8%	5.7%	−0.00
Antiglaucoma agents, oral	0.3%	0.3%	−0.01	0.3%	0.3%	0.00	0.3%	0.3%	−0.00	0.3%	0.3%	0.00
Bone protective drugs	5.8%	6.7%	0.04	6.0%	6.0%	−0.00	4.2%	5.0%	0.04	4.5%	4.5%	−0.00
Potassium laboratory tests in 30 days prior to index date, %col	32.3%	32.4%	0.00	32.3%	32.2%	−0.00	31.9%	32.5%	0.01	32.1%	31.8%	−0.01
Hospitalization in 30 days prior to index date, %col	17.5%	24.6%	**0.18**	19.0%	19.4%	0.01	27.4%	38.7%	**0.24**	31.4%	31.7%	0.01

Abbreviations: ACEI/ARB: angiotensin converting enzyme inhibitor/angiotensin II receptor antagonist; ACTH: adrenocorticotropic hormone; CKD: chronic kidney disease; DOAC: direct‐acting oral anticoagulant; HIV/AIDS: human immunodeficiency virus*/*acquired immunodeficiency syndrome; IPTW, inverse probability of treatment weighting; K+: potassium; SCD/VA, sudden cardiac arrest/ventricular arrhythmia; SD: standard deviation; SDiff: standardized difference.

^a^ Bolded values indicate meaningful imbalance (|standardized difference| > 0.1).

^b^ Weighted sample size.

The hdPS model included 662 covariates (165 pre‐defined and 497 empirically identified) in each furosemide dose cohort (Tables S4 and S5). After IPTW, all baseline characteristics were balanced between subjects with and without empiric potassium (Table 1), and there were good PS overlaps within each furosemide dose cohort (Figures S2 and S3). PS trimming further improved the balance (Table S6). For both furosemide doses, baseline characteristics of persons removed by trimming were quite different from those remaining cohorts after trimming (Table S7 **)**.

### 3.2 Primary and sensitivity analyses

#### 3.2.1 Primary outcome: all‐cause mortality

In furosemide <40 and ≥40 mg/day cohorts, crude mortality rates were 85.2 (95% confidence interval [CI] 84.7–85.7) and 91.3 (90.5–91.8) per 1000 person‐years, respectively. As‐started IPTW‐adjusted Kaplan–Meier curves (Figures S4 and S5) showed similar survival probability between people with *vs*. without empiric potassium throughout follow‐up in both furosemide dose cohorts. Visual inspection of Kaplan–Meier curves did not suggest a violation of the proportional‐hazards assumption. As‐started IPTW‐hazard ratios (IPTW‐HRs) for empiric potassium (*vs*. no potassium) were similar in both furosemide cohorts, that is, 1.02 (1.01–1.04) for <40 mg/day and 1.02 (1.00–1.03) for ≥40 mg/day. Findings from the prespecified sensitivity analyses that trimmed the PS distribution were attenuated to the null (Table 2). IPTW HRs from the as‐treated analyses were nearly identical to and had 95% CIs that overlapped the as‐started findings (Table 2).

**TABLE 2:** IPTW Cox proportional hazards models for all outcomes among individuals initiating furosemide <40 mg/day or ≥40 mg/day with *vs.* without empiric potassium.

Outcome			Furosemide <40 mg/day		Furosemide ≥40 mg/day	
	Analysis		Crude HR (95%CI)	IPTW‐HR (95%CI)	Crude HR (95%CI)	IPTW‐HR (95%CI)
All‐cause mortality	As‐started analysis	Full^a^ cohort	1.07 (1.06–1.09)	1.02 (1.01–1.04)	0.95 (0.93–0.96)	1.02 (1.00–1.03)^1^
	As‐treated analysis		1.09 (1.05–1.14)	1.00 (0.95–1.05)	0.95 (0.91–0.99)	0.97 (0.92–1.02)
	As‐started analysis	Trimmed^b^ sample	1.10 (1.08–1.12)	1.01 (1.00–1.03)^2^	1.06 (1.04–1.07)	1.00 (0.98–1.02)
	As‐treated analysis		1.08 (1.03–1.13)	0.99 (0.94–1.04)	1.01 (0.96–1.06)	0.94 (0.90–0.99)
SCA/VA	As‐started analysis	Full^a^ cohort	0.97 (0.93–1.01)	0.99 (0.94–1.04)	0.88 (0.85–0.92)	0.98 (0.94–1.03)
	As‐treated analysis		1.04 (0.95–1.15)	1.03 (0.91–1.15)	0.95 (0.88–1.04)	0.97 (0.88–1.07)
	As‐started analysis	Trimmed^b^ sample	1.00 (0.95–1.05)	0.99 (0.95–1.04)	0.96 (0.92–1.00)^3^	0.97 (0.93–1.01)
	As‐treated analysis		1.02 (0.92–1.13)	0.99 (0.89–1.11)	0.96 (0.88–1.05)	0.96 (0.87–1.06)
Stroke	As‐started analysis	Full^a^ cohort	1.03 (1.01–1.06)	1.03 (1.00–1.06)^4^	0.97 (0.95–1.00)^5^	1.01 (0.98–1.04)
	As‐treated analysis		1.08 (1.01–1.15)	1.04 (0.97–1.13)	1.11 (1.04–1.18)	1.06 (0.99–1.15)
	As‐started analysis	Trimmed^b^ sample	1.05 (1.02–1.08)	1.02 (0.99–1.05)	1.03 (1.00–1.06)^6^	1.00 (0.97–1.04)
	As‐treated analysis		1.06 (0.99–1.13)	1.01 (0.94–1.09)	1.11 (1.03–1.19)	1.07 (1.00–1.16)^7^

Abbreviations: CI: confidence interval; HR: hazard ratio; IPTW: inverse probability treatment weighting; SCA/VA: sudden cardiac arrest/ventricular arrhythmia.

^a^ Sample sizes *n* = 511 532 for full furosemide <40 mg/day cohort and *n* = 320 703 for full furosemide ≥40 mg/day cohort.

^b^ Sample sizes *n* = 445 778 for trimmed furosemide <40 mg/day cohort and *n* = 270 766 for trimmed furosemide ≥40 mg/day cohort.

^1^ *p* = .0671

^2^ *p* = .1453

^3^ *p* = .0310

^4^ *p* = .0354

^5^ *p* = .0467

^6^ *p* = .0752

^7^ *p* = .0667

#### 3.2.2 Secondary outcomes: SCA/VA and stroke

See details in Supporting Information Results, Table 2, and Figures S4 and S5.

### 3.3 Additional subgroup and IV analyses

See details in Supporting Information Results and Figures S6 and S7.

## 4 DISCUSSION

In this series of retrospective cohort studies using a large U.S. commercial health insurance database, most analyses found no association and a minority of analyses identified extremely modest positive associations between the use of empiric potassium supplementation and outcomes of interest—findings that appear to be potentially sensitive to employed analytic approaches and susceptible to residual confounding. Among furosemide <40 mg/day initiators, we identified in as‐started analyses an extremely small increase in mortality (IPTW‐HR 1.02, 95% CI 1.01–1.04) and stroke (IPTW‐HR 1.03, 1.00–1.06; *p* = .0354) rates among people with (*vs*. without) empiric potassium. We identified no associations with SCA/VA in any analyses, or mortality or stroke in as‐treated analyses and sensitivity analyses of the trimmed sample. Among furosemide ≥40 mg/day initiators, we identified no associations between empiric potassium and any outcome across analyses, except for a modest reduction in mortality rate in an as‐treated analysis of the trimmed sample (IPTW‐HR 0.94, 0.90–0.99).

Results were highly consistent across analyses for the mortality outcome. We only found a very modest elevated HR for death associated with empiric potassium in the as‐started analysis in the <40 mg/day furosemide cohort (IPTW‐HR 1.02, 1.01–1.04), which is of uncertain clinical relevance. This finding differed from a previous study using Medicaid data from economically disadvantaged Americans, which found a protective association between potassium and mortality in both furosemide <40 and ≥40 mg/day (matched HRs 0.93, 0.86–1.00; *p* = .05 and .84, .79–.89, respectively).^6^

Potassium is naturally present in many foods and available without prescription as a dietary supplement.^31^ Commercially insured Americans are generally wealthier and have less comorbidity than the Medicaid population, which might explain the differing findings with the prior study by Leonard et al.^6^ Individuals in our study sample might obtain sufficient potassium from their diet and/or have better access to potassium supplements, making additional supplementation with prescription potassium potentially unnecessary for those taking lower doses (<40 mg/day) of furosemide. Krogager and colleagues found that even the lower or higher end of the normal range of serum potassium level (3.5–3.8 or 4.6–5.0 mmol/L) was associated with an increased risk of mortality in HF patients requiring diuretic treatment following myocardial infarction, suggesting that there might be a very delicate balance between optimal potassium levels to improve survival in specific patient populations. Potassium levels slightly outside the normal range can be detrimental.^32^ Previous studies have reported an association between hyperkalaemia and mortality.^33^, ^34^ Clinical guidelines have suggested that serum potassium levels have a U‐shaped relationship with mortality in individuals with HF, such that both hypokalaemia and hyperkalaemia are associated with higher risk.^10^ Hypokalaemia has been linked to a higher risk of VA, sudden cardiac death, and all‐cause mortality, likely because of enhanced myocardial excitability and electrical instability.^4^, ^35^, ^36^ Conversely, hyperkalaemia has been associated with increased all‐cause and cardiovascular mortality, bradyarrhythmias, conduction disturbances, HF hospitalizations, and may also contribute to peripheral neuropathy and renal tubular acidosis.^35^, ^37^, ^38^ Several large observational studies have demonstrated that these risks extend beyond extreme potassium values and may be observed even within the lower (3.5–4.1 mmol/L) and upper (4.8–5.0 mmol/L) ends of the conventional normal range of potassium levels, with the lowest risk of death observed in a relatively narrow ‘mid‐normal’ range of approximately 4–5 mmol/L and an optimal value around 4.2 mmol/L.^39^, ^40^, ^41^ Trial‐based analyses also showed consistent patterns in severe HF populations, reinforcing that both extremes could carry risk.^42^ As a result, continuous and careful monitoring of potassium levels, renal function and diuretic dosing should be performed regularly, especially in people co‐prescribed renin‐angiotensin‐aldosterone system (RAAS) inhibitors that can potentially increase hyperkalaemia risk.^9^, ^10^, ^11^

The small protective association observed for the cohort of furosemide ≥40 mg/day after trimming in the as‐treated analysis (IPTW‐HR 0.94, 0.90–0.99) may suggest that empiric potassium is modestly beneficial in a more homogenous population of furosemide users—those who required a higher dose of furosemide but were less sick than persons trimmed from the analysis. However, characteristics associated with individual decisions or reasons to discontinue or switch from furosemide to another alternative might influence the outcomes, which we could not account for in as‐treated analyses.^43^

We found no association between empiric potassium supplementation and SCA/VA in either furosemide dose cohorts across analyses, similar to previous findings by Leonard et al.^6^ and Cooper et al.^44^ A prior study by Harkness et al. reported that in the acute care setting, there was no association between correcting hypokalaemia and arrhythmias.^45^ In another study among Swedes with chronic HF, Ekundayo et al. suggested that potassium supplements, while helpful to correct hypokalaemia, might not provide any additional mortality reduction benefit, and aldosterone antagonists may be preferable to maintain normokalaemia in the long term.^46^ Of note, previous studies have shown that potassium‐sparing diuretics, but not potassium supplements, were associated with reduced cardiac arrest risk.^47^

We observed a very modest increased rate of stroke with the as‐started analysis in the <40 mg/day furosemide cohort (IPTW‐HR 1.03, 95% CI 1.00–1.06; *p* = .0354), a magnitude unlikely to translate into a clinically meaningful effect and was not observed across other analytical approaches. This finding was somewhat consistent with results from prior studies reporting associations between dyskalaemia—particularly hypokalaemia—and ischaemic stroke risk, which may partially contextualize this finding.^48^ In contrast, randomized evidence from a cluster trial demonstrated lower stroke rates among older adults with hypertension and history of stroke who used potassium‐containing salt substitutes, highlighting that the effects of potassium on stroke risk may depend on population characteristics and the form of potassium exposure.^7^ Although a substantial proportion of our study cohort had a history of hypertension, we lacked information on dietary modifications or salt substitutions, which could negate any stroke‐related effects of empiric potassium supplementation. Consistent with this heterogeneity, prior work by Fang et al. only found an association between higher dietary potassium intake and reduced stroke mortality in specific subgroups, such as among Black men and hypertensive men.^49^ Nevertheless, multiple meta‐analyses of randomized trials have shown that potassium supplementation lowers systolic and diastolic blood pressure—particularly among those with hypertension—and higher potassium intake has also been associated with a reduced risk of stroke, suggesting that potential benefits of potassium supplementation should not be overlooked in appropriately selected populations.^50^

Our study had strengths. We used 12 years of data among a large, diverse sample of commercially insured Americans newly starting furosemide, which resulted in narrow confidence intervals. We used a semi‐automated data‐adaptive approach for covariate selection to minimize residual confounding.^18^, ^19^, ^20^ Moreover, we conducted several prespecified sensitivity and subgroup analyses to compare findings based on different analytical approaches.

Our study had limitations. Our exposure definition, by design, was intentionally restricted to empiric potassium supplementation proactively prescribed on the same date as furosemide initiation. This design choice, while reducing the potential for confounding, prevented us from capturing potassium prescribed later in response to laboratory findings or a diagnosis of hypokalaemia, and may attenuate true associations between potassium supplementation and outcomes towards the null, particularly given the substantial potassium coverage observed among empiric users in as‐treated analyses and the minimal crossover observed among non‐users. Moreover, while pharmacy dispensings do not guarantee actual medication consumption, this assumption is generally considered acceptable in pharmacoepidemiologic studies, especially of chronically administered therapies. We could not identify the potassium intake or the intake of other electrolytes that can affect potassium levels (e.g. magnesium)^51^ from sources such as diet or over‐the‐counter (OTC) medications in claims data; thus, individuals might be misclassified, and the impact of other electrolyte supplementation on potassium levels could not be accounted for. Absorption of oral furosemide products is known to be erratic with considerable inter‐ and intra‐subject variability,^52^ which can affect any effect of empiric potassium if it exists. Potassium‐prescribing decisions are often guided by clinical factors not fully captured in claims data, such as baseline serum potassium levels, CKD severity and progression, and the frequency and timing of laboratory monitoring. These factors may influence clinicians' decisions to initiate or withhold empiric potassium supplementation, thereby channelling patients towards or away from empiric use—particularly in those at higher risk of hyperkalaemia—and may contribute to residual and/or unmeasured confounding and potential model misspecification,^28^ even with adequate covariate balance achieved through IPTW and adjustment for measured confounders. Consequently, empiric potassium recipients may have had greater baseline risk, which could plausibly explain the very small, elevated hazard ratios observed in some as‐started analyses (e.g. mortality HR 1.02 in the <40 mg/day furosemide cohort). Importantly, despite the large number of covariates included in the IPTW, covariate balance was achieved, and the magnitude of these associations was extremely modest across analytic approaches, suggesting that residual bias or model imprecision is unlikely to mask a clinically meaningful effect. Furthermore, we did not adjust for time‐varying covariates in the as‐treated models, which is potentially important if reasons for discontinuation were related to the risk for study outcomes (e.g., sicker patients stopped potassium supplementation). Similarly, findings in subgroup analyses should be interpreted carefully, as analyses in other populations with different distributions of these variables may result in very different estimates.^53^ In the presence of effect‐measure modification, the IPTW analytic approach can produce different summary estimates compared to other methods, such as PS matching, although both approaches allow for causal contrasts.^54^ Our decision to select the last day of the month as the death date (because of the unavailability of the specific death date in the database) could potentially affect as‐treated analyses, where patients could be censored owing to furosemide discontinuation/switching, and their death (which might have occurred any time before the last day of the month) will not be counted. Lastly, we lacked information on the cause of death or complete potassium laboratory values in our databases; therefore, individuals could have died because of reasons entirely unrelated to empiric potassium supplementation or dyskalaemia.

In this study of commercially insured new users of furosemide, we found only modest and analytically sensitive associations between empiric potassium supplementation and outcomes, the clinical relevance of which remains uncertain. These findings should be interpreted cautiously given the observational study design and potential for residual confounding, and decisions regarding empiric supplementation should continue to be individualized based on patient‐specific risk factors and clinical judgement. Given the high prevalence of furosemide prescriptions among highly heterogeneous patient populations, a large pragmatic trial may be warranted to more definitively evaluate the potential benefits and harms of empiric potassium supplementation among furosemide initiators.

## AUTHOR CONTRIBUTIONS

Thanh Phuong Pham Nguyen, Sean Hennessy, Warren B. Bilker, Laura M. Dember, Todd A. Miano, Allison W. Willis and Charles E. Leonard designed the research. Thanh Phuong Pham Nguyen and Colleen M. Brensinger performed the research. Thanh Phuong Pham Nguyen, Sean Hennessy, Laura M. Dember, Todd A. Miano, Tat‐Thang Vo, Allison W. Willis and Charles E. Leonard analysed the data. Thanh Phuong Pham Nguyen wrote the manuscript; all other authors revised important content of the manuscript. All authors reviewed and approved the manuscript.

## CONFLICT OF INTEREST STATEMENT

Dr. T.P.P.N. was a member of the 2022–2023 Junior Investigator Intensive Program of the U.S. Deprescribing Research Network, which was funded by the National Institute on Aging (Grant # R24AG064025), and receives support from the National Institutes of Health (Grants #1RF1NS132673‐01, #R01NS099129, #R01AG02515215, #R01AG06458903, #R33AG086944) and Acadia Pharmaceuticals Inc. Dr. S.H. has consulted for Novo Nordisk, Arbor Pharmaceuticals, the Medullary Thyroid Cancer Consortium (Novo Nordisk and Eli Lilly), Biogen, Intercept Pharmaceuticals, Provention Bio, bluebird bio, and Amylyx Pharmaceuticals, and is a special government employee of the U.S. Food and Drug Administration. Dr. L.M.D. has received compensation from the National Kidney Foundation for her role as Deputy Editor of the *American Journal of Kidney Diseases*, honoraria from the American Society of Nephrology, consulting fees from AstraZeneca, Merck, and Alucent Biomedical, and compensation for serving on Data and Safety Monitoring Boards for the National Institute of Diabetes and Digestive and Kidney Diseases, CSL Behring, and Vertex Pharmaceuticals. Dr. T.A.M. receives financial support from the National Institute of Health (Grant # K08DK124658). Dr. A.W. receives financial support from the National Institutes of Health (Grants #1RF1NS132673‐01, #K24AG075234, #P30AG059302), the Parkinson's Foundation, Acadia Pharmaceuticals Inc. and the University of Pennsylvania. Dr. C.E.L. receives financial support, as Principal Investigator or Site Principal Investigator, from the National Institute on Aging (Grants #2R01AG060975, #5R01AG077620). Dr. Leonard recently received honoraria from the American College of Clinical Pharmacy Foundation, the Consortium for Medical Marijuana Clinical Outcomes Research and Ancora Education. Dr. C.E.L. is a Special Government Employee of the U.S. Food and Drug Administration and has consulted for their Reagan–Udall Foundation. Dr. C.E.L. has consulted for TriNetX, Novo Nordisk and Moderna. Dr. C.E.L.'s spouse is an employee of Johnson & Johnson; neither Dr. C.E.L. nor his spouse owns stock in the company. All other authors declared no competing interests for this work.

## Supporting information


**Figure S1.** Illustration of study design.

**Figure S2.** Propensity score overlap before (histograms) and after IPTW (density plots) among individuals initiating furosemide <40 mg/day with and without empiric potassium†.

**Figure S3.** Propensity score overlap before (histograms) and after IPTW (density plots) among individuals initiating furosemide ≥40 mg/day with and without empiric potassium†.

**Figure S4.** IPTW‐adjusted Kaplan–Meier curves for outcomes of interest among initiators of furosemide <40 mg/day.
Figure S4a. All‐cause mortality outcome.
Figure S4b. SCA/VA outcome.
Figure S4c. Stroke outcome.

**Figure S5.** IPTW‐adjusted Kaplan–Meier curves for outcomes of interest among initiators of furosemide ≥40 mg/day.
Figure S5a. All‐cause mortality outcome.
Figure S5b. SCA/VA outcome.
Figure S5c. Stroke outcome.

**Figure S6.** Forest plot† examining effect modification among initiators of furosemide <40 mg/day by subgroups.

**Figure S7.** Forest plot† examining effect modification among initiators of furosemide ≥40 mg/day by subgroups.

**Table S1.** Specifications used in the empiric identification of covariates for high‐dimensional propensity score inclusion.

**Table S2.** Pre‐specified covariates included in the high‐dimensional propensity score.

**Table S3.** Operational definition for the composite secondary outcomes of interest.

**Table S4.** Covariates empirically identified by the high‐dimensional propensity score method for the <40 mg/day furosemide cohort.

**Table S5.** Covariates empirically identified by the high‐dimensional propensity score method for the ≥40 mg/day furosemide cohort.

**Table S6.** Baseline characteristics among individuals initiating furosemide <40 mg/day or ≥40 mg/day with and without empiric potassium in the remaining cohort after PS trimming.

**Table S7.** Baseline characteristics among trimmed individuals (<2.5th percentile or >97.5th percentile) *vs.* full remaining cohort.

**Table S8.** IPTW Cox proportional hazards models for all outcomes among individuals initiating furosemide <40 mg/day or ≥40 mg/day with *vs.* without empiric potassium at six‐month and one‐year follow‐up†.

**Data S1.** Supporting Information.

**Data S2.** Supporting Information.

## Data Availability

The data that support the findings of this study are not publicly available due to privacy, commercial or ethical restrictions.
