# Supplementary material for: Impact of empiric potassium supplementation on mortality, sudden cardiac arrest and stroke in furosemide initiators
Source: Br J Clin Pharmacol. 2026 May 3;92(8):2924–36. doi: 10.1002/bcp.70584 (PMC13421057; doi:10.1002/bcp.70584)
Supplement: Supplementary file 1 — Figure S1. Illustration of study design. [file BCP-92-2924-s009.docx]

**Figure S1. Illustration of study design**

**Cohort entry date**

**(First f*urosemide* Rx [± empiric potassium Rx])**

**Day 0**

**Exclusion assessment window**

**(Intermittent medical and drug coverage^a^)**

**Days [-365, -1]**

**Washout window (exposure)**

**(No furosemide or other loop diuretic Rx, or empiric potassium Rx)**

**Days [-365, -1]**

**Covariate assessment window**

**(demographic, state, calendar year, Medicare Advantage, long-term care residence, or hospitalization on index)**

**Days [0, 0]**

**Covariate assessment window**

**(Baseline covariates^b^)**

**Days [-365 or -30 , -1]**

**Follow-up window**

**Days [0, Censor^c^]**

**Time**

1. Up to 30-day gaps in insurance coverage allowed; patients with a prescription for potassium supplement, a baseline diagnosis of hypokalemia, hyperkalemia, or dialysis-dependent chronic kidney disease excluded.
2. Baseline covariates included: diseases (e.g., atrial fibrillation, hypertension), claims-based frailty index, drug markers of chronic diseases (e.g., antihypertensive agents), potassium laboratory test measured within 30 days prior to index, hospital admission within 30 days prior to index, measures of intensity of healthcare utilization, and other covariates selected via the hdPS algorithm.
3. Earliest of: (1) an outcome of interest (death, sudden cardiac arrest/ventricular arrhythmia [SCA/VA], stroke), (2) death (for the SCA/VA and stroke analyses), (3) end of database (December 31, 2019) for primary as-started analyses; additional censoring criteria for as-treated secondary analyses included: (4) switching to another diuretic and (5) furosemide discontinuation (i.e., no new furosemide dispensing after permitting a grace period corresponding to 20% of the days’ supply of the latest furosemide Rx).

hdPS: high-dimensional propensity score; Rx: prescription
