# Supplementary material for: Impact of empiric potassium supplementation on mortality, sudden cardiac arrest and stroke in furosemide initiators
Source: Br J Clin Pharmacol. 2026 May 3;92(8):2924–36. doi: 10.1002/bcp.70584 (PMC13421057; doi:10.1002/bcp.70584)
Supplement: Supplementary file 3 — Figure S3. Propensity score overlap before (histograms) and after IPTW (density plots) among individuals initiating furosemide ≥40 mg/day with and without empiric potassium†. [file BCP-92-2924-s013.docx]

**Figure S3. Propensity score overlap before (histograms) and after IPTW (density plots) among individuals initiating furosemide ≥40 mg/day with and without empiric potassium^†^**

**
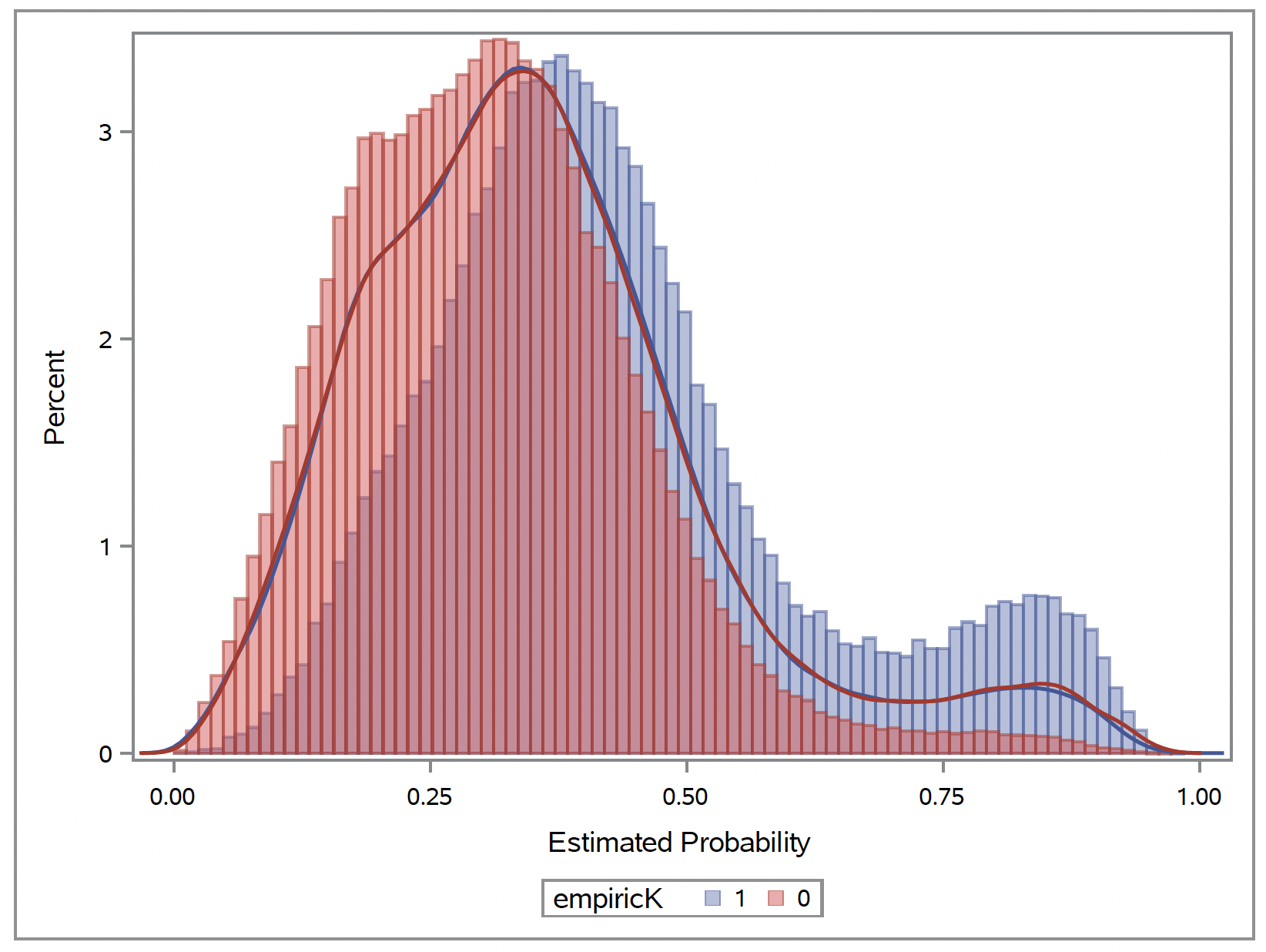
**

IPTW: inverse probability of treatment weighting; empiricK: empiric potassium supplementation

^†^ Histograms represent overlap before IPTW, and density plots represent overlap after IPTW
