# Supplementary material for: Impact of empiric potassium supplementation on mortality, sudden cardiac arrest and stroke in furosemide initiators
Source: Br J Clin Pharmacol. 2026 May 3;92(8):2924–36. doi: 10.1002/bcp.70584 (PMC13421057; doi:10.1002/bcp.70584)
Supplement: Supplementary file 4 — Figure S4. IPTW‐adjusted Kaplan–Meier curves for outcomes of interest among initiators of furosemide <40 mg/day. Figure S4a. All‐cause mortality outcome. Figure S4b. SCA/VA outcome. Figure S4c. Stroke outcome. [file BCP-92-2924-s007.docx]

**Figure S4. IPTW-adjusted Kaplan-Meier curves for outcomes of interest among initiators of furosemide <40 mg/day**

**Figure S4a. All-cause mortality outcome**

p=0.0047

**Figure S4b. SCA/VA outcome**

p=0.6535

**Figure S4c. Stroke outcome**

p=0.0318

IPTW: inverse probability of treatment weighting; SCA/VA: sudden cardiac arrest/ventricular arrhythmia
