# Supplementary material for: Impact of empiric potassium supplementation on mortality, sudden cardiac arrest and stroke in furosemide initiators
Source: Br J Clin Pharmacol. 2026 May 3;92(8):2924–36. doi: 10.1002/bcp.70584 (PMC13421057; doi:10.1002/bcp.70584)
Supplement: Supplementary file 5 — Figure S5. IPTW‐adjusted Kaplan–Meier curves for outcomes of interest among initiators of furosemide ≥40 mg/day. Figure S5a. All‐cause mortality outcome. Figure S5b. SCA/VA outcome. Figure S5c. Stroke outcome. [file BCP-92-2924-s005.docx]

**Figure S5. IPTW-adjusted Kaplan-Meier curves for outcomes of interest among initiators of furosemide ≥40 mg/day**

**Figure S5a. All-cause mortality outcome**

p=0.0648

**Figure S5b. SCA/VA outcome**

p=0.3985

**Figure S5c. Stroke outcome**

p=0.5921

IPTW: inverse probability of treatment weighting; SCA/VA: sudden cardiac arrest/ventricular arrhythmia
