# Supplementary material for: Impact of empiric potassium supplementation on mortality, sudden cardiac arrest and stroke in furosemide initiators
Source: Br J Clin Pharmacol. 2026 May 3;92(8):2924–36. doi: 10.1002/bcp.70584 (PMC13421057; doi:10.1002/bcp.70584)
Supplement: Supplementary file 7 — Figure S7. Forest plot† examining effect modification among initiators of furosemide ≥40 mg/day by subgroups. [file BCP-92-2924-s003.docx]

**Figure S7. Forest plot^†^ examining effect modification among initiators of furosemide ≥40 mg/day by subgroups**

**
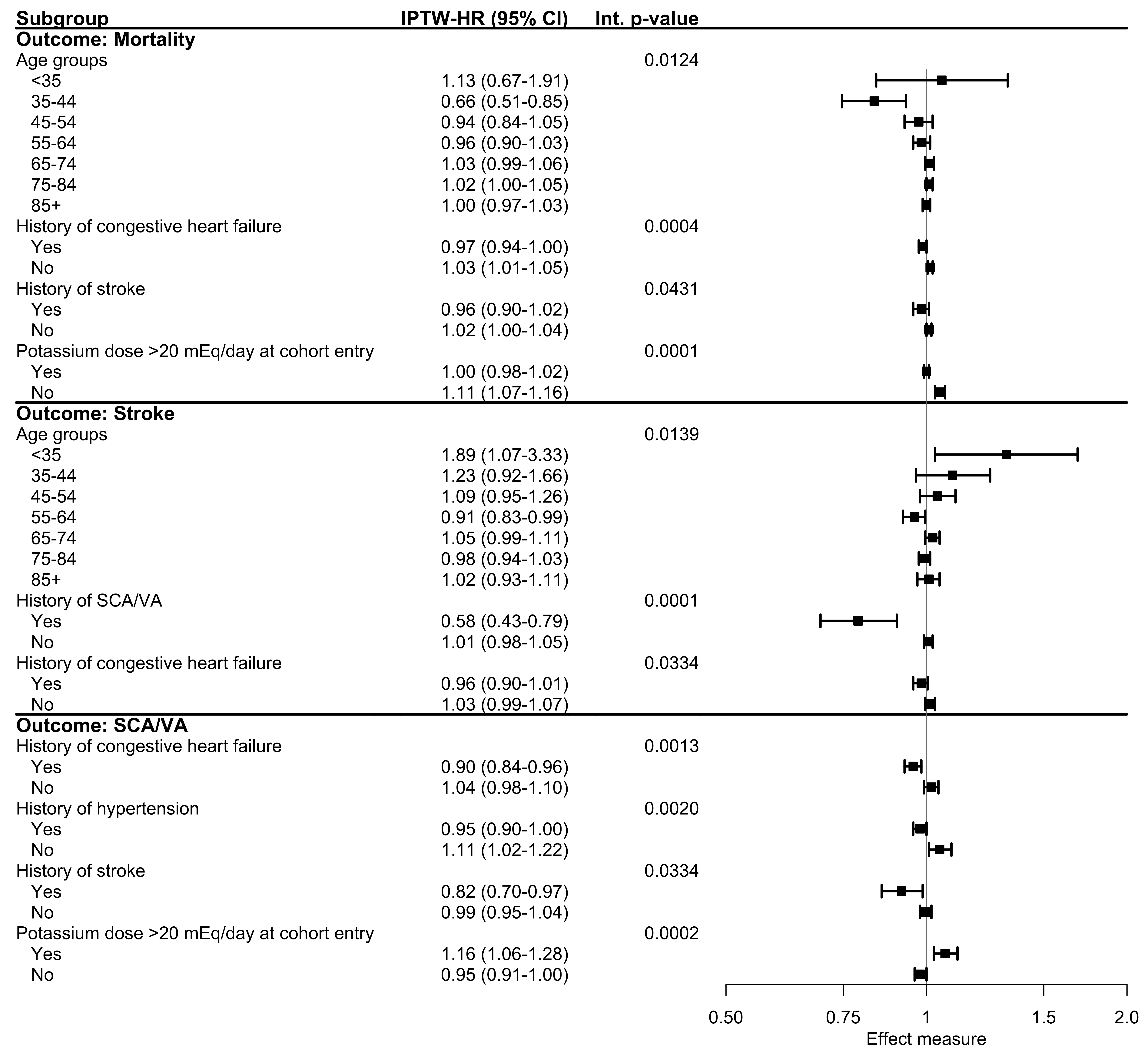
**

^†^ Outcomes are examined using as-started analyses

CI: confidence interval; IPTW-HR: inverse probability of treatment weighting hazard ratio; int. p-value: p-value for interaction; SCA/VA: sudden cardiac arrest/ventricular arrhythmia
