# Supplementary material for: Impact of empiric potassium supplementation on mortality, sudden cardiac arrest and stroke in furosemide initiators
Source: Br J Clin Pharmacol. 2026 May 3;92(8):2924–36. doi: 10.1002/bcp.70584 (PMC13421057; doi:10.1002/bcp.70584)
Supplement: Supplementary file 8 — Table S1. Specifications used in the empiric identification of covariates for high‐dimensional propensity score inclusion. [file BCP-92-2924-s004.docx]

### Table S1. Specifications used in the empiric identification of covariates for high-dimensional propensity score inclusion

| Data dimensions (*p*) | Inpatient ICD-9-CM diagnoses |
| --- | --- |
|  | Inpatient ICD-10-CM diagnoses |
|  | Inpatient ICD-9-CM procedures |
|  | Inpatient ICD-10-CM procedures |
|  | Inpatient CPT/HCPCS procedures |
|  | Laboratory LOINC observations |
|  | Outpatient ICD-9-CM diagnoses |
|  | Outpatient ICD-10-CM diagnoses |
|  | Outpatient ICD-9-CM procedures |
|  | Outpatient ICD-10-CM procedures |
|  | Outpatient CPT/HCPCS procedures |
|  | Other setting ICD-9-CM diagnoses |
|  | Other setting ICD-10-CM diagnoses |
|  | Other setting ICD-9-CM procedures |
|  | Other setting ICD-10-CM procedures |
|  | Other setting CPT/HCPCS procedures |
|  | Outpatient medications |
| Granularity of *p* | 3 digits for ICD-9-CM diagnoses, 3 characters for ICD-10-CM diagnoses, 7 characters for LOINC observations, 2 digits for ICD-9-CM procedures, 3 characters for ICD-10-PCS procedures, 5 digits for CPT, 5 characters for HCPCS, and Lexicon Plus-defined active ingredient for medications (Cerner Multum: Denver, Colorado) |
| Empiric covariates identified (*n*), per *p*, ranked in descending order by prevalence | *n* = 200 |
| Method of covariate prioritization | Bross bias formula:  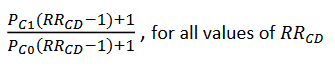  where P_C1_ represents the prevalence of the binary covariate within the group with the exposure of interest, P_C0_ the prevalence of the binary covariate within the group with the reference exposure, and RR_CD_ the relative risk for the univariate association between the binary covariate and the outcome.^6^ |
| Empiric covariates selected (*k*), across *p*, subsequent to prioritization | *k* = 500, plus the investigator-predefined covariates listed in **Table S2** |
| Software used | SAS package from <https://www.drugepi.org/dope/software> |
| CPT: Current Procedural Terminology; HCPCS: Healthcare Common Procedure Coding System; ICD-9-CM: International Classification of Diseases, Ninth Revision, Clinical Modification; ICD-10-CM: International Classification of Diseases, Tenth Revision, Clinical Modification; LOINC: Logical Observation Identifiers Names and Codes; PCS: Procedure Coding System | |
