# Supplementary material for: Impact of empiric potassium supplementation on mortality, sudden cardiac arrest and stroke in furosemide initiators
Source: Br J Clin Pharmacol. 2026 May 3;92(8):2924–36. doi: 10.1002/bcp.70584 (PMC13421057; doi:10.1002/bcp.70584)
Supplement: Supplementary file 9 — Table S2. Pre‐specified covariates included in the high‐dimensional propensity score. [file BCP-92-2924-s011.docx]

### Table S2. Pre-specified covariates included in the high-dimensional propensity score

| Demographics | Age | |
| --- | --- | --- |
|  | Sex | |
|  | Race | |
|  | State of residence | |
|  | Calendar year of cohort entry | |
|  | Enrollment in Medicare Advantage | |
|  | Residence in long-term care/ hospitalization on index date | |
| Frailty measurement | Claims-based frailty index (1) | |
| Comorbidities in one year prior to index date (day -365 through day -1) | Atrial fibrillation | Adrenogenital disorders |
|  | SCA/VA | Other corticoadrenal overactivity/ACTH-producing bronchogenic tumor |
|  | Heart failure | Hyperthyroidism |
|  | Hypertension | Pyloric stenosis |
|  | Chronic kidney disease | Alcoholism/delirium tremens |
|  | Stroke | Leukemia |
|  | Cirrhosis | Systemic lupus erythematosus |
|  | Ascites | Amyloidosis |
|  | Diabetes insipidus | Corticoadrenal insufficiency |
|  | Edema | Hyperosmolality |
|  | Glaucoma | Acidosis |
|  | Nocturia | Obstructive uropathy |
|  | Osteoporosis | Sickle cell disease |
|  | Pulmonary congestion and hypostasis/pulmonary edema | HIV/AIDS |
|  | Nephrolithiasis | Renal transplantation |
|  | Metabolic alkalosis | Periodic paralysis |
|  | Cushing’s syndrome | Disorders of magnesium metabolism |
|  | Hyperaldosteronism |  |
| Drug markers of diseases in one year prior to index date (day -365 through day -1) | ACEI/ARB | Corticosteroids, inhaled |
|  | Aliskiren | Corticosteroids, oral |
|  | Potassium-sparing diuretics | Digoxin, oral |
|  | Aldosterone antagonists | Immunosuppressants for organ transplant |
|  | Beta-2 adrenoreceptor agonists | Lipid-lowering agents |
|  | Anorexiants/antiobesity agents | Nitrates |
|  | Antiadrenergic agents | Vasodilators, non-nitrates |
|  | Antiarrhythmics, type I, except lidocaine and phenytoin | Thyroid hormones |
|  | Antiarrhythmics, type III | Warfarin |
|  | Beta blockers, systemic | Xanthine oxidase inhibitors |
|  | Calcium channel blockers, dihydropyridines | Antiglaucoma agents, ophthalmic |
|  | Calcium channel blocker, non-dihydropyridines | Antiglaucoma agents, oral |
|  | Antidiabetic agents | Bone protective drugs |
|  | Insulin | Antiretrovirals for HIV |
|  | Direct-acting oral anticoagulants |  |
| Laboratory tests within 30 days prior to index date | Presence of any tests ordered for potassium or metabolic panels including potassium within 30 days prior to index date | |
| Hospitalization within 30 days prior to index date | Presence of any hospitalization claims within 30 days prior to index date | |
| Measures of intensity of healthcare utilization | Numbers of: prescriptions dispensed; inpatient diagnoses; inpatient procedures; outpatient diagnoses; outpatient procedures; other setting diagnoses; other setting procedures; laboratory LOINC codes | |
| ACEI/ARB: angiotensin converting enzyme inhibitors/angiotensin II receptor antagonists; ACTH: adrenocorticotropic hormone; HIV/AIDS: human immunodeficiency virus/ acquired immunodeficiency syndrome; LOINC: LOINC: Logical Observation Identifiers Names and Codes; SCA/VA: sudden cardiac arrest/ventricular arrhythmia | | |

**Reference:**

(1) Kim, D.H., Schneeweiss, S., Glynn, R.J., Lipsitz, L.A., Rockwood, K. & Avorn, J. Measuring Frailty in Medicare Data: Development and Validation of a Claims-Based Frailty Index. *J Gerontol A Biol Sci Med Sci* **73**, 980-7 (2018).
