# Supplementary material for: Impact of empiric potassium supplementation on mortality, sudden cardiac arrest and stroke in furosemide initiators
Source: Br J Clin Pharmacol. 2026 May 3;92(8):2924–36. doi: 10.1002/bcp.70584 (PMC13421057; doi:10.1002/bcp.70584)
Supplement: Supplementary file 10 — Table S3. Operational definition for the composite secondary outcomes of interest. [file BCP-92-2924-s001.docx]

**Table S3. Operational definition for the composite secondary outcomes of interest**

| **Outcome of interest** | **Outcome component** | **ICD-9-CM codes** | **ICD-10-CM diagnosis codes** | **Discharge diagnosis position and claim type** |
| --- | --- | --- | --- | --- |
| Sudden cardiac arrest/ventricular arrhythmia | Sudden cardiac arrest | 427.5 | I46.9 | First listed or principal position discharge diagnosis on an emergency department claim or an inpatient hospitalization claim |
|  |  | 798.0, 798.1, 798.2 | - |  |
|  | Ventricular arrhythmia | 427.1 | I47.2 |  |
|  |  | 427.41 | I49.01 |  |
|  |  | 427.42 | I49.02 |  |
| Stroke | Ischemic stroke/TIA | 433.01, 433.11, 433.21, 433.31, 433.81, 433.91, 434.00, 434.01, 434.10, 434.11, 434.90, 434.91, 435.0, 435.1, 435.3, 435.8, 435.9, 436, 997.02 | G45.0, G45.1, G45.2, G45.8, G45.9, G46.0, G46.1, G46.2, G46.3, G46.4, G46.5, G46.6, G46.7, G46.8, G97.31, G97.32, I63.00, I63.011, I63.012, I63.013, I63.019, I63.02, I63.031, I63.032, I63.039, I63.09, I63.10, I63.111, I63.112, I63.119, I63.12, I63.131, I63.132, I63.139, I63.19, I63.20, I63.211, I63.212, I63.213, I63.219, I63.22, I63.231, I63.232, I63.233, I63.239, I63.29, I63.30, I63.311, I63.312, I63.313, I63.319, I63.321, I63.322, I63.323, I63.329, I63.331, I63.332, I63.333, I63.339, I63.341, I63.342, I63.343, I63.349, I63.39, I63.40, I63.411, I63.412, I63.413, I63.419, I63.421, I63.422, I63.423, I63.429, I63.431, I63.432, I63.433, I63.439, I63.441, I63.442, I63.443, I63.449, I63.49, I63.50, I63.511, I63.512, I63.513, I63.519, I63.521, I63.522, I63.523, I63.529, I63.531, I63.532, I63.533, I63.539, I63.541, I63.542, I63.543, I63.549, I63.59, I63.6, I63.8, I63.9, I66.01, I66.02, I66.03, I66.09, I66.11, I66.12, I66.13, I66.19, I66.21, I66.22, I66.23, I66.29, I66.3, I66.8, I66.9, I67.841, I67.848, I67.89, I97.810, I97.811, I97.820, I97.821 |  |
|  | Hemorrhagic stroke | 430, 431 | I60.00, I60.01, I60.02, I60.10, I60.11, I60.12, I60.20, I60.21, I60.22, I60.30, I60.31, I60.32, I60.4, I60.50, I60.51, I60.52, I60.6, I60.7, I60.8, I60.9, I61.0, I61.1, I61.2, I61.3, I61.4, I61.5, I61.6, I61.8, I61.9 |  |
| ICD-9-CM: International Classification of Diseases, Ninth Revision, Clinical Modification; ICD-10-CM: International Classification of Diseases, Tenth Revision, Clinical Modification; TIA: transient ischemic attack | | | | |
