# Supplementary material for: Impact of empiric potassium supplementation on mortality, sudden cardiac arrest and stroke in furosemide initiators
Source: Br J Clin Pharmacol. 2026 May 3;92(8):2924–36. doi: 10.1002/bcp.70584 (PMC13421057; doi:10.1002/bcp.70584)
Supplement: Supplementary file 11 — Table S4. Covariates empirically identified by the high‐dimensional propensity score method for the <40 mg/day furosemide cohort. [file BCP-92-2924-s008.docx]

**Table S4. Covariates empirically identified by the high-dimensional propensity score method for the <40 mg/day furosemide cohort**

| **Data Dimension** | **Variable** | **Code** | **Code description** | **Z-bias = 5** |
| --- | --- | --- | --- | --- |
| Drug | D01V000Once | Drug-specific NDCs | acetaminophen |  |
| Drug | D01V000Spor | Drug-specific NDCs | acetaminophen |  |
| Drug | D01V158Once | Drug-specific NDCs | hydrocodone bitartrate |  |
| Drug | D01V158Spor | Drug-specific NDCs | hydrocodone bitartrate |  |
| Drug | D01V212Once | Drug-specific NDCs | levofloxacin |  |
| Drug | D01V524Once | Drug-specific NDCs | clopidogrel bisulfate |  |
| Drug | D01V824Once | Drug-specific NDCs | tiotropium |  |
| Inpatient ICD-9-CM diagnosis | D02V000Freq | 433 | precerebral occlusion |  |
| Inpatient ICD-9-CM diagnosis | D02V000Once | 433 | precerebral occlusion |  |
| Inpatient ICD-9-CM diagnosis | D02V001Freq | 496 | chronic airway obstruction, not elsewhere classified |  |
| Inpatient ICD-9-CM diagnosis | D02V001Once | 496 | chronic airway obstruction, not elsewhere classified |  |
| Inpatient ICD-9-CM diagnosis | D02V001Spor | 496 | chronic airway obstruction, not elsewhere classified |  |
| Inpatient ICD-9-CM diagnosis | D02V002Freq | 780 | general symptoms |  |
| Inpatient ICD-9-CM diagnosis | D02V002Once | 780 | general symptoms |  |
| Inpatient ICD-9-CM diagnosis | D02V002Spor | 780 | general symptoms |  |
| Inpatient ICD-9-CM diagnosis | D02V003Freq | 786 | symptoms involving respiratory system and other chest symptoms |  |
| Inpatient ICD-9-CM diagnosis | D02V003Once | 786 | symptoms involving respiratory system and other chest symptoms |  |
| Inpatient ICD-9-CM diagnosis | D02V003Spor | 786 | symptoms involving respiratory system and other chest symptoms |  |
| Inpatient ICD-9-CM diagnosis | D02V004Once | V12 | personal history of certain other diseases |  |
| Inpatient ICD-9-CM diagnosis | D02V005Freq | 250 | diabetes mellitus |  |
| Inpatient ICD-9-CM diagnosis | D02V005Once | 250 | diabetes mellitus |  |
| Inpatient ICD-9-CM diagnosis | D02V005Spor | 250 | diabetes mellitus |  |
| Inpatient ICD-9-CM diagnosis | D02V006Freq | 401 | essential hypertension |  |
| Inpatient ICD-9-CM diagnosis | D02V006Once | 401 | essential hypertension |  |
| Inpatient ICD-9-CM diagnosis | D02V006Spor | 401 | essential hypertension |  |
| Inpatient ICD-9-CM diagnosis | D02V009Once | 402 | hypertensive heart disease |  |
| Inpatient ICD-9-CM diagnosis | D02V011Freq | 424 | other diseases of endocardium |  |
| Inpatient ICD-9-CM diagnosis | D02V011Once | 424 | other diseases of endocardium |  |
| Inpatient ICD-9-CM diagnosis | D02V011Spor | 424 | other diseases of endocardium |  |
| Inpatient ICD-9-CM diagnosis | D02V012Freq | 427 | cardiac dysrhythmias |  |
| Inpatient ICD-9-CM diagnosis | D02V012Once | 427 | cardiac dysrhythmias |  |
| Inpatient ICD-9-CM diagnosis | D02V012Spor | 427 | cardiac dysrhythmias |  |
| Inpatient ICD-9-CM diagnosis | D02V013Freq | 428 | heart failure |  |
| Inpatient ICD-9-CM diagnosis | D02V013Once | 428 | heart failure |  |
| Inpatient ICD-9-CM diagnosis | D02V013Spor | 428 | heart failure |  |
| Inpatient ICD-9-CM diagnosis | D02V014Once | 599 | other disorders of urethra and urinary tract |  |
| Inpatient ICD-9-CM diagnosis | D02V014Spor | 599 | other disorders of urethra and urinary tract |  |
| Inpatient ICD-9-CM diagnosis | D02V015Once | 790 | abnormal blood findings |  |
| Inpatient ICD-9-CM diagnosis | D02V015Spor | 790 | abnormal blood findings |  |
| Inpatient ICD-9-CM diagnosis | D02V016Once | V15 | other personal history presenting hazards to health |  |
| Inpatient ICD-9-CM diagnosis | D02V018Once | 244 | acquired hypothyroidism |  |
| Inpatient ICD-9-CM diagnosis | D02V022Freq | 486 | pneumonia |  |
| Inpatient ICD-9-CM diagnosis | D02V022Once | 486 | pneumonia |  |
| Inpatient ICD-9-CM diagnosis | D02V022Spor | 486 | pneumonia |  |
| Inpatient ICD-9-CM diagnosis | D02V023Once | 593 | other disorders of kidney and ureter |  |
| Inpatient ICD-9-CM diagnosis | D02V027Freq | 285 | other and unspecified anemias |  |
| Inpatient ICD-9-CM diagnosis | D02V027Once | 285 | other and unspecified anemias |  |
| Inpatient ICD-9-CM diagnosis | D02V027Spor | 285 | other and unspecified anemias |  |
| Inpatient ICD-9-CM diagnosis | D02V028Freq | 411 | other acute and subacute forms of ischemic heart disease |  |
| Inpatient ICD-9-CM diagnosis | D02V028Once | 411 | other acute and subacute forms of ischemic heart disease |  |
| Inpatient ICD-9-CM diagnosis | D02V028Spor | 411 | other acute and subacute forms of ischemic heart disease |  |
| Inpatient ICD-9-CM diagnosis | D02V029Freq | 425 | cardiomyopathy |  |
| Inpatient ICD-9-CM diagnosis | D02V029Once | 425 | cardiomyopathy |  |
| Inpatient ICD-9-CM diagnosis | D02V029Spor | 425 | cardiomyopathy |  |
| Inpatient ICD-9-CM diagnosis | D02V030Freq | 429 | ill-defined descriptions and complications of heart disease |  |
| Inpatient ICD-9-CM diagnosis | D02V030Once | 429 | ill-defined descriptions and complications of heart disease |  |
| Inpatient ICD-9-CM diagnosis | D02V031Once | 458 | hypotension |  |
| Inpatient ICD-9-CM diagnosis | D02V031Spor | 458 | hypotension |  |
| Inpatient ICD-9-CM diagnosis | D02V033Freq | V45 | other postprocedural states |  |
| Inpatient ICD-9-CM diagnosis | D02V033Once | V45 | other postprocedural states |  |
| Inpatient ICD-9-CM diagnosis | D02V034Once | 288 | diseases of white blood cells |  |
| Inpatient ICD-9-CM diagnosis | D02V034Spor | 288 | diseases of white blood cells |  |
| Inpatient ICD-9-CM diagnosis | D02V035Freq | 410 | acute myocardial infarction |  |
| Inpatient ICD-9-CM diagnosis | D02V035Once | 410 | acute myocardial infarction |  |
| Inpatient ICD-9-CM diagnosis | D02V035Spor | 410 | acute myocardial infarction |  |
| Inpatient ICD-9-CM diagnosis | D02V036Freq | 793 | nonspecific (abnormal) findings on radiological and other examination of body structure |  |
| Inpatient ICD-9-CM diagnosis | D02V036Once | 793 | nonspecific (abnormal) findings on radiological and other examination of body structure |  |
| Inpatient ICD-9-CM diagnosis | D02V038Once | 530 | diseases of esophagus |  |
| Inpatient ICD-9-CM diagnosis | D02V040Once | 493 | asthma |  |
| Inpatient ICD-9-CM diagnosis | D02V041Once | 715 | osteoarthrosis and allied disorders |  |
| Inpatient ICD-9-CM diagnosis | D02V042Once | 719 | other and unspecified disorders of joint |  |
| Inpatient ICD-9-CM diagnosis | D02V045Freq | 511 | pleurisy |  |
| Inpatient ICD-9-CM diagnosis | D02V045Once | 511 | pleurisy |  |
| Inpatient ICD-9-CM diagnosis | D02V045Spor | 511 | pleurisy |  |
| Inpatient ICD-9-CM diagnosis | D02V046Freq | 515 | postinflammatory pulmonary fibrosis |  |
| Inpatient ICD-9-CM diagnosis | D02V046Once | 515 | postinflammatory pulmonary fibrosis |  |
| Inpatient ICD-9-CM diagnosis | D02V048Once | 789 | other symptoms involving abdomen and pelvis |  |
| Inpatient ICD-9-CM diagnosis | D02V049Freq | 799 | other ill-defined and unknown causes of morbidity and mortality |  |
| Inpatient ICD-9-CM diagnosis | D02V049Once | 799 | other ill-defined and unknown causes of morbidity and mortality |  |
| Inpatient ICD-9-CM diagnosis | D02V049Spor | 799 | other ill-defined and unknown causes of morbidity and mortality |  |
| Inpatient ICD-9-CM diagnosis | D02V056Once | 423 | other diseases of pericardium |  |
| Inpatient ICD-9-CM diagnosis | D02V057Freq | 272 | disorders of lipoid metabolism |  |
| Inpatient ICD-9-CM diagnosis | D02V057Once | 272 | disorders of lipoid metabolism |  |
| Inpatient ICD-9-CM diagnosis | D02V058Freq | 414 | other forms of chronic ischemic heart disease |  |
| Inpatient ICD-9-CM diagnosis | D02V058Once | 414 | other forms of chronic ischemic heart disease |  |
| Inpatient ICD-9-CM diagnosis | D02V058Spor | 414 | other forms of chronic ischemic heart disease |  |
| Inpatient ICD-9-CM diagnosis | D02V059Freq | 518 | other lung diseases |  |
| Inpatient ICD-9-CM diagnosis | D02V059Once | 518 | other lung diseases |  |
| Inpatient ICD-9-CM diagnosis | D02V059Spor | 518 | other lung diseases |  |
| Inpatient ICD-9-CM diagnosis | D02V060Once | 584 | acute renal failure |  |
| Inpatient ICD-9-CM diagnosis | D02V060Spor | 584 | acute renal failure |  |
| Inpatient ICD-9-CM diagnosis | D02V064Freq | V58 | encounter for other and unspecified procedures and aftercare |  |
| Inpatient ICD-9-CM diagnosis | D02V064Once | V58 | encounter for other and unspecified procedures and aftercare |  |
| Inpatient ICD-9-CM diagnosis | D02V066Freq | 426 | conduction disorders |  |
| Inpatient ICD-9-CM diagnosis | D02V066Once | 426 | conduction disorders |  |
| Inpatient ICD-9-CM diagnosis | D02V067Once | 491 | chronic bronchitis |  |
| Inpatient ICD-9-CM diagnosis | D02V067Spor | 491 | chronic bronchitis |  |
| Inpatient ICD-9-CM diagnosis | D02V068Once | V46 | other machine dependence |  |
| Inpatient ICD-9-CM diagnosis | D02V070Freq | 276 | disorders of fluid electrolyte and acid-base balance |  |
| Inpatient ICD-9-CM diagnosis | D02V070Once | 276 | disorders of fluid electrolyte and acid-base balance |  |
| Inpatient ICD-9-CM diagnosis | D02V070Spor | 276 | disorders of fluid electrolyte and acid-base balance |  |
| Inpatient ICD-9-CM diagnosis | D02V073Once | 600 | hyperplasia of prostate |  |
| Inpatient ICD-9-CM diagnosis | D02V074Once | 787 | symptoms involving digestive system |  |
| Inpatient ICD-9-CM diagnosis | D02V074Spor | 787 | symptoms involving digestive system |  |
| Inpatient ICD-9-CM diagnosis | D02V076Once | 275 | disorders of mineral metabolism |  |
| Inpatient ICD-9-CM diagnosis | D02V077Freq | 441 | aortic aneurysm |  |
| Inpatient ICD-9-CM diagnosis | D02V077Once | 441 | aortic aneurysm |  |
| Inpatient ICD-9-CM diagnosis | D02V077Spor | 441 | aortic aneurysm |  |
| Inpatient ICD-9-CM diagnosis | D02V079Once | V10 | personal history of malignant neoplasm |  |
| Inpatient ICD-9-CM diagnosis | D02V080Once | 997 | complications affecting specified body system not elsewhere classified |  |
| Inpatient ICD-9-CM diagnosis | D02V081Once | 998 | other complications of procedures not elsewhere classified |  |
| Inpatient ICD-9-CM diagnosis | D02V083Freq | 794 | abnormal function study |  |
| Inpatient ICD-9-CM diagnosis | D02V083Once | 794 | abnormal function study |  |
| Inpatient ICD-9-CM diagnosis | D02V085Once | 729 | other disorders of soft tissues |  |
| Inpatient ICD-9-CM diagnosis | D02V094Freq | 514 | pulmonary congestion and hypostasis |  |
| Inpatient ICD-9-CM diagnosis | D02V094Once | 514 | pulmonary congestion and hypostasis |  |
| Inpatient ICD-9-CM diagnosis | D02V097Once | 443 | other peripheral vascular disease |  |
| Inpatient ICD-9-CM diagnosis | D02V102Once | 278 | overweight, obesity and other hyperalimentation |  |
| Inpatient ICD-9-CM diagnosis | D02V103Once | 305 | nondependent drug abuse |  |
| Inpatient ICD-9-CM diagnosis | D02V108Once | 038 | septicemia |  |
| Inpatient ICD-9-CM diagnosis | D02V110Once | 578 | gastrointestinal hemorrhage |  |
| Inpatient ICD-9-CM diagnosis | D02V113Once | 492 | emphysema |  |
| Inpatient ICD-9-CM diagnosis | D02V115Once | V43 | organ or tissue replaced by other means |  |
| Inpatient ICD-9-CM diagnosis | D02V117Freq | V72 | special examinations |  |
| Inpatient ICD-9-CM diagnosis | D02V117Once | V72 | special examinations |  |
| Inpatient ICD-9-CM diagnosis | D02V119Once | 397 | diseases of other endocardial structures |  |
| Inpatient ICD-9-CM diagnosis | D02V120Freq | 416 | chronic pulmonary heart disease |  |
| Inpatient ICD-9-CM diagnosis | D02V120Once | 416 | chronic pulmonary heart disease |  |
| Inpatient ICD-9-CM diagnosis | D02V124Once | 995 | certain adverse effects not elsewhere classified |  |
| Inpatient ICD-9-CM diagnosis | D02V124Spor | 995 | certain adverse effects not elsewhere classified |  |
| Inpatient ICD-9-CM diagnosis | D02V125Once | 996 | complications peculiar to certain specified procedures |  |
| Inpatient ICD-9-CM diagnosis | D02V131Freq | V67 | follow-up examination |  |
| Inpatient ICD-9-CM diagnosis | D02V131Once | V67 | follow-up examination |  |
| Inpatient ICD-9-CM diagnosis | D02V143Freq | 785 | symptoms involving cardiovascular system |  |
| Inpatient ICD-9-CM diagnosis | D02V143Once | 785 | symptoms involving cardiovascular system |  |
| Inpatient ICD-9-CM diagnosis | D02V143Spor | 785 | symptoms involving cardiovascular system |  |
| Inpatient ICD-9-CM diagnosis | D02V144Freq | 440 | atherosclerosis |  |
| Inpatient ICD-9-CM diagnosis | D02V144Once | 440 | atherosclerosis |  |
| Inpatient ICD-9-CM diagnosis | D02V147Freq | 413 | angina pectoris |  |
| Inpatient ICD-9-CM diagnosis | D02V147Once | 413 | angina pectoris |  |
| Inpatient ICD-9-CM diagnosis | D02V158Once | 784 | symptoms involving head and neck |  |
| Inpatient ICD-9-CM diagnosis | D02V171Once | 782 | symptoms involving skin and other integumentary tissue |  |
| Inpatient ICD-9-CM diagnosis | D02V229Once | 287 | purpura and other hemorrhagic conditions |  |
| Inpatient ICD-9-CM diagnosis | D02V240Freq | 412 | old myocardial infarction |  |
| Inpatient ICD-9-CM diagnosis | D02V240Once | 412 | old myocardial infarction |  |
| Inpatient ICD-9-CM diagnosis | D02V275Once | 280 | iron deficiency anemias |  |
| Inpatient ICD-9-CM diagnosis | D02V286Once | 396 | diseases of mitral and aortic valves |  |
| Inpatient ICD-9-CM diagnosis | D02V313Once | 507 | pneumonitis due to solids and liquids |  |
| Inpatient ICD-9-CM diagnosis | D02V345Once | 512 | pneumothorax |  |
| Inpatient ICD-9-CM diagnosis | D02V345Spor | 512 | pneumothorax |  |
| Inpatient ICD-10-CM diagnosis | D03V000Once | D62 | acute posthemorrhagic anemia |  |
| Inpatient ICD-10-CM diagnosis | D03V005Freq | I25 | chronic ischemic heart disease |  |
| Inpatient ICD-10-CM diagnosis | D03V005Once | I25 | chronic ischemic heart disease |  |
| Inpatient ICD-10-CM diagnosis | D03V005Spor | I25 | chronic ischemic heart disease |  |
| Inpatient ICD-10-CM diagnosis | D03V010Once | N18 | chronic kidney disease (ckd) |  |
| Inpatient ICD-10-CM diagnosis | D03V018Freq | Z95 | presence of cardiac and vascular implants and grafts |  |
| Inpatient ICD-10-CM diagnosis | D03V018Once | Z95 | presence of cardiac and vascular implants and grafts |  |
| Inpatient ICD-10-CM diagnosis | D03V018Spor | Z95 | presence of cardiac and vascular implants and grafts |  |
| Inpatient ICD-10-CM diagnosis | D03V020Once | J90 | pleural effusion, not elsewhere classified |  |
| Inpatient ICD-10-CM diagnosis | D03V042Freq | I50 | heart failure |  |
| Inpatient ICD-10-CM diagnosis | D03V042Spor | I50 | heart failure |  |
| Inpatient ICD-10-CM diagnosis | D03V060Once | N17 | acute kidney failure |  |
| Inpatient ICD-9-CM procedure | D04V000Freq | 37 | other operations on heart and pericardium |  |
| Inpatient ICD-9-CM procedure | D04V000Once | 37 | other operations on heart and pericardium |  |
| Inpatient ICD-9-CM procedure | D04V001Once | 88 | other diagnostic radiology and related techniques |  |
| Inpatient ICD-9-CM procedure | D04V001Spor | 88 | other diagnostic radiology and related techniques |  |
| Inpatient ICD-9-CM procedure | D04V004Freq | 99 | other nonoperative procedures |  |
| Inpatient ICD-9-CM procedure | D04V004Once | 99 | other nonoperative procedures |  |
| Inpatient ICD-9-CM procedure | D04V005Freq | 38 | incision, excision, and occlusion of vessels |  |
| Inpatient ICD-9-CM procedure | D04V005Once | 38 | incision, excision, and occlusion of vessels |  |
| Inpatient ICD-9-CM procedure | D04V007Once | 39 | other operations on vessels |  |
| Inpatient ICD-9-CM procedure | D04V014Once | 36 | operations on vessels of heart |  |
| Inpatient ICD-9-CM procedure | D04V014Spor | 36 | operations on vessels of heart |  |
| Inpatient ICD-9-CM procedure | D04V021Once | 96 | nonoperative intubation and irrigation |  |
| Inpatient ICD-9-CM procedure | D04V032Once | 89 | interview, evaluation, consultation, and examination |  |
| Inpatient ICD-10-CM procedure | D05V001Once | 02R | heart and great vessels replacement |  |
| Inpatient ICD-10-CM procedure | D05V009Once | 4A0 | measurement |  |
| Inpatient ICD-10-CM procedure | D05V010Once | B21 | imaging: heart fluoroscopy |  |
| Inpatient ICD-10-CM procedure | D05V010Spor | B21 | imaging: heart fluoroscopy |  |
| Inpatient ICD-10-CM procedure | D05V025Freq | 021 | bypass |  |
| Inpatient ICD-10-CM procedure | D05V025Once | 021 | bypass |  |
| Inpatient ICD-10-CM procedure | D05V025Spor | 021 | bypass |  |
| Inpatient ICD-10-CM procedure | D05V027Freq | 06B | excision |  |
| Inpatient ICD-10-CM procedure | D05V027Once | 06B | excision |  |
| Inpatient CPT-4/HCPCS procedure | D06V000Freq | 71010 | chest x-ray |  |
| Inpatient CPT-4/HCPCS procedure | D06V000Once | 71010 | chest x-ray |  |
| Inpatient CPT-4/HCPCS procedure | D06V000Spor | 71010 | chest x-ray |  |
| Inpatient CPT-4/HCPCS procedure | D06V001Once | 93880 | extracranial study |  |
| Inpatient CPT-4/HCPCS procedure | D06V002Freq | 99223 | initial hospital care |  |
| Inpatient CPT-4/HCPCS procedure | D06V002Once | 99223 | initial hospital care |  |
| Inpatient CPT-4/HCPCS procedure | D06V003Freq | 93010 | ecg report |  |
| Inpatient CPT-4/HCPCS procedure | D06V003Once | 93010 | ecg report |  |
| Inpatient CPT-4/HCPCS procedure | D06V004Freq | 99232 | subsequent hospital care |  |
| Inpatient CPT-4/HCPCS procedure | D06V004Once | 99232 | subsequent hospital care |  |
| Inpatient CPT-4/HCPCS procedure | D06V004Spor | 99232 | subsequent hospital care |  |
| Inpatient CPT-4/HCPCS procedure | D06V005Once | 78452 | myocardial perfusion imaging, tomographic |  |
| Inpatient CPT-4/HCPCS procedure | D06V006Once | 93016 | cardiovascular stress test |  |
| Inpatient CPT-4/HCPCS procedure | D06V007Once | 93018 | cardiovascular stress test |  |
| Inpatient CPT-4/HCPCS procedure | D06V008Once | 93306 | transthoracic echocardiography |  |
| Inpatient CPT-4/HCPCS procedure | D06V009Once | 99239 | hospital discharge day |  |
| Inpatient CPT-4/HCPCS procedure | D06V015Once | 93312 | echocardiography, transesophageal |  |
| Inpatient CPT-4/HCPCS procedure | D06V017Once | 93320 | doppler echo exam heart |  |
| Inpatient CPT-4/HCPCS procedure | D06V018Once | 93325 | doppler color flow add-on |  |
| Inpatient CPT-4/HCPCS procedure | D06V019Once | 93503 | insert/place heart catheter |  |
| Inpatient CPT-4/HCPCS procedure | D06V023Freq | 99291 | critical care, first hour |  |
| Inpatient CPT-4/HCPCS procedure | D06V023Once | 99291 | critical care, first hour |  |
| Inpatient CPT-4/HCPCS procedure | D06V023Spor | 99291 | critical care, first hour |  |
| Inpatient CPT-4/HCPCS procedure | D06V029Once | 99238 | hospital discharge day |  |
| Inpatient CPT-4/HCPCS procedure | D06V035Once | 99222 | initial hospital care |  |
| Inpatient CPT-4/HCPCS procedure | D06V036Freq | 99233 | subsequent hospital care |  |
| Inpatient CPT-4/HCPCS procedure | D06V036Once | 99233 | subsequent hospital care |  |
| Inpatient CPT-4/HCPCS procedure | D06V036Spor | 99233 | subsequent hospital care |  |
| Inpatient CPT-4/HCPCS procedure | D06V054Freq | 99231 | subsequent hospital care |  |
| Inpatient CPT-4/HCPCS procedure | D06V054Once | 99231 | subsequent hospital care |  |
| Inpatient CPT-4/HCPCS procedure | D06V054Spor | 99231 | subsequent hospital care |  |
| Inpatient CPT-4/HCPCS procedure | D06V057Once | 99292 | critical care, additional 30 minutes |  |
| Inpatient CPT-4/HCPCS procedure | D06V067Once | 70450 | ct head/brain without dye |  |
| Inpatient CPT-4/HCPCS procedure | D06V068Once | 74000 | x-ray abdomen |  |
| Inpatient CPT-4/HCPCS procedure | D06V073Once | 93510 | left heart catheterization |  |
| Inpatient CPT-4/HCPCS procedure | D06V074Once | 93543 | inject for heart x-rays |  |
| Inpatient CPT-4/HCPCS procedure | D06V076Once | 93545 | inject for coronary x-rays |  |
| Inpatient CPT-4/HCPCS procedure | D06V077Once | 93555 | imaging, cardiac catheterization |  |
| Inpatient CPT-4/HCPCS procedure | D06V078Once | 93556 | imaging, cardiac catheterization |  |
| Inpatient CPT-4/HCPCS procedure | D06V079Once | 99255 | inpatient consultation |  |
| Inpatient CPT-4/HCPCS procedure | D06V080Freq | 71020 | chest x-ray |  |
| Inpatient CPT-4/HCPCS procedure | D06V080Once | 71020 | chest x-ray |  |
| Inpatient CPT-4/HCPCS procedure | D06V081Once | 93458 | catheter placement in coronary artery(s) for coronary angiography |  |
| Inpatient CPT-4/HCPCS procedure | D06V089Once | 93308 | echocardiography, transthoracic, real-time with image documentation (2D) |  |
| Inpatient CPT-4/HCPCS procedure | D06V090Once | 93321 | doppler echocardiography |  |
| Inpatient CPT-4/HCPCS procedure | D06V091Once | 71275 | ct angiography, chest |  |
| Inpatient CPT-4/HCPCS procedure | D06V096Once | 99254 | inpatient consultation |  |
| Inpatient CPT-4/HCPCS procedure | D06V120Once | 36620 | insert catheter, artery |  |
| Inpatient CPT-4/HCPCS procedure | D06V1236Once | 33967 | placement of a percutaneous intra-aortic balloon pump |  |
| Inpatient CPT-4/HCPCS procedure | D06V126Once | 93970 | extremity study |  |
| Inpatient CPT-4/HCPCS procedure | D06V141Once | 93307 | echocardiography, transthoracic, real-time with image documentation (2D) |  |
| Inpatient CPT-4/HCPCS procedure | D06V152Once | 78478 | heart wall motion add-on |  |
| Inpatient CPT-4/HCPCS procedure | D06V153Once | 78480 | heart function add-on |  |
| Inpatient CPT-4/HCPCS procedure | D06V159Once | 76937 | ultrasound guidance for vascular access |  |
| Inpatient CPT-4/HCPCS procedure | D06V167Once | 88305 | tissue exam by pathologist |  |
| Inpatient CPT-4/HCPCS procedure | D06V183Once | 92980 | insert intracoronary stent |  |
| Inpatient CPT-4/HCPCS procedure | D06V208Once | 36556 | insertion of non-tunneled centrally inserted central venous catheter |  |
| Inpatient CPT-4/HCPCS procedure | D06V239Once | 33508 | endoscopic vein harvest |  |
| Inpatient CPT-4/HCPCS procedure | D06V241Once | 71250 | ct thorax without dye |  |
| Inpatient CPT-4/HCPCS procedure | D06V244Once | 94060 | evaluate wheezing |  |
| Inpatient CPT-4/HCPCS procedure | D06V313Once | 00567 | anesthesia for intrathoracic procedures |  |
| Outpatient ICD-9-CM diagnosis | D07V000Freq | 250 | diabetes mellitus |  |
| Outpatient ICD-9-CM diagnosis | D07V000Spor | 250 | diabetes mellitus |  |
| Outpatient ICD-9-CM diagnosis | D07V001Freq | 585 | chronic renal failure |  |
| Outpatient ICD-9-CM diagnosis | D07V001Once | 585 | chronic renal failure |  |
| Outpatient ICD-9-CM diagnosis | D07V001Spor | 585 | chronic renal failure |  |
| Outpatient ICD-9-CM diagnosis | D07V002Once | 791 | abnormal urine findings |  |
| Outpatient ICD-9-CM diagnosis | D07V003Freq | 272 | disorders of lipoid metabolism |  |
| Outpatient ICD-9-CM diagnosis | D07V003Once | 272 | disorders of lipoid metabolism |  |
| Outpatient ICD-9-CM diagnosis | D07V003Spor | 272 | disorders of lipoid metabolism |  |
| Outpatient ICD-9-CM diagnosis | D07V005Once | 722 | intervertebral disc disorders |  |
| Outpatient ICD-9-CM diagnosis | D07V008Freq | 786 | symptoms involving respiratory system and other chest symptoms |  |
| Outpatient ICD-9-CM diagnosis | D07V008Once | 786 | symptoms involving respiratory system and other chest symptoms |  |
| Outpatient ICD-9-CM diagnosis | D07V008Spor | 786 | symptoms involving respiratory system and other chest symptoms |  |
| Outpatient ICD-9-CM diagnosis | D07V009Freq | 401 | essential hypertension |  |
| Outpatient ICD-9-CM diagnosis | D07V009Once | 401 | essential hypertension |  |
| Outpatient ICD-9-CM diagnosis | D07V009Spor | 401 | essential hypertension |  |
| Outpatient ICD-9-CM diagnosis | D07V010Once | 410 | acute myocardial infarction |  |
| Outpatient ICD-9-CM diagnosis | D07V011Freq | 411 | other acute and subacute forms of ischemic heart disease |  |
| Outpatient ICD-9-CM diagnosis | D07V011Once | 411 | other acute and subacute forms of ischemic heart disease |  |
| Outpatient ICD-9-CM diagnosis | D07V012Freq | V45 | other postprocedural states |  |
| Outpatient ICD-9-CM diagnosis | D07V012Once | V45 | other postprocedural states |  |
| Outpatient ICD-9-CM diagnosis | D07V012Spor | V45 | other postprocedural states |  |
| Outpatient ICD-9-CM diagnosis | D07V014Freq | 794 | abnormal function study |  |
| Outpatient ICD-9-CM diagnosis | D07V014Once | 794 | abnormal function study |  |
| Outpatient ICD-9-CM diagnosis | D07V015Freq | 414 | other forms of chronic ischemic heart disease |  |
| Outpatient ICD-9-CM diagnosis | D07V015Once | 414 | other forms of chronic ischemic heart disease |  |
| Outpatient ICD-9-CM diagnosis | D07V015Spor | 414 | other forms of chronic ischemic heart disease |  |
| Outpatient ICD-9-CM diagnosis | D07V016Once | V04 | need for prophylactic vaccination and inoculation against certain viral diseases |  |
| Outpatient ICD-9-CM diagnosis | D07V017Freq | 496 | chronic airway obstruction, not elsewhere classified |  |
| Outpatient ICD-9-CM diagnosis | D07V017Once | 496 | chronic airway obstruction, not elsewhere classified |  |
| Outpatient ICD-9-CM diagnosis | D07V017Spor | 496 | chronic airway obstruction, not elsewhere classified |  |
| Outpatient ICD-9-CM diagnosis | D07V018Once | 491 | chronic bronchitis |  |
| Outpatient ICD-9-CM diagnosis | D07V018Spor | 491 | chronic bronchitis |  |
| Outpatient ICD-9-CM diagnosis | D07V019Freq | 518 | other lung diseases |  |
| Outpatient ICD-9-CM diagnosis | D07V019Once | 518 | other lung diseases |  |
| Outpatient ICD-9-CM diagnosis | D07V020Once | 790 | abnormal blood findings |  |
| Outpatient ICD-9-CM diagnosis | D07V021Freq | 428 | heart failure |  |
| Outpatient ICD-9-CM diagnosis | D07V021Once | 428 | heart failure |  |
| Outpatient ICD-9-CM diagnosis | D07V021Spor | 428 | heart failure |  |
| Outpatient ICD-9-CM diagnosis | D07V022Freq | 780 | general symptoms |  |
| Outpatient ICD-9-CM diagnosis | D07V022Once | 780 | general symptoms |  |
| Outpatient ICD-9-CM diagnosis | D07V022Spor | 780 | general symptoms |  |
| Outpatient ICD-9-CM diagnosis | D07V024Once | 367 | disorders of refraction |  |
| Outpatient ICD-9-CM diagnosis | D07V025Once | 733 | other disorders of bone and cartilage | Y |
| Outpatient ICD-9-CM diagnosis | D07V027Once | 366 | cataract |  |
| Outpatient ICD-9-CM diagnosis | D07V029Once | 416 | chronic pulmonary heart disease |  |
| Outpatient ICD-9-CM diagnosis | D07V030Once | 362 | other retinal disorders |  |
| Outpatient ICD-9-CM diagnosis | D07V032Once | 793 | nonspecific (abnormal) findings on radiological and other examination of body structure |  |
| Outpatient ICD-9-CM diagnosis | D07V033Once | 724 | other and unspecified disorders of back | Y |
| Outpatient ICD-9-CM diagnosis | D07V035Once | 466 | acute bronchitis and bronchiolitis |  |
| Outpatient ICD-9-CM diagnosis | D07V045Once | 599 | other disorders of urethra and urinary tract | Y |
| Outpatient ICD-9-CM diagnosis | D07V045Spor | 599 | other disorders of urethra and urinary tract |  |
| Outpatient ICD-9-CM diagnosis | D07V048Freq | 403 | hypertensive chronic kidney disease |  |
| Outpatient ICD-9-CM diagnosis | D07V048Once | 403 | hypertensive chronic kidney disease |  |
| Outpatient ICD-9-CM diagnosis | D07V048Spor | 403 | hypertensive chronic kidney disease |  |
| Outpatient ICD-9-CM diagnosis | D07V049Freq | V58 | encounter for other and unspecified procedures and aftercare |  |
| Outpatient ICD-9-CM diagnosis | D07V049Once | V58 | encounter for other and unspecified procedures and aftercare |  |
| Outpatient ICD-9-CM diagnosis | D07V049Spor | V58 | encounter for other and unspecified procedures and aftercare |  |
| Outpatient ICD-9-CM diagnosis | D07V055Once | V10 | personal history of malignant neoplasm |  |
| Outpatient ICD-9-CM diagnosis | D07V059Once | 729 | other disorders of soft tissues | Y |
| Outpatient ICD-9-CM diagnosis | D07V060Once | 788 | urinary system symptoms |  |
| Outpatient ICD-9-CM diagnosis | D07V067Once | 719 | other and unspecified disorders of joint | Y |
| Outpatient ICD-9-CM diagnosis | D07V068Once | 782 | symptoms involving skin and other integumentary tissue | Y |
| Outpatient ICD-9-CM diagnosis | D07V070Freq | 424 | other diseases of endocardium |  |
| Outpatient ICD-9-CM diagnosis | D07V070Once | 424 | other diseases of endocardium |  |
| Outpatient ICD-9-CM diagnosis | D07V070Spor | 424 | other diseases of endocardium |  |
| Outpatient ICD-9-CM diagnosis | D07V071Freq | 427 | cardiac dysrhythmias |  |
| Outpatient ICD-9-CM diagnosis | D07V071Once | 427 | cardiac dysrhythmias |  |
| Outpatient ICD-9-CM diagnosis | D07V071Spor | 427 | cardiac dysrhythmias |  |
| Outpatient ICD-9-CM diagnosis | D07V072Freq | 785 | symptoms involving cardiovascular system |  |
| Outpatient ICD-9-CM diagnosis | D07V072Once | 785 | symptoms involving cardiovascular system |  |
| Outpatient ICD-9-CM diagnosis | D07V073Once | V12 | personal history of certain other diseases |  |
| Outpatient ICD-9-CM diagnosis | D07V082Once | 238 | neoplasm of uncertain behavior of other and unspecified sites and tissues |  |
| Outpatient ICD-9-CM diagnosis | D07V083Once | 244 | acquired hypothyroidism | Y |
| Outpatient ICD-9-CM diagnosis | D07V085Once | 789 | other symptoms involving abdomen and pelvis | Y |
| Outpatient ICD-9-CM diagnosis | D07V089Once | 443 | other peripheral vascular disease |  |
| Outpatient ICD-9-CM diagnosis | D07V089Spor | 443 | other peripheral vascular disease |  |
| Outpatient ICD-9-CM diagnosis | D07V093Once | 600 | hyperplasia of prostate |  |
| Outpatient ICD-9-CM diagnosis | D07V093Spor | 600 | hyperplasia of prostate |  |
| Outpatient ICD-9-CM diagnosis | D07V096Once | 702 | other dermatoses |  |
| Outpatient ICD-9-CM diagnosis | D07V096Spor | 702 | other dermatoses |  |
| Outpatient ICD-9-CM diagnosis | D07V097Once | 715 | osteoarthrosis and allied disorders | Y |
| Outpatient ICD-9-CM diagnosis | D07V097Spor | 715 | osteoarthrosis and allied disorders | Y |
| Outpatient ICD-9-CM diagnosis | D07V098Once | 781 | symptoms involving nervous and musculoskeletal systems |  |
| Outpatient ICD-9-CM diagnosis | D07V108Freq | 426 | conduction disorders |  |
| Outpatient ICD-9-CM diagnosis | D07V108Once | 426 | conduction disorders |  |
| Outpatient ICD-9-CM diagnosis | D07V111Once | V43 | organ or tissue replaced by other means |  |
| Outpatient ICD-9-CM diagnosis | D07V114Once | 173 | other and unspecified malignant neoplasm of skin |  |
| Outpatient ICD-9-CM diagnosis | D07V114Spor | 173 | other and unspecified malignant neoplasm of skin |  |
| Outpatient ICD-9-CM diagnosis | D07V115Freq | 429 | ill-defined descriptions and complications of heart disease |  |
| Outpatient ICD-9-CM diagnosis | D07V115Once | 429 | ill-defined descriptions and complications of heart disease |  |
| Outpatient ICD-9-CM diagnosis | D07V117Freq | 799 | other ill-defined and unknown causes of morbidity and mortality |  |
| Outpatient ICD-9-CM diagnosis | D07V117Once | 799 | other ill-defined and unknown causes of morbidity and mortality |  |
| Outpatient ICD-9-CM diagnosis | D07V129Once | 285 | other and unspecified anemias |  |
| Outpatient ICD-9-CM diagnosis | D07V131Once | 425 | cardiomyopathy |  |
| Outpatient ICD-9-CM diagnosis | D07V131Spor | 425 | cardiomyopathy |  |
| Outpatient ICD-9-CM diagnosis | D07V132Once | 458 | hypotension |  |
| Outpatient ICD-9-CM diagnosis | D07V141Freq | 433 | precerebral occlusion |  |
| Outpatient ICD-9-CM diagnosis | D07V141Once | 433 | precerebral occlusion |  |
| Outpatient ICD-9-CM diagnosis | D07V143Once | 787 | symptoms involving digestive system | Y |
| Outpatient ICD-9-CM diagnosis | D07V161Once | 305 | nondependent drug abuse |  |
| Outpatient ICD-9-CM diagnosis | D07V166Freq | 486 | pneumonia |  |
| Outpatient ICD-9-CM diagnosis | D07V166Once | 486 | pneumonia |  |
| Outpatient ICD-9-CM diagnosis | D07V170Freq | 511 | pleurisy |  |
| Outpatient ICD-9-CM diagnosis | D07V170Once | 511 | pleurisy |  |
| Outpatient ICD-9-CM diagnosis | D07V189Freq | 440 | atherosclerosis |  |
| Outpatient ICD-9-CM diagnosis | D07V189Once | 440 | atherosclerosis |  |
| Outpatient ICD-9-CM diagnosis | D07V192Freq | 413 | angina pectoris |  |
| Outpatient ICD-9-CM diagnosis | D07V192Once | 413 | angina pectoris |  |
| Outpatient ICD-9-CM diagnosis | D07V196Once | V15 | other personal history presenting hazards to health |  |
| Outpatient ICD-9-CM diagnosis | D07V199Once | 682 | other cellulitis/abscess |  |
| Outpatient ICD-9-CM diagnosis | D07V213Once | V53 | fitting and adjustment of other device |  |
| Outpatient ICD-9-CM diagnosis | D07V219Freq | 492 | emphysema |  |
| Outpatient ICD-9-CM diagnosis | D07V219Once | 492 | emphysema |  |
| Outpatient ICD-9-CM diagnosis | D07V228Once | 276 | disorders of fluid electrolyte and acid-base balance |  |
| Outpatient ICD-9-CM diagnosis | D07V230Freq | 441 | aortic aneurysm |  |
| Outpatient ICD-9-CM diagnosis | D07V230Once | 441 | aortic aneurysm |  |
| Outpatient ICD-9-CM diagnosis | D07V230Spor | 441 | aortic aneurysm |  |
| Outpatient ICD-9-CM diagnosis | D07V242Once | 185 | malignant neoplasm of the prostate |  |
| Outpatient ICD-9-CM diagnosis | D07V251Freq | 412 | old myocardial infarction |  |
| Outpatient ICD-9-CM diagnosis | D07V251Once | 412 | old myocardial infarction |  |
| Outpatient ICD-9-CM diagnosis | D07V252Once | 402 | hypertensive heart disease |  |
| Outpatient ICD-9-CM diagnosis | D07V288Once | 436 | acute, but ill-defined, cerebrovascular disease |  |
| Outpatient ICD-9-CM diagnosis | D07V289Once | 435 | transient cerebral ischemia |  |
| Outpatient ICD-10-CM diagnosis | D08V001Once | E11 | type 2 diabetes mellitus | Y |
| Outpatient ICD-10-CM diagnosis | D08V002Once | I10 | essential (primary) hypertension | Y |
| Outpatient ICD-10-CM diagnosis | D08V002Spor | I10 | essential (primary) hypertension | Y |
| Outpatient ICD-10-CM diagnosis | D08V004Freq | N18 | chronic kidney disease (ckd) |  |
| Outpatient ICD-10-CM diagnosis | D08V004Once | N18 | chronic kidney disease (ckd) | Y |
| Outpatient ICD-10-CM diagnosis | D08V004Spor | N18 | chronic kidney disease (ckd) | Y |
| Outpatient ICD-10-CM diagnosis | D08V005Once | Z23 | encounter for immunization | Y |
| Outpatient ICD-10-CM diagnosis | D08V010Freq | E78 | disorders of lipoprotein metabolism and other lipidemias | Y |
| Outpatient ICD-10-CM diagnosis | D08V010Once | E78 | disorders of lipoprotein metabolism and other lipidemias | Y |
| Outpatient ICD-10-CM diagnosis | D08V010Spor | E78 | disorders of lipoprotein metabolism and otherlipidemias | Y |
| Outpatient ICD-10-CM diagnosis | D08V011Once | F41 | other anxiety disorders |  |
| Outpatient ICD-10-CM diagnosis | D08V012Once | I12 | hypertensive chronic kidney disease |  |
| Outpatient ICD-10-CM diagnosis | D08V012Spor | I12 | hypertensive chronic kidney disease | Y |
| Outpatient ICD-10-CM diagnosis | D08V016Once | N17 | acute kidney failure |  |
| Outpatient ICD-10-CM diagnosis | D08V016Spor | N17 | acute kidney failure |  |
| Outpatient ICD-10-CM diagnosis | D08V017Freq | Z68 | body mass index [bmi] |  |
| Outpatient ICD-10-CM diagnosis | D08V017Once | Z68 | body mass index [bmi] |  |
| Outpatient ICD-10-CM diagnosis | D08V017Spor | Z68 | body mass index [bmi] |  |
| Outpatient ICD-10-CM diagnosis | D08V019Once | K21 | gastro-esophageal reflux disease |  |
| Outpatient ICD-10-CM diagnosis | D08V019Spor | K21 | gastro-esophageal reflux disease |  |
| Outpatient ICD-10-CM diagnosis | D08V021Once | J06 | acute upper respiratory infections of multiple and unspecified sites |  |
| Outpatient ICD-10-CM diagnosis | D08V023Freq | E66 | overweight and obesity |  |
| Outpatient ICD-10-CM diagnosis | D08V023Once | E66 | overweight and obesity |  |
| Outpatient ICD-10-CM diagnosis | D08V023Spor | E66 | overweight and obesity |  |
| Outpatient ICD-10-CM diagnosis | D08V028Once | M54 | dorsalgia |  |
| Outpatient ICD-10-CM diagnosis | D08V028Spor | M54 | dorsalgia |  |
| Outpatient ICD-10-CM diagnosis | D08V030Once | R60 | edema, not elsewhere classified |  |
| Outpatient ICD-10-CM diagnosis | D08V032Freq | E55 | vitamin d deficiency |  |
| Outpatient ICD-10-CM diagnosis | D08V032Once | E55 | vitamin d deficiency |  |
| Outpatient ICD-10-CM diagnosis | D08V033Freq | Z00 | encounter for general examination without complaint, suspected or reported diagnosis |  |
| Outpatient ICD-10-CM diagnosis | D08V033Once | Z00 | encounter for general examination without complaint, suspected or reported diagnosis |  |
| Outpatient ICD-10-CM diagnosis | D08V034Once | H52 | disorders of refraction and accommodation |  |
| Outpatient ICD-10-CM diagnosis | D08V040Freq | I50 | heart failure |  |
| Outpatient ICD-10-CM diagnosis | D08V040Once | I50 | heart failure |  |
| Outpatient ICD-10-CM diagnosis | D08V040Spor | I50 | heart failure |  |
| Outpatient ICD-10-CM diagnosis | D08V044Once | E03 | other hypothyroidism |  |
| Outpatient ICD-10-CM diagnosis | D08V044Spor | E03 | other hypothyroidism |  |
| Outpatient ICD-10-CM diagnosis | D08V049Freq | M79 | other and unspecified soft tissue disorders, not elsewhere classified |  |
| Outpatient ICD-10-CM diagnosis | D08V049Once | M79 | other and unspecified soft tissue disorders, not elsewhere classified |  |
| Outpatient ICD-10-CM diagnosis | D08V049Spor | M79 | other and unspecified soft tissue disorders, not elsewhere classified |  |
| Outpatient ICD-10-CM diagnosis | D08V070Once | N39 | other disorders of urinary system | Y |
| Outpatient ICD-10-CM diagnosis | D08V073Once | E87 | other disorders of fluid, electrolyte and acid-base balance |  |
| Outpatient ICD-10-CM diagnosis | D08V074Spor | I25 | chronic ischemic heart disease |  |
| Outpatient ICD-10-CM diagnosis | D08V078Freq | I42 | cardiomyopathy |  |
| Outpatient ICD-10-CM diagnosis | D08V078Once | I42 | cardiomyopathy |  |
| Outpatient ICD-10-CM diagnosis | D08V078Spor | I42 | cardiomyopathy |  |
| Outpatient ICD-10-CM diagnosis | D08V083Once | R73 | elevated blood glucose level |  |
| Outpatient ICD-10-CM diagnosis | D08V098Once | Z01 | encounter for other special examination without complaint, suspected or reported diagnosis |  |
| Outpatient ICD-10-CM diagnosis | D08V107Once | H25 | age-related cataract |  |
| Outpatient ICD-10-CM diagnosis | D08V110Once | H43 | disorders of vitreous body |  |
| Outpatient ICD-10-CM diagnosis | D08V122Once | Z13 | encounter for screening for other diseases anddisorders |  |
| Outpatient ICD-10-CM diagnosis | D08V124Freq | Z12 | encounter for screening for malignant neoplasms |  |
| Outpatient ICD-10-CM diagnosis | D08V124Once | Z12 | encounter for screening for malignant neoplasms |  |
| Outpatient ICD-10-CM diagnosis | D08V125Once | H04 | disorders of lacrimal system |  |
| Outpatient ICD-10-CM diagnosis | D08V142Freq | M25 | other joint disorder, not elsewhere classified |  |
| Outpatient ICD-10-CM diagnosis | D08V142Once | M25 | other joint disorder, not elsewhere classified |  |
| Outpatient ICD-10-CM diagnosis | D08V142Spor | M25 | other joint disorder, not elsewhere classified |  |
| Outpatient ICD-10-CM diagnosis | D08V151Once | J01 | acute sinusitis |  |
| Outpatient ICD-10-CM diagnosis | D08V157Once | R10 | abdominal and pelvic pain | Y |
| Outpatient ICD-10-CM diagnosis | D08V164Once | Z96 | presence of other functional implants |  |
| Outpatient ICD-10-CM diagnosis | D08V169Once | M19 | other and unspecified osteoarthritis |  |
| Outpatient ICD-10-CM diagnosis | D08V192Once | J30 | vasomotor and allergic rhinitis |  |
| Outpatient ICD-10-CM diagnosis | D08V199Once | M17 | osteoarthritis of knee |  |
| Outpatient ICD-10-CM diagnosis | D08V199Spor | M17 | osteoarthritis of knee |  |
| Outpatient ICD-10-CM diagnosis | D08V264Once | R35 | polyuria |  |
| Outpatient ICD-10-CM diagnosis | D08V386Once | J02 | acute pharyngitis |  |
| Outpatient ICD-10-CM diagnosis | D08V399Freq | D63 | anemia in chronic diseases classified elsewhere |  |
| Outpatient ICD-10-CM diagnosis | D08V399Once | D63 | anemia in chronic diseases classified elsewhere |  |
| Outpatient ICD-10-CM diagnosis | D08V399Spor | D63 | anemia in chronic diseases classified elsewhere |  |
| Outpatient ICD-10-CM diagnosis | D08V578Once | Z11 | encounter for screening for infectious and parasitic diseases |  |
| Outpatient CPT-4/HCPCS procedure | D11V004Freq | 36415 | routine venipuncture | Y |
| Outpatient CPT-4/HCPCS procedure | D11V004Spor | 36415 | routine venipuncture | Y |
| Outpatient CPT-4/HCPCS procedure | D11V008Once | 82043 | microalbumin, quantitative | Y |
| Outpatient CPT-4/HCPCS procedure | D11V009Once | 82570 | assay urine creatinine | Y |
| Outpatient CPT-4/HCPCS procedure | D11V011Freq | 83036 | glycosylated hemoglobin test | Y |
| Outpatient CPT-4/HCPCS procedure | D11V011Once | 83036 | glycosylated hemoglobin test | Y |
| Outpatient CPT-4/HCPCS procedure | D11V011Spor | 83036 | glycosylated hemoglobin test | Y |
| Outpatient CPT-4/HCPCS procedure | D11V013Once | 84100 | assay phosphorus | Y |
| Outpatient CPT-4/HCPCS procedure | D11V020Freq | 71010 | chest x-ray |  |
| Outpatient CPT-4/HCPCS procedure | D11V020Once | 71010 | chest x-ray |  |
| Outpatient CPT-4/HCPCS procedure | D11V021Freq | 93010 | ecg report |  |
| Outpatient CPT-4/HCPCS procedure | D11V021Once | 93010 | ecg report |  |
| Outpatient CPT-4/HCPCS procedure | D11V023Once | 99291 | critical care, first hour |  |
| Outpatient CPT-4/HCPCS procedure | D11V029Freq | 99285 | emergency department visit |  |
| Outpatient CPT-4/HCPCS procedure | D11V029Once | 99285 | emergency department visit |  |
| Outpatient CPT-4/HCPCS procedure | D11V030Once | 94060 | evaluate wheezing |  |
| Outpatient CPT-4/HCPCS procedure | D11V033Once | 94760 | measure blood oxygen level |  |
| Outpatient CPT-4/HCPCS procedure | D11V034Freq | 71020 | chest x-ray |  |
| Outpatient CPT-4/HCPCS procedure | D11V034Once | 71020 | chest x-ray |  |
| Outpatient CPT-4/HCPCS procedure | D11V036Once | A0425 | ground mileage, per statute mile |  |
| Outpatient CPT-4/HCPCS procedure | D11V037Once | A0427 | ambulance service, advanced life support, emergency transport, level 1 (ALS 1-emergency) |  |
| Outpatient CPT-4/HCPCS procedure | D11V052Freq | 80048 | basic metabolic panel (calcium total) | Y |
| Outpatient CPT-4/HCPCS procedure | D11V052Once | 80048 | basic metabolic panel (calcium total) | Y |
| Outpatient CPT-4/HCPCS procedure | D11V055Once | 80050 | general health panel |  |
| Outpatient CPT-4/HCPCS procedure | D11V056Once | 82306 | assay vitamin d | Y |
| Outpatient CPT-4/HCPCS procedure | D11V075Once | 88175 | cytopathology, cervical or vaginal (any reporting system), collected in preservative fluid, automated thin layer preparation |  |
| Outpatient CPT-4/HCPCS procedure | D11V091Once | 84153 | prostate specific antigen (PSA) |  |
| Outpatient CPT-4/HCPCS procedure | D11V093Once | 90658 | influenza virus vaccine, trivalent (IIV3), split virus, 0.5 mL dosage, for intramuscular use |  |
| Outpatient CPT-4/HCPCS procedure | D11V095Once | G0008 | administration of influenza virus vaccine | Y |
| Outpatient CPT-4/HCPCS procedure | D11V114Freq | 93000 | ecg complete |  |
| Outpatient CPT-4/HCPCS procedure | D11V114Once | 93000 | ecg complete |  |
| Outpatient CPT-4/HCPCS procedure | D11V115Once | 93306 | electrocardiogram, routine ecg with at least 12 leads |  |
| Outpatient CPT-4/HCPCS procedure | D11V145Once | G0463 | hospital outpatient clinic visit or assessment and management of a patient | Y |
| Outpatient CPT-4/HCPCS procedure | D11V145Spor | G0463 | hospital outpatient clinic visit or assessment and management of a patient |  |
| Outpatient CPT-4/HCPCS procedure | D11V147Freq | 85610 | prothrombin time |  |
| Outpatient CPT-4/HCPCS procedure | D11V147Once | 85610 | prothrombin time |  |
| Outpatient CPT-4/HCPCS procedure | D11V169Once | 93880 | extracranial study |  |
| Outpatient CPT-4/HCPCS procedure | D11V218Once | 83880 | natriuretic peptide |  |
| Outpatient CPT-4/HCPCS procedure | D11V219Once | 85730 | thromboplastin time, partial |  |
| Outpatient CPT-4/HCPCS procedure | D11V273Once | E1390 | oxygen concentrator, single delivery port, capable of delivering 85 percent or greater oxygen concentration at the prescribed flow rate |  |
| Outpatient CPT-4/HCPCS procedure | D11V273Spor | E1390 | oxygen concentrator, single delivery port, capable of delivering 85 percent or greater oxygen concentration at the prescribed flow rate |  |
| Outpatient CPT-4/HCPCS procedure | D11V347Once | 11721 | debride nail, 6 or more |  |
| Outpatient CPT-4/HCPCS procedure | D11V386Once | G0439 | annual wellness visit, subsequent |  |
| Outpatient CPT-4/HCPCS procedure | D11V429Once | 99396 | periodic comprehensive preventive medicine reevaluation and management of an individual |  |
| Outpatient CPT-4/HCPCS procedure | D11V509Once | 77067 | screening mammography, bilateral (2-view study of each breast) |  |
| Outpatient CPT-4/HCPCS procedure | D11V557Once | 93325 | doppler color flow add-on |  |
| Outpatient CPT-4/HCPCS procedure | D11V633Once | 93015 | cardiovascular stress test |  |
| Outpatient CPT-4/HCPCS procedure | D11V747Once | 71046 | radiologic examination, chest |  |
| Outpatient CPT-4/HCPCS procedure | D11V776Once | 90686 | influenza virus vaccine, quadrivalent |  |
| Other ICD-9-CM diagnosis | D12V004Once | V57 | rehabilitation procedure |  |
| Laboratory | D17V000Once | 62238-1 | glomerular filtration rate/1.73 sq m.predicted [volume  rate/area] in serum or plasma by creatinine-b |  |
| Laboratory | D17V089Freq | 88293-6 | glomerular filtration rate/1.73 sq m.predicted among blacks [volume rate/area] in serum, plasma or blood by creatinine-based formula (ckd-epi) |  |
| Laboratory | D17V089Once | 88293-6 | glomerular filtration rate/1.73 sq m.predicted among blacks [volume rate/area] in serum, plasma or blood by creatinine-based formula (ckd-epi) |  |
| Laboratory | D17V090Freq | 88294-4 | glomerular filtration rate/1.73 sq m.predicted among non-blacks [volume rate/area] in serum, plasma or blood by creatinine-based formula (ckd-epi) |  |
| Laboratory | D17V090Once | 88294-4 | glomerular filtration rate/1.73 sq m.predicted among non-blacks [volume rate/area] in serum, plasma or blood by creatinine-based formula (ckd-epi) |  |
| Laboratory | D17V110Once | 62292-8 | 25-hydroxyvitamin d2+25-hydroxyvitamin d3  [mass/volume] in serum or plasma |  |
| Laboratory | D17V149Freq | 11580-8 | thyrotropin [units/volume] in serum or plasma by  detection limit <= 0.005 miu/l |  |
| Laboratory | D17V149Once | 11580-8 | thyrotropin [units/volume] in serum or plasma by  detection limit <= 0.005 miu/l |  |
