# Supplementary material for: Impact of empiric potassium supplementation on mortality, sudden cardiac arrest and stroke in furosemide initiators
Source: Br J Clin Pharmacol. 2026 May 3;92(8):2924–36. doi: 10.1002/bcp.70584 (PMC13421057; doi:10.1002/bcp.70584)
Supplement: Supplementary file 12 — Table S5. Covariates empirically identified by the high‐dimensional propensity score method for the ≥40 mg/day furosemide cohort. [file BCP-92-2924-s014.docx]

**Table S5. Covariates empirically identified by the high-dimensional propensity score method for the ≥40 mg/day furosemide cohort**

| **Data Dimension** | **Variable** | **Code** | **Code description** | **Z-bias = 5** |
| --- | --- | --- | --- | --- |
| Drug | D01V024Once | Drug-specific NDCs | calcitriol |  |
| Drug | D01V024Spor | Drug-specific NDCs | calcitriol |  |
| Drug | D01V500Once | Drug-specific NDCs | clopidogrel bisulfate |  |
| Inpatient ICD-9-CM diagnosis | D02V000Once | 244 | acquired hypothyroidism |  |
| Inpatient ICD-9-CM diagnosis | D02V001Freq | 272 | disorders of lipoid metabolism |  |
| Inpatient ICD-9-CM diagnosis | D02V001Once | 272 | disorders of lipoid metabolism |  |
| Inpatient ICD-9-CM diagnosis | D02V001Spor | 272 | disorders of lipoid metabolism |  |
| Inpatient ICD-9-CM diagnosis | D02V002Freq | 401 | essential hypertension |  |
| Inpatient ICD-9-CM diagnosis | D02V002Once | 401 | essential hypertension |  |
| Inpatient ICD-9-CM diagnosis | D02V002Spor | 401 | essential hypertension |  |
| Inpatient ICD-9-CM diagnosis | D02V003Freq | 414 | other forms of chronic ischemic heart disease |  |
| Inpatient ICD-9-CM diagnosis | D02V003Once | 414 | other forms of chronic ischemic heart disease |  |
| Inpatient ICD-9-CM diagnosis | D02V003Spor | 414 | other forms of chronic ischemic heart disease |  |
| Inpatient ICD-9-CM diagnosis | D02V004Freq | 424 | other diseases of endocardium |  |
| Inpatient ICD-9-CM diagnosis | D02V004Once | 424 | other diseases of endocardium |  |
| Inpatient ICD-9-CM diagnosis | D02V004Spor | 424 | other diseases of endocardium |  |
| Inpatient ICD-9-CM diagnosis | D02V005Freq | 425 | cardiomyopathy |  |
| Inpatient ICD-9-CM diagnosis | D02V005Once | 425 | cardiomyopathy |  |
| Inpatient ICD-9-CM diagnosis | D02V005Spor | 425 | cardiomyopathy |  |
| Inpatient ICD-9-CM diagnosis | D02V006Freq | 427 | cardiac dysrhythmias |  |
| Inpatient ICD-9-CM diagnosis | D02V006Once | 427 | cardiac dysrhythmias |  |
| Inpatient ICD-9-CM diagnosis | D02V006Spor | 427 | cardiac dysrhythmias |  |
| Inpatient ICD-9-CM diagnosis | D02V007Once | 600 | hyperplasia of prostate |  |
| Inpatient ICD-9-CM diagnosis | D02V008Freq | V58 | encounter for other and unspecified procedures and aftercare |  |
| Inpatient ICD-9-CM diagnosis | D02V008Once | V58 | encounter for other and unspecified procedures and aftercare |  |
| Inpatient ICD-9-CM diagnosis | D02V008Spor | V58 | encounter for other and unspecified procedures and aftercare |  |
| Inpatient ICD-9-CM diagnosis | D02V009Once | V43 | organ or tissue replaced by other means |  |
| Inpatient ICD-9-CM diagnosis | D02V011Freq | 276 | disorders of fluid electrolyte and acid-base balance |  |
| Inpatient ICD-9-CM diagnosis | D02V011Once | 276 | disorders of fluid electrolyte and acid-base balance |  |
| Inpatient ICD-9-CM diagnosis | D02V011Spor | 276 | disorders of fluid electrolyte and acid-base balance |  |
| Inpatient ICD-9-CM diagnosis | D02V012Freq | 410 | acute myocardial infarction |  |
| Inpatient ICD-9-CM diagnosis | D02V012Once | 410 | acute myocardial infarction |  |
| Inpatient ICD-9-CM diagnosis | D02V012Spor | 410 | acute myocardial infarction |  |
| Inpatient ICD-9-CM diagnosis | D02V013Freq | 428 | heart failure |  |
| Inpatient ICD-9-CM diagnosis | D02V013Once | 428 | heart failure |  |
| Inpatient ICD-9-CM diagnosis | D02V013Spor | 428 | heart failure |  |
| Inpatient ICD-9-CM diagnosis | D02V017Freq | 518 | other lung diseases |  |
| Inpatient ICD-9-CM diagnosis | D02V017Once | 518 | other lung diseases |  |
| Inpatient ICD-9-CM diagnosis | D02V017Spor | 518 | other lung diseases |  |
| Inpatient ICD-9-CM diagnosis | D02V021Freq | 785 | symptoms involving cardiovascular system |  |
| Inpatient ICD-9-CM diagnosis | D02V021Once | 785 | symptoms involving cardiovascular system |  |
| Inpatient ICD-9-CM diagnosis | D02V021Spor | 785 | symptoms involving cardiovascular system |  |
| Inpatient ICD-9-CM diagnosis | D02V023Once | 458 | hypotension |  |
| Inpatient ICD-9-CM diagnosis | D02V023Spor | 458 | hypotension |  |
| Inpatient ICD-9-CM diagnosis | D02V024Once | 530 | diseases of esophagus |  |
| Inpatient ICD-9-CM diagnosis | D02V025Spor | 584 | acute renal failure |  |
| Inpatient ICD-9-CM diagnosis | D02V028Freq | 429 | ill-defined descriptions and complications of heart disease |  |
| Inpatient ICD-9-CM diagnosis | D02V028Once | 429 | ill-defined descriptions and complications of heart disease |  |
| Inpatient ICD-9-CM diagnosis | D02V029Freq | 786 | symptoms involving respiratory system and other chest symptoms |  |
| Inpatient ICD-9-CM diagnosis | D02V029Once | 786 | symptoms involving respiratory system and other chest symptoms |  |
| Inpatient ICD-9-CM diagnosis | D02V029Spor | 786 | symptoms involving respiratory system and other chest symptoms |  |
| Inpatient ICD-9-CM diagnosis | D02V030Freq | 585 | chronic renal failure |  |
| Inpatient ICD-9-CM diagnosis | D02V030Once | 585 | chronic renal failure |  |
| Inpatient ICD-9-CM diagnosis | D02V030Spor | 585 | chronic renal failure |  |
| Inpatient ICD-9-CM diagnosis | D02V031Freq | 496 | chronic airway obstruction, not elsewhere classified |  |
| Inpatient ICD-9-CM diagnosis | D02V031Once | 496 | chronic airway obstruction, not elsewhere classified |  |
| Inpatient ICD-9-CM diagnosis | D02V031Spor | 496 | chronic airway obstruction, not elsewhere classified |  |
| Inpatient ICD-9-CM diagnosis | D02V032Freq | 416 | chronic pulmonary heart disease |  |
| Inpatient ICD-9-CM diagnosis | D02V032Once | 416 | chronic pulmonary heart disease |  |
| Inpatient ICD-9-CM diagnosis | D02V033Freq | 799 | other ill-defined and unknown causes of morbidity and mortality |  |
| Inpatient ICD-9-CM diagnosis | D02V033Once | 799 | other ill-defined and unknown causes of morbidity and mortality |  |
| Inpatient ICD-9-CM diagnosis | D02V033Spor | 799 | other ill-defined and unknown causes of morbidity and mortality |  |
| Inpatient ICD-9-CM diagnosis | D02V034Once | 486 | pneumonia |  |
| Inpatient ICD-9-CM diagnosis | D02V034Spor | 486 | pneumonia |  |
| Inpatient ICD-9-CM diagnosis | D02V039Freq | 511 | pleurisy |  |
| Inpatient ICD-9-CM diagnosis | D02V039Once | 511 | pleurisy |  |
| Inpatient ICD-9-CM diagnosis | D02V039Spor | 511 | pleurisy |  |
| Inpatient ICD-9-CM diagnosis | D02V040Once | 729 | other disorders of soft tissues |  |
| Inpatient ICD-9-CM diagnosis | D02V041Once | 787 | symptoms involving digestive system |  |
| Inpatient ICD-9-CM diagnosis | D02V045Once | 287 | purpura and other hemorrhagic conditions |  |
| Inpatient ICD-9-CM diagnosis | D02V046Freq | 411 | ischemic heart disease |  |
| Inpatient ICD-9-CM diagnosis | D02V046Once | 411 | ischemic heart disease |  |
| Inpatient ICD-9-CM diagnosis | D02V046Spor | 411 | ischemic heart disease |  |
| Inpatient ICD-9-CM diagnosis | D02V047Freq | 794 | abnormal function study |  |
| Inpatient ICD-9-CM diagnosis | D02V047Once | 794 | abnormal function study |  |
| Inpatient ICD-9-CM diagnosis | D02V048Once | 997 | complications affecting specified body system not elsewhere classified |  |
| Inpatient ICD-9-CM diagnosis | D02V049Freq | V45 | other postprocedural states |  |
| Inpatient ICD-9-CM diagnosis | D02V049Once | V45 | other postprocedural states |  |
| Inpatient ICD-9-CM diagnosis | D02V049Spor | V45 | other postprocedural states |  |
| Inpatient ICD-9-CM diagnosis | D02V050Once | V67 | follow-up examination |  |
| Inpatient ICD-9-CM diagnosis | D02V054Once | 491 | chronic bronchitis |  |
| Inpatient ICD-9-CM diagnosis | D02V054Spor | 491 | chronic bronchitis |  |
| Inpatient ICD-9-CM diagnosis | D02V056Freq | 790 | abnormal blood findings |  |
| Inpatient ICD-9-CM diagnosis | D02V056Once | 790 | abnormal blood findings |  |
| Inpatient ICD-9-CM diagnosis | D02V056Spor | 790 | abnormal blood findings |  |
| Inpatient ICD-9-CM diagnosis | D02V057Freq | 793 | nonspecific (abnormal) findings on radiological and other examination of body structure |  |
| Inpatient ICD-9-CM diagnosis | D02V057Once | 793 | nonspecific (abnormal) findings on radiological and other examination of body structure |  |
| Inpatient ICD-9-CM diagnosis | D02V062Freq | 780 | general symptoms |  |
| Inpatient ICD-9-CM diagnosis | D02V062Once | 780 | general symptoms |  |
| Inpatient ICD-9-CM diagnosis | D02V062Spor | 780 | general symptoms |  |
| Inpatient ICD-9-CM diagnosis | D02V064Freq | 305 | nondependent drug abuse |  |
| Inpatient ICD-9-CM diagnosis | D02V064Once | 305 | nondependent drug abuse |  |
| Inpatient ICD-9-CM diagnosis | D02V065Once | 327 | organic sleep disorders |  |
| Inpatient ICD-9-CM diagnosis | D02V069Freq | 403 | hypertensive chronic kidney disease |  |
| Inpatient ICD-9-CM diagnosis | D02V069Once | 403 | hypertensive chronic kidney disease |  |
| Inpatient ICD-9-CM diagnosis | D02V071Freq | 412 | old myocardial infarction |  |
| Inpatient ICD-9-CM diagnosis | D02V071Once | 412 | old myocardial infarction |  |
| Inpatient ICD-9-CM diagnosis | D02V076Freq | 250 | diabetes mellitus |  |
| Inpatient ICD-9-CM diagnosis | D02V076Once | 250 | diabetes mellitus |  |
| Inpatient ICD-9-CM diagnosis | D02V076Spor | 250 | diabetes mellitus |  |
| Inpatient ICD-9-CM diagnosis | D02V079Once | V12 | personal history of certain other diseases |  |
| Inpatient ICD-9-CM diagnosis | D02V081Freq | 285 | other and unspecified anemias |  |
| Inpatient ICD-9-CM diagnosis | D02V081Once | 285 | other and unspecified anemias |  |
| Inpatient ICD-9-CM diagnosis | D02V081Spor | 285 | other and unspecified anemias |  |
| Inpatient ICD-9-CM diagnosis | D02V082Once | 599 | other disorders of urethra and urinary tract |  |
| Inpatient ICD-9-CM diagnosis | D02V090Once | 996 | complications peculiar to certain specified procedures |  |
| Inpatient ICD-9-CM diagnosis | D02V104Freq | 433 | precerebral occlusion |  |
| Inpatient ICD-9-CM diagnosis | D02V104Once | 433 | precerebral occlusion |  |
| Inpatient ICD-9-CM diagnosis | D02V105Once | 715 | osteoarthrosis and allied disorders |  |
| Inpatient ICD-9-CM diagnosis | D02V107Once | 492 | emphysema |  |
| Inpatient ICD-9-CM diagnosis | D02V108Once | V15 | other personal history presenting hazards to health |  |
| Inpatient ICD-9-CM diagnosis | D02V112Once | 278 | overweight, obesity and other hyperalimentation |  |
| Inpatient ICD-9-CM diagnosis | D02V129Once | 493 | asthma |  |
| Inpatient ICD-9-CM diagnosis | D02V130Freq | 514 | pulmonary congestion and hypostasis |  |
| Inpatient ICD-9-CM diagnosis | D02V130Once | 514 | pulmonary congestion and hypostasis |  |
| Inpatient ICD-9-CM diagnosis | D02V135Once | 402 | hypertensive heart disease |  |
| Inpatient ICD-9-CM diagnosis | D02V136Once | 441 | aortic aneurysm |  |
| Inpatient ICD-9-CM diagnosis | D02V136Spor | 441 | aortic aneurysm |  |
| Inpatient ICD-9-CM diagnosis | D02V139Once | 288 | diseases of white blood cells |  |
| Inpatient ICD-9-CM diagnosis | D02V139Spor | 288 | diseases of white blood cells |  |
| Inpatient ICD-9-CM diagnosis | D02V141Once | 998 | other complications of procedures not elsewhere classified |  |
| Inpatient ICD-9-CM diagnosis | D02V141Spor | 998 | other complications of procedures not elsewhere classified |  |
| Inpatient ICD-9-CM diagnosis | D02V147Once | 397 | diseases of other endocardial structures |  |
| Inpatient ICD-9-CM diagnosis | D02V155Freq | 426 | conduction disorders |  |
| Inpatient ICD-9-CM diagnosis | D02V155Once | 426 | conduction disorders |  |
| Inpatient ICD-9-CM diagnosis | D02V155Spor | 426 | conduction disorders |  |
| Inpatient ICD-9-CM diagnosis | D02V171Once | 396 | diseases of mitral and aortic valves |  |
| Inpatient ICD-9-CM diagnosis | D02V172Freq | 443 | other peripheral vascular disease |  |
| Inpatient ICD-9-CM diagnosis | D02V172Once | 443 | other peripheral vascular disease |  |
| Inpatient ICD-9-CM diagnosis | D02V173Freq | V72 | special examinations |  |
| Inpatient ICD-9-CM diagnosis | D02V173Once | V72 | special examinations |  |
| Inpatient ICD-9-CM diagnosis | D02V178Once | 423 | other diseases of pericardium |  |
| Inpatient ICD-9-CM diagnosis | D02V198Freq | 440 | atherosclerosis |  |
| Inpatient ICD-9-CM diagnosis | D02V198Once | 440 | atherosclerosis |  |
| Inpatient ICD-9-CM diagnosis | D02V210Freq | 413 | angina pectoris |  |
| Inpatient ICD-9-CM diagnosis | D02V210Once | 413 | angina pectoris |  |
| Inpatient ICD-9-CM diagnosis | D02V210Spor | 413 | angina pectoris |  |
| Inpatient ICD-10-CM diagnosis | D03V006Freq | I25 | chronic ischemic heart disease | Y |
| Inpatient ICD-10-CM diagnosis | D03V006Once | I25 | chronic ischemic heart disease |  |
| Inpatient ICD-10-CM diagnosis | D03V006Spor | I25 | chronic ischemic heart disease | Y |
| Inpatient ICD-19-CM diagnosis | D03V011Freq | Z95 | presence of cardiac and vascular implants and grafts |  |
| Inpatient ICD-10-CM diagnosis | D03V011Once | Z95 | presence of cardiac and vascular implants and grafts | Y |
| Inpatient ICD-10-CM diagnosis | D03V011Spor | Z95 | presence of cardiac and vascular implants and grafts |  |
| Inpatient ICD-10-CM diagnosis | D03V015Once | I42 | cardiomyopathy |  |
| Inpatient ICD-10-CM diagnosis | D03V015Spor | I42 | cardiomyopathy |  |
| Inpatient ICD-10-CM diagnosis | D03V016Freq | I50 | heart failure |  |
| Inpatient ICD-10-CM diagnosis | D03V016Once | I50 | heart failure |  |
| Inpatient ICD-10-CM diagnosis | D03V016Spor | I50 | heart failure |  |
| Inpatient ICD-10-CM diagnosis | D03V017Once | J95 | intraoperative and postprocedural complications and disorders of respiratory system, not elsewhere classified |  |
| Inpatient ICD-10-CM diagnosis | D03V018Freq | J98 | other respiratory disorders |  |
| Inpatient ICD-10-CM diagnosis | D03V018Once | J98 | other respiratory disorders | Y |
| Inpatient ICD-10-CM diagnosis | D03V018Spor | J98 | other respiratory disorders |  |
| Inpatient ICD-10-CM diagnosis | D03V021Once | R73 | elevated blood glucose level |  |
| Inpatient ICD-10-CM diagnosis | D03V022Once | Z46 | encounter for fitting and adjustment of other devices | Y |
| Inpatient ICD-10-CM diagnosis | D03V039Freq | N17 | acute kidney failure |  |
| Inpatient ICD-10-CM diagnosis | D03V039Once | N17 | acute kidney failure |  |
| Inpatient ICD-10-CM diagnosis | D03V039Spor | N17 | acute kidney failure |  |
| Inpatient ICD-10-CM diagnosis | D03V051Once | I13 | hypertensive heart and chronic kidney disease |  |
| Inpatient ICD-10-CM diagnosis | D03V054Freq | N18 | chronic kidney disease (ckd) |  |
| Inpatient ICD-10-CM diagnosis | D03V054Once | N18 | chronic kidney disease (ckd) |  |
| Inpatient ICD-10-CM diagnosis | D03V054Spor | N18 | chronic kidney disease (ckd) |  |
| Inpatient ICD-10-CM diagnosis | D03V056Once | Z98 | other postprocedural states |  |
| Inpatient ICD-10-CM diagnosis | D03V103Once | Z01 | encounter for other special examination without  complaint, suspected or reported diagnosis |  |
| Inpatient ICD-10-CM diagnosis | D03V113Freq | D62 | acute posthemorrhagic anemia |  |
| Inpatient ICD-10-CM diagnosis | D03V113Once | D62 | acute posthemorrhagic anemia |  |
| Inpatient ICD-10-CM diagnosis | D03V113Spor | D62 | acute posthemorrhagic anemia |  |
| Inpatient ICD-10-CM diagnosis | D03V158Once | Z48 | encounter for other postprocedural aftercare |  |
| Inpatient ICD-10-CM diagnosis | D03V158Spor | Z48 | encounter for other postprocedural aftercare |  |
| Inpatient ICD-9-CM procedure | D04V000Spor | 35 | operations on valves and septa of heart |  |
| Inpatient ICD-9-CM procedure | D04V001Freq | 36 | operations on vessels of heart |  |
| Inpatient ICD-9-CM procedure | D04V001Once | 36 | operations on vessels of heart |  |
| Inpatient ICD-9-CM procedure | D04V001Spor | 36 | operations on vessels of heart | Y |
| Inpatient ICD-9-CM procedure | D04V002Freq | 37 | other operations on heart and pericardium |  |
| Inpatient ICD-9-CM procedure | D04V002Once | 37 | other operations on heart and pericardium |  |
| Inpatient ICD-9-CM procedure | D04V003Spor | 39 | other operations on vessels | Y |
| Inpatient ICD-9-CM procedure | D04V004Freq | 88 | other diagnostic radiology and related techniques |  |
| Inpatient ICD-9-CM procedure | D04V004Once | 88 | other diagnostic radiology and related techniques |  |
| Inpatient ICD-9-CM procedure | D04V004Spor | 88 | other diagnostic radiology and related techniques |  |
| Inpatient ICD-9-CM procedure | D04V008Once | 96 | nonoperative intubation and irrigation |  |
| Inpatient ICD-9-CM procedure | D04V009Freq | 99 | other nonoperative procedures |  |
| Inpatient ICD-9-CM procedure | D04V009Once | 99 | other nonoperative procedures |  |
| Inpatient ICD-9-CM procedure | D04V011Once | 38 | incision, excision, and occlusion of vessels |  |
| Inpatient ICD-9-CM procedure | D04V013Once | 89 | interview, evaluation, consultation, and examination |  |
| Inpatient ICD-9-CM procedure | D04V018Once | 34 | operations on chest wall, pleura, mediastinum, and diaphragm |  |
| Inpatient ICD-10-CM procedure | D05V004Once | 4A0 | measurement |  |
| Inpatient ICD-10-CM procedure | D05V005Once | B21 | imaging: heart fluoroscopy |  |
| Inpatient ICD-10-CM procedure | D05V012Freq | 021 | bypass |  |
| Inpatient ICD-10-CM procedure | D05V012Once | 021 | bypass |  |
| Inpatient ICD-10-CM procedure | D05V012Spor | 021 | bypass |  |
| Inpatient ICD-10-CM procedure | D05V013Freq | 06B | excision |  |
| Inpatient ICD-10-CM procedure | D05V013Once | 06B | excision |  |
| Inpatient ICD-10-CM procedure | D05V014Freq | 5A1 | performance |  |
| Inpatient ICD-10-CM procedure | D05V014Once | 5A1 | performance |  |
| Inpatient ICD-10-CM procedure | D05V019Freq | 02R | heart and great vessels replacement |  |
| Inpatient ICD-10-CM procedure | D05V019Once | 02R | heart and great vessels replacement |  |
| Inpatient ICD-10-CM procedure | D05V032Freq | B24 | ultrasonography |  |
| Inpatient CPT-4/HCPCS procedure | D06V000Once | 00562 | anesthesia for procedures on heart, pericardial sac, and great vessels of chest | Y |
| Inpatient CPT-4/HCPCS procedure | D06V001Once | 33405 | replace aortic valve | Y |
| Inpatient CPT-4/HCPCS procedure | D06V004Once | 36620 | insert catheter, artery | Y |
| Inpatient CPT-4/HCPCS procedure | D06V005Freq | 71010 | chest x-ray |  |
| Inpatient CPT-4/HCPCS procedure | D06V005Once | 71010 | chest x-ray |  |
| Inpatient CPT-4/HCPCS procedure | D06V005Spor | 71010 | chest x-ray |  |
| Inpatient CPT-4/HCPCS procedure | D06V008Once | 88305 | tissue exam by pathologist | Y |
| Inpatient CPT-4/HCPCS procedure | D06V011Once | 93325 | doppler color flow add-on |  |
| Inpatient CPT-4/HCPCS procedure | D06V012Once | 93503 | insert/place heart catheter | Y |
| Inpatient CPT-4/HCPCS procedure | D06V013Once | 99100 | special anesthesia service |  |
| Inpatient CPT-4/HCPCS procedure | D06V014Freq | 71020 | chest x-ray |  |
| Inpatient CPT-4/HCPCS procedure | D06V014Once | 71020 | chest x-ray |  |
| Inpatient CPT-4/HCPCS procedure | D06V015Freq | 99223 | initial hospital care |  |
| Inpatient CPT-4/HCPCS procedure | D06V015Once | 99223 | initial hospital care |  |
| Inpatient CPT-4/HCPCS procedure | D06V016Freq | 99233 | subsequent hospital care |  |
| Inpatient CPT-4/HCPCS procedure | D06V016Once | 99233 | subsequent hospital care |  |
| Inpatient CPT-4/HCPCS procedure | D06V016Spor | 99233 | subsequent hospital care |  |
| Inpatient CPT-4/HCPCS procedure | D06V024Once | 99222 | initial hospital care |  |
| Inpatient CPT-4/HCPCS procedure | D06V025Once | 76770 | ultrasound, retroperitoneal (e.g., renal, aorta, nodes), real time with image documentation |  |
| Inpatient CPT-4/HCPCS procedure | D06V026Freq | 99291 | critical care, first hour |  |
| Inpatient CPT-4/HCPCS procedure | D06V026Once | 99291 | critical care, first hour |  |
| Inpatient CPT-4/HCPCS procedure | D06V026Spor | 99291 | critical care, first hour |  |
| Inpatient CPT-4/HCPCS procedure | D06V027Once | 93306 | transthoracic echocardiography |  |
| Inpatient CPT-4/HCPCS procedure | D06V028Freq | 99232 | subsequent hospital care |  |
| Inpatient CPT-4/HCPCS procedure | D06V028Once | 99232 | subsequent hospital care |  |
| Inpatient CPT-4/HCPCS procedure | D06V028Spor | 99232 | subsequent hospital care |  |
| Inpatient CPT-4/HCPCS procedure | D06V033Once | 74000 | x-ray abdomen |  |
| Inpatient CPT-4/HCPCS procedure | D06V035Once | 93970 | extremity study |  |
| Inpatient CPT-4/HCPCS procedure | D06V036Freq | 99231 | subsequent hospital care |  |
| Inpatient CPT-4/HCPCS procedure | D06V036Once | 99231 | subsequent hospital care |  |
| Inpatient CPT-4/HCPCS procedure | D06V036Spor | 99231 | subsequent hospital care |  |
| Inpatient CPT-4/HCPCS procedure | D06V040Once | 93510 | left heart catheterization |  |
| Inpatient CPT-4/HCPCS procedure | D06V041Once | 93543 | inject for heart x-rays |  |
| Inpatient CPT-4/HCPCS procedure | D06V042Once | 93545 | inject for coronary x-rays |  |
| Inpatient CPT-4/HCPCS procedure | D06V043Once | 93555 | imaging, cardiac catheterization |  |
| Inpatient CPT-4/HCPCS procedure | D06V044Once | 93556 | imaging, cardiac catheterization |  |
| Inpatient CPT-4/HCPCS procedure | D06V045Once | 00567 | anesthesia for intrathoracic procedures |  |
| Inpatient CPT-4/HCPCS procedure | D06V046Once | 33508 | endoscopic vein harvest |  |
| Inpatient CPT-4/HCPCS procedure | D06V047Once | 33519 | coronary artery bypass, using venous graft(s) and arterial graft(s) |  |
| Inpatient CPT-4/HCPCS procedure | D06V048Once | 33533 | arterial grafting for coronary artery bypass |  |
| Inpatient CPT-4/HCPCS procedure | D06V049Once | 4048F | therapeutic, preventive or other interventions |  |
| Inpatient CPT-4/HCPCS procedure | D06V050Freq | 93010 | ecg report |  |
| Inpatient CPT-4/HCPCS procedure | D06V050Once | 93010 | ecg report |  |
| Inpatient CPT-4/HCPCS procedure | D06V050Spor | 93010 | ecg report |  |
| Inpatient CPT-4/HCPCS procedure | D06V051Once | 93320 | doppler echo exam heart |  |
| Inpatient CPT-4/HCPCS procedure | D06V057Once | 71275 | angiography, chest |  |
| Inpatient CPT-4/HCPCS procedure | D06V061Freq | 99254 | inpatient consultation |  |
| Inpatient CPT-4/HCPCS procedure | D06V061Once | 99254 | inpatient consultation |  |
| Inpatient CPT-4/HCPCS procedure | D06V063Once | 71090 | x-ray & pacemaker insert |  |
| Inpatient CPT-4/HCPCS procedure | D06V064Once | 99255 | inpatient consultation |  |
| Inpatient CPT-4/HCPCS procedure | D06V081Once | 93458 | catheter placement in coronary artery(s) for coronary angiography |  |
| Inpatient CPT-4/HCPCS procedure | D06V084Once | 99253 | inpatient consultation |  |
| Inpatient CPT-4/HCPCS procedure | D06V134Once | 93880 | extracranial study |  |
| Inpatient CPT-4/HCPCS procedure | D06V140Freq | 71045 | radiologic examination, chest |  |
| Inpatient CPT-4/HCPCS procedure | D06V140Once | 71045 | radiologic examination, chest |  |
| Inpatient CPT-4/HCPCS procedure | D06V140Spor | 71045 | radiologic examination, chest |  |
| Inpatient CPT-4/HCPCS procedure | D06V141Freq | 71046 | radiologic examination, chest |  |
| Inpatient CPT-4/HCPCS procedure | D06V141Once | 71046 | radiologic examination, chest |  |
| Inpatient CPT-4/HCPCS procedure | D06V174Once | 93321 | doppler echo exam heart |  |
| Inpatient CPT-4/HCPCS procedure | D06V218Once | 93307 | echo exam heart |  |
| Inpatient CPT-4/HCPCS procedure | D06V227Once | 33518 | coronary artery bypass, using venous graft(s) and arterial graft(s) |  |
| Inpatient CPT-4/HCPCS procedure | D06V237Once | 33534 | coronary artery bypass, using arterial graft(s) |  |
| Inpatient CPT-4/HCPCS procedure | D06V239Once | 93308 | echo exam heart |  |
| Inpatient CPT-4/HCPCS procedure | D06V241Once | 93454 | catheter placement in coronary artery(s) for coronary angiography |  |
| Inpatient CPT-4/HCPCS procedure | D06V247Once | 92980 | insert intracoronary stent |  |
| Inpatient CPT-4/HCPCS procedure | D06V283Once | 93526 | combined right heart catheterization and retrograde left heart catheterization |  |
| Inpatient CPT-4/HCPCS procedure | D06V404Once | 59510 | cesarean delivery |  |
| Inpatient CPT-4/HCPCS procedure | D06V422Once | 33967 | insertion of intra-aortic balloon assist device |  |
| Inpatient CPT-4/HCPCS procedure | D06V463Once | 93459 | catheter placement in coronary artery(s) for coronary angiography |  |
| Inpatient CPT-4/HCPCS procedure | D06V639Once | 76998 | other diagnostic ultrasound procedures |  |
| Outpatient ICD-9-CM diagnosis | D07V002Once | 702 | other dermatoses | Y |
| Outpatient ICD-9-CM diagnosis | D07V002Spor | 702 | other dermatoses | Y |
| Outpatient ICD-9-CM diagnosis | D07V004Freq | 272 | disorders of lipoid metabolism |  |
| Outpatient ICD-9-CM diagnosis | D07V004Once | 272 | disorders of lipoid metabolism |  |
| Outpatient ICD-9-CM diagnosis | D07V004Spor | 272 | disorders of lipoid metabolism |  |
| Outpatient ICD-9-CM diagnosis | D07V005Freq | 401 | essential hypertension |  |
| Outpatient ICD-9-CM diagnosis | D07V005Once | 401 | essential hypertension |  |
| Outpatient ICD-9-CM diagnosis | D07V005Spor | 401 | essential hypertension |  |
| Outpatient ICD-9-CM diagnosis | D07V006Freq | 424 | other diseases of endocardium |  |
| Outpatient ICD-9-CM diagnosis | D07V006Once | 424 | other diseases of endocardium |  |
| Outpatient ICD-9-CM diagnosis | D07V006Spor | 424 | other diseases of endocardium |  |
| Outpatient ICD-9-CM diagnosis | D07V007Freq | 427 | cardiac dysrhythmias |  |
| Outpatient ICD-9-CM diagnosis | D07V007Once | 427 | cardiac dysrhythmias |  |
| Outpatient ICD-9-CM diagnosis | D07V007Spor | 427 | cardiac dysrhythmias |  |
| Outpatient ICD-9-CM diagnosis | D07V010Freq | 780 | general symptoms |  |
| Outpatient ICD-9-CM diagnosis | D07V010Once | 780 | general symptoms |  |
| Outpatient ICD-9-CM diagnosis | D07V010Spor | 780 | general symptoms |  |
| Outpatient ICD-9-CM diagnosis | D07V017Once | 600 | hyperplasia of prostate |  |
| Outpatient ICD-9-CM diagnosis | D07V018Freq | 414 | other forms of chronic ischemic heart disease |  |
| Outpatient ICD-9-CM diagnosis | D07V018Once | 414 | other forms of chronic ischemic heart disease |  |
| Outpatient ICD-9-CM diagnosis | D07V018Spor | 414 | other forms of chronic ischemic heart disease |  |
| Outpatient ICD-9-CM diagnosis | D07V020Once | V58 | encounter for other and unspecified procedures and aftercare |  |
| Outpatient ICD-9-CM diagnosis | D07V020Spor | V58 | encounter for other and unspecified procedures and aftercare |  |
| Outpatient ICD-9-CM diagnosis | D07V023Freq | 250 | diabetes mellitus |  |
| Outpatient ICD-9-CM diagnosis | D07V023Once | 250 | diabetes mellitus |  |
| Outpatient ICD-9-CM diagnosis | D07V023Spor | 250 | diabetes mellitus |  |
| Outpatient ICD-9-CM diagnosis | D07V024Freq | 428 | heart failure |  |
| Outpatient ICD-9-CM diagnosis | D07V024Once | 428 | heart failure |  |
| Outpatient ICD-9-CM diagnosis | D07V024Spor | 428 | heart failure |  |
| Outpatient ICD-9-CM diagnosis | D07V026Freq | 429 | ill-defined descriptions and complications of heart disease |  |
| Outpatient ICD-9-CM diagnosis | D07V026Once | 429 | ill-defined descriptions and complications of heart disease |  |
| Outpatient ICD-9-CM diagnosis | D07V028Freq | 426 | conduction disorders |  |
| Outpatient ICD-9-CM diagnosis | D07V028Once | 426 | conduction disorders |  |
| Outpatient ICD-9-CM diagnosis | D07V029Freq | V45 | other postprocedural states |  |
| Outpatient ICD-9-CM diagnosis | D07V029Once | V45 | other postprocedural states |  |
| Outpatient ICD-9-CM diagnosis | D07V029Spor | V45 | other postprocedural states |  |
| Outpatient ICD-9-CM diagnosis | D07V030Freq | 785 | symptoms involving cardiovascular system |  |
| Outpatient ICD-9-CM diagnosis | D07V030Once | 785 | symptoms involving cardiovascular system |  |
| Outpatient ICD-9-CM diagnosis | D07V031Once | 366 | cataract |  |
| Outpatient ICD-9-CM diagnosis | D07V033Once | 791 | abnormal urine findings |  |
| Outpatient ICD-9-CM diagnosis | D07V035Once | 790 | abnormal blood findings |  |
| Outpatient ICD-9-CM diagnosis | D07V036Freq | 496 | chronic airway obstruction, not elsewhere classified |  |
| Outpatient ICD-9-CM diagnosis | D07V036Once | 496 | chronic airway obstruction, not elsewhere classified |  |
| Outpatient ICD-9-CM diagnosis | D07V036Spor | 496 | chronic airway obstruction, not elsewhere classified |  |
| Outpatient ICD-9-CM diagnosis | D07V037Once | 491 | chronic bronchitis |  |
| Outpatient ICD-9-CM diagnosis | D07V037Spor | 491 | chronic bronchitis |  |
| Outpatient ICD-9-CM diagnosis | D07V038Freq | 786 | symptoms involving respiratory system and other chest symptoms |  |
| Outpatient ICD-9-CM diagnosis | D07V038Once | 786 | symptoms involving respiratory system and other chest symptoms |  |
| Outpatient ICD-9-CM diagnosis | D07V038Spor | 786 | symptoms involving respiratory system and other chest symptoms |  |
| Outpatient ICD-9-CM diagnosis | D07V046Freq | 441 | aortic aneurysm |  |
| Outpatient ICD-9-CM diagnosis | D07V046Once | 441 | aortic aneurysm |  |
| Outpatient ICD-9-CM diagnosis | D07V046Spor | 441 | aortic aneurysm |  |
| Outpatient ICD-9-CM diagnosis | D07V048Once | V04 | need for prophylactic vaccination and inoculation against certain viral diseases |  |
| Outpatient ICD-9-CM diagnosis | D07V052Once | 799 | other ill-defined and unknown causes of morbidity and mortality |  |
| Outpatient ICD-9-CM diagnosis | D07V057Freq | 403 | hypertensive chronic kidney disease |  |
| Outpatient ICD-9-CM diagnosis | D07V057Once | 403 | hypertensive chronic kidney disease |  |
| Outpatient ICD-9-CM diagnosis | D07V057Spor | 403 | hypertensive chronic kidney disease |  |
| Outpatient ICD-9-CM diagnosis | D07V060Freq | 585 | chronic renal failure |  |
| Outpatient ICD-9-CM diagnosis | D07V060Once | 585 | chronic renal failure |  |
| Outpatient ICD-9-CM diagnosis | D07V060Spor | 585 | chronic renal failure |  |
| Outpatient ICD-9-CM diagnosis | D07V062Once | V15 | other personal history presenting hazards to health |  |
| Outpatient ICD-9-CM diagnosis | D07V063Freq | 794 | abnormal function study |  |
| Outpatient ICD-9-CM diagnosis | D07V063Once | 794 | abnormal function study |  |
| Outpatient ICD-9-CM diagnosis | D07V065Freq | 410 | acute myocardial infarction |  |
| Outpatient ICD-9-CM diagnosis | D07V065Once | 410 | acute myocardial infarction |  |
| Outpatient ICD-9-CM diagnosis | D07V066Freq | 518 | other lung diseases |  |
| Outpatient ICD-9-CM diagnosis | D07V066Once | 518 | other lung diseases |  |
| Outpatient ICD-9-CM diagnosis | D07V070Freq | 285 | other and unspecified anemias |  |
| Outpatient ICD-9-CM diagnosis | D07V070Once | 285 | other and unspecified anemias |  |
| Outpatient ICD-9-CM diagnosis | D07V070Spor | 285 | other and unspecified anemias |  |
| Outpatient ICD-9-CM diagnosis | D07V079Once | 305 | nondependent drug abuse |  |
| Outpatient ICD-9-CM diagnosis | D07V096Once | 793 | nonspecific (abnormal) findings on radiological and other examination of body structure |  |
| Outpatient ICD-9-CM diagnosis | D07V106Once | 402 | hypertensive heart disease |  |
| Outpatient ICD-9-CM diagnosis | D07V118Once | 584 | acute renal failure |  |
| Outpatient ICD-9-CM diagnosis | D07V122Freq | 433 | precerebral occlusion |  |
| Outpatient ICD-9-CM diagnosis | D07V122Once | 433 | precerebral occlusion |  |
| Outpatient ICD-9-CM diagnosis | D07V124Freq | 413 | angina pectoris |  |
| Outpatient ICD-9-CM diagnosis | D07V124Once | 413 | angina pectoris |  |
| Outpatient ICD-9-CM diagnosis | D07V125Once | 443 | other peripheral vascular disease |  |
| Outpatient ICD-9-CM diagnosis | D07V125Spor | 443 | other peripheral vascular disease |  |
| Outpatient ICD-9-CM diagnosis | D07V128Freq | 486 | pneumonia |  |
| Outpatient ICD-9-CM diagnosis | D07V128Once | 486 | pneumonia |  |
| Outpatient ICD-9-CM diagnosis | D07V129Freq | 511 | pleurisy |  |
| Outpatient ICD-9-CM diagnosis | D07V129Once | 511 | pleurisy |  |
| Outpatient ICD-9-CM diagnosis | D07V149Once | 715 | osteoarthrosis and allied disorders | Y |
| Outpatient ICD-9-CM diagnosis | D07V156Once | 466 | acute bronchitis and bronchiolitis |  |
| Outpatient ICD-9-CM diagnosis | D07V158Once | V43 | organ or tissue replaced by other means |  |
| Outpatient ICD-9-CM diagnosis | D07V174Freq | 593 | other disorders of kidney and ureter |  |
| Outpatient ICD-9-CM diagnosis | D07V174Once | 593 | other disorders of kidney and ureter |  |
| Outpatient ICD-9-CM diagnosis | D07V179Once | 492 | emphysema |  |
| Outpatient ICD-9-CM diagnosis | D07V187Freq | 412 | old myocardial infarction |  |
| Outpatient ICD-9-CM diagnosis | D07V187Once | 412 | old myocardial infarction |  |
| Outpatient ICD-9-CM diagnosis | D07V197Freq | 440 | atherosclerosis |  |
| Outpatient ICD-9-CM diagnosis | D07V197Once | 440 | atherosclerosis |  |
| Outpatient ICD-9-CM diagnosis | D07V234Once | 514 | pulmonary congestion and hypostasis |  |
| Outpatient ICD-9-CM diagnosis | D07V238Once | 416 | chronic pulmonary heart disease |  |
| Outpatient ICD-9-CM diagnosis | D07V243Freq | 411 | other acute and subacute forms of ischemic heart disease |  |
| Outpatient ICD-9-CM diagnosis | D07V243Once | 411 | other acute and subacute forms of ischemic heart disease |  |
| Outpatient ICD-9-CM diagnosis | D07V338Once | 425 | cardiomyopathy |  |
| Outpatient ICD-9-CM diagnosis | D07V357Once | 274 | gout |  |
| Outpatient ICD-10-CM diagnosis | D08V003Freq | E11 | type 2 diabetes mellitus | Y |
| Outpatient ICD-10-CM diagnosis | D08V003Once | E11 | type 2 diabetes mellitus | Y |
| Outpatient ICD-10-CM diagnosis | D08V003Spor | E11 | type 2 diabetes mellitus | Y |
| Outpatient ICD-10-CM diagnosis | D08V005Once | I10 | essential (primary) hypertension | Y |
| Outpatient ICD-10-CM diagnosis | D08V005Spor | I10 | essential (primary) hypertension | Y |
| Outpatient ICD-10-CM diagnosis | D08V006Once | I25 | chronic ischemic heart disease |  |
| Outpatient ICD-10-CM diagnosis | D08V006Spor | I25 | chronic ischemic heart disease |  |
| Outpatient ICD-10-CM diagnosis | D08V007Once | Z00 | encounter for general examination without complaint, suspected or reported diagnosis |  |
| Outpatient ICD-10-CM diagnosis | D08V010Once | Z23 | encounter for immunization |  |
| Outpatient ICD-10-CM diagnosis | D08V016Once | E66 | overweight and obesity |  |
| Outpatient ICD-10-CM diagnosis | D08V017Freq | E87 | other disorders of fluid, electrolyte and acid-base balance |  |
| Outpatient ICD-10-CM diagnosis | D08V017Once | E87 | other disorders of fluid, electrolyte and acid-base balance |  |
| Outpatient ICD-10-CM diagnosis | D08V024Once | Z68 | body mass index [bmi] |  |
| Outpatient ICD-10-CM diagnosis | D08V032Once | M17 | osteoarthritis of knee |  |
| Outpatient ICD-10-CM diagnosis | D08V032Spor | M17 | osteoarthritis of knee |  |
| Outpatient ICD-10-CM diagnosis | D08V033Once | M19 | other and unspecified osteoarthritis |  |
| Outpatient ICD-10-CM diagnosis | D08V035Once | J44 | other chronic obstructive pulmonary disease |  |
| Outpatient ICD-10-CM diagnosis | D08V035Spor | J44 | other chronic obstructive pulmonary disease |  |
| Outpatient ICD-10-CM diagnosis | D08V047Once | J96 | respiratory failure, not elsewhere classified |  |
| Outpatient ICD-10-CM diagnosis | D08V047Spor | J96 | respiratory failure, not elsewhere classified |  |
| Outpatient ICD-10-CM diagnosis | D08V049Freq | M54 | dorsalgia |  |
| Outpatient ICD-10-CM diagnosis | D08V049Once | M54 | dorsalgia |  |
| Outpatient ICD-10-CM diagnosis | D08V049Spor | M54 | dorsalgia |  |
| Outpatient ICD-10-CM diagnosis | D08V055Once | M25 | other joint disorder, not elsewhere classified |  |
| Outpatient ICD-10-CM diagnosis | D08V055Spor | M25 | other joint disorder, not elsewhere classified |  |
| Outpatient ICD-10-CM diagnosis | D08V056Freq | M79 | other and unspecified soft tissue disorders, not elsewhere classified |  |
| Outpatient ICD-10-CM diagnosis | D08V056Once | M79 | other and unspecified soft tissue disorders, not elsewhere classified |  |
| Outpatient ICD-10-CM diagnosis | D08V056Spor | M79 | other and unspecified soft tissue disorders, not elsewhere classified |  |
| Outpatient ICD-10-CM diagnosis | D08V061Freq | N18 | chronic kidney disease (ckd) |  |
| Outpatient ICD-10-CM diagnosis | D08V061Once | N18 | chronic kidney disease (ckd) |  |
| Outpatient ICD-10-CM diagnosis | D08V061Spor | N18 | chronic kidney disease (ckd) |  |
| Outpatient ICD-10-CM diagnosis | D08V062Once | R60 | edema, not elsewhere classified |  |
| Outpatient ICD-10-CM diagnosis | D08V064Once | H25 | age-related cataract |  |
| Outpatient ICD-10-CM diagnosis | D08V069Freq | I12 | hypertensive chronic kidney disease |  |
| Outpatient ICD-10-CM diagnosis | D08V069Once | I12 | hypertensive chronic kidney disease |  |
| Outpatient ICD-10-CM diagnosis | D08V069Spor | I12 | hypertensive chronic kidney disease |  |
| Outpatient ICD-10-CM diagnosis | D08V071Freq | D50 | iron deficiency anemia |  |
| Outpatient ICD-10-CM diagnosis | D08V081Once | Z95 | presence of cardiac and vascular implants and grafts |  |
| Outpatient ICD-10-CM diagnosis | D08V082Once | J18 | pneumonia, unspecified organism |  |
| Outpatient ICD-10-CM diagnosis | D08V094Once | K76 | other diseases of liver |  |
| Outpatient ICD-10-CM diagnosis | D08V118Freq | I42 | cardiomyopathy |  |
| Outpatient ICD-10-CM diagnosis | D08V118Once | I42 | cardiomyopathy |  |
| Outpatient ICD-10-CM diagnosis | D08V118Spor | I42 | cardiomyopathy |  |
| Outpatient ICD-10-CM diagnosis | D08V119Freq | I50 | heart failure |  |
| Outpatient ICD-10-CM diagnosis | D08V119Once | I50 | heart failure |  |
| Outpatient ICD-10-CM diagnosis | D08V119Spor | I50 | heart failure |  |
| Outpatient ICD-10-CM diagnosis | D08V136Once | R10 | abdominal and pelvic pain | Y |
| Outpatient ICD-10-CM diagnosis | D08V146Once | H52 | disorders of refraction and accommodation |  |
| Outpatient ICD-10-CM diagnosis | D08V150Once | D64 | other anemias | Y |
| Outpatient ICD-10-CM diagnosis | D08V150Spor | D64 | other anemias | Y |
| Outpatient ICD-10-CM diagnosis | D08V156Freq | Z12 | encounter for screening for malignant neoplasms |  |
| Outpatient ICD-10-CM diagnosis | D08V156Once | Z12 | encounter for screening for malignant neoplasms |  |
| Outpatient ICD-10-CM diagnosis | D08V163Freq | E55 | vitamin d deficiency |  |
| Outpatient ICD-10-CM diagnosis | D08V163Once | E55 | vitamin d deficiency |  |
| Outpatient ICD-10-CM diagnosis | D08V179Freq | Z01 | encounter for other special examination without complaint, suspected or reported diagnosis |  |
| Outpatient ICD-10-CM diagnosis | D08V180Freq | Z99 | dependence on enabling machines and devices, not elsewhere classified |  |
| Outpatient ICD-10-CM diagnosis | D08V180Once | Z99 | dependence on enabling machines and devices, not elsewhere classified |  |
| Outpatient ICD-10-CM diagnosis | D08V180Spor | Z99 | dependence on enabling machines and devices, not elsewhere classified |  |
| Outpatient ICD-10-CM diagnosis | D08V184Once | I21 | acute myocardial infarction |  |
| Outpatient ICD-10-CM diagnosis | D08V190Once | G89 | pain, not elsewhere classified |  |
| Outpatient ICD-10-CM diagnosis | D08V214Freq | N17 | acute kidney failure |  |
| Outpatient ICD-10-CM diagnosis | D08V214Once | N17 | acute kidney failure |  |
| Outpatient ICD-10-CM diagnosis | D08V214Spor | N17 | acute kidney failure |  |
| Outpatient ICD-10-CM diagnosis | D08V218Once | J81 | pulmonary edema |  |
| Outpatient ICD-10-CM diagnosis | D08V224Freq | I47 | paroxysmal tachycardia |  |
| Outpatient ICD-10-CM diagnosis | D08V224Once | I47 | paroxysmal tachycardia |  |
| Outpatient ICD-10-CM diagnosis | D08V224Spor | I47 | paroxysmal tachycardia |  |
| Outpatient ICD-10-CM diagnosis | D08V250Freq | D63 | anemia in chronic diseases classified elsewhere |  |
| Outpatient ICD-10-CM diagnosis | D08V250Once | D63 | anemia in chronic diseases classified elsewhere |  |
| Outpatient ICD-10-CM diagnosis | D08V250Spor | D63 | anemia in chronic diseases classified elsewhere |  |
| Outpatient ICD-10-CM diagnosis | D08V334Once | Z45 | encounter for adjustment and management of implanted device |  |
| Outpatient CPT-4/HCPCS procedure | D11V002Freq | 93000 | ecg complete |  |
| Outpatient CPT-4/HCPCS procedure | D11V002Once | 93000 | ecg complete |  |
| Outpatient CPT-4/HCPCS procedure | D11V003Once | 93306 | electrocardiogram, routine ecg with at least 12 leads |  |
| Outpatient CPT-4/HCPCS procedure | D11V004Spor | 99214 | established patient office visit, 30-39 minute | Y |
| Outpatient CPT-4/HCPCS procedure | D11V015Freq | 36415 | routine venipuncture | Y |
| Outpatient CPT-4/HCPCS procedure | D11V015Spor | 36415 | routine venipuncture | Y |
| Outpatient CPT-4/HCPCS procedure | D11V016Freq | 80048 | basic metabolic panel (calcium total) | Y |
| Outpatient CPT-4/HCPCS procedure | D11V025Once | J3010 | injection, fentanyl citrate, 0.1 mg | Y |
| Outpatient CPT-4/HCPCS procedure | D11V026Once | Q9967 | low osmolar contrast material, 300-399 mg/ml iodine concentration, per ml | Y |
| Outpatient CPT-4/HCPCS procedure | D11V027Freq | 71020 | chest x-ray |  |
| Outpatient CPT-4/HCPCS procedure | D11V027Once | 71020 | chest x-ray |  |
| Outpatient CPT-4/HCPCS procedure | D11V029Freq | 83036 | glycosylated hemoglobin test | Y |
| Outpatient CPT-4/HCPCS procedure | D11V029Once | 83036 | glycosylated hemoglobin test | Y |
| Outpatient CPT-4/HCPCS procedure | D11V029Spor | 83036 | glycosylated hemoglobin test | Y |
| Outpatient CPT-4/HCPCS procedure | D11V039Once | 99285 | emergency department visit |  |
| Outpatient CPT-4/HCPCS procedure | D11V040Freq | 93010 | ecg report |  |
| Outpatient CPT-4/HCPCS procedure | D11V040Once | 93010 | ecg report |  |
| Outpatient CPT-4/HCPCS procedure | D11V057Once | 82043 | microalbumin, quantitative | Y |
| Outpatient CPT-4/HCPCS procedure | D11V058Freq | 82570 | assay urine creatinine | Y |
| Outpatient CPT-4/HCPCS procedure | D11V058Once | 82570 | assay urine creatinine | Y |
| Outpatient CPT-4/HCPCS procedure | D11V062Freq | 71010 | chest x-ray |  |
| Outpatient CPT-4/HCPCS procedure | D11V062Once | 71010 | chest x-ray |  |
| Outpatient CPT-4/HCPCS procedure | D11V064Once | 85610 | prothrombin time |  |
| Outpatient CPT-4/HCPCS procedure | D11V070Once | A0425 | ground mileage, per statute mile |  |
| Outpatient CPT-4/HCPCS procedure | D11V071Once | A0427 | ambulance service, advanced life support, emergency transport, level 1 (ALS 1-emergency) |  |
| Outpatient CPT-4/HCPCS procedure | D11V078Once | 82962 | glucose blood test | Y |
| Outpatient CPT-4/HCPCS procedure | D11V085Once | 94760 | measure blood oxygen level |  |
| Outpatient CPT-4/HCPCS procedure | D11V105Once | 86850 | rbc antibody screen |  |
| Outpatient CPT-4/HCPCS procedure | D11V106Once | 86900 | blood typing, serologic |  |
| Outpatient CPT-4/HCPCS procedure | D11V107Once | 86901 | blood typing, rh (d) |  |
| Outpatient CPT-4/HCPCS procedure | D11V113Once | 99291 | critical care, first hour |  |
| Outpatient CPT-4/HCPCS procedure | D11V134Once | 78452 | myocardial perfusion imaging, tomographic | Y |
| Outpatient CPT-4/HCPCS procedure | D11V139Once | 90658 | influenza virus vaccine, trivalent (IIV3), split virus, 0.5 mL dosage, for intramuscular use |  |
| Outpatient CPT-4/HCPCS procedure | D11V143Once | 80050 | general health panel |  |
| Outpatient CPT-4/HCPCS procedure | D11V144Once | 84153 | assay psa, total |  |
| Outpatient CPT-4/HCPCS procedure | D11V181Once | A9500 | technetium Tc-99m sestamibi, diagnostic, per study dose | Y |
| Outpatient CPT-4/HCPCS procedure | D11V185Once | G0463 | hospital outpatient clinic visit or assessment and management of a patient |  |
| Outpatient CPT-4/HCPCS procedure | D11V185Spor | G0463 | hospital outpatient clinic visit or assessment and management of a patient | Y |
| Outpatient CPT-4/HCPCS procedure | D11V204Freq | 80069 | renal function panel |  |
| Outpatient CPT-4/HCPCS procedure | D11V204Once | 80069 | renal function panel | Y |
| Outpatient CPT-4/HCPCS procedure | D11V232Once | 82306 | assay vitamin d | Y |
| Outpatient CPT-4/HCPCS procedure | D11V236Once | 93015 | cardiovascular stress test |  |
| Outpatient CPT-4/HCPCS procedure | D11V245Once | 93880 | extracranial study | Y |
| Outpatient CPT-4/HCPCS procedure | D11V290Once | A4253 | blood glucose test or reagent strips for home blood glucose monitor, per 50 strips |  |
| Outpatient CPT-4/HCPCS procedure | D11V375Freq | 83970 | assay parathormone | Y |
| Outpatient CPT-4/HCPCS procedure | D11V375Once | 83970 | assay parathormone | Y |
| Outpatient CPT-4/HCPCS procedure | D11V385Freq | 84100 | assay phosphorus | Y |
| Outpatient CPT-4/HCPCS procedure | D11V385Once | 84100 | assay phosphorus | Y |
| Outpatient CPT-4/HCPCS procedure | D11V412Once | A9502 | technetium Tc-99m tetrofosmin, diagnostic, per study dose |  |
| Outpatient CPT-4/HCPCS procedure | D11V421Once | 11721 | debride nail, 6 or more |  |
| Outpatient CPT-4/HCPCS procedure | D11V535Once | 76770 | ultrasound, retroperitoneal (e.g., renal, aorta, nodes), real time with image documentation | Y |
| Outpatient CPT-4/HCPCS procedure | D11V631Once | 93320 | doppler echo exam heart |  |
| Outpatient CPT-4/HCPCS procedure | D11V632Once | 93325 | doppler color flow add-on |  |
| Laboratory | D17V053Once | 11580-8 | thyrotropin [units/volume] in serum or plasma by detection limit <= 0.005 miu/l |  |
| Laboratory | D17V059Once | 62238-1 | glomerular filtration rate/1.73 sq m.predicted [volume rate/area] in serum or plasma by creatinine-b |  |
| Laboratory | D17V060Freq | 88293-6 | glomerular filtration rate/1.73 sq m.predicted among blacks [volume rate/area] in serum, plasma or blood by creatinine-based formula (ckd-epi) |  |
| Laboratory | D17V060Once | 88293-6 | glomerular filtration rate/1.73 sq m.predicted among blacks [volume rate/area] in serum, plasma or blood by creatinine-based formula (ckd-epi) |  |
| Laboratory | D17V061Freq | 88294-4 | glomerular filtration rate/1.73 sq m.predicted among non-blacks [volume rate/area] in serum, plasma or blood by creatinine-based formula (ckd-epi) |  |
| Laboratory | D17V061Once | 88294-4 | glomerular filtration rate/1.73 sq m.predicted among non-blacks [volume rate/area] in serum, plasma or blood by creatinine-based formula (ckd-epi) |  |
| Laboratory | D17V163Once | 62292-8 | hydroxyvitamin d2+25-hydroxyvitamin d3  [mass/volume] in serum or plasma |  |
