# Supplementary material for: Impact of empiric potassium supplementation on mortality, sudden cardiac arrest and stroke in furosemide initiators
Source: Br J Clin Pharmacol. 2026 May 3;92(8):2924–36. doi: 10.1002/bcp.70584 (PMC13421057; doi:10.1002/bcp.70584)
Supplement: Supplementary file 13 — Table S6. Baseline characteristics among individuals initiating furosemide <40 mg/day or ≥40 mg/day with and without empiric potassium in the remaining cohort after PS trimming. [file BCP-92-2924-s017.docx]

**Table S6. Baseline characteristics among individuals initiating furosemide <40 mg/day or ≥40 mg/day with and without empiric potassium in the remaining cohort after PS trimming**

| **Characteristics** | **Furosemide <40 mg/day** | | | | | | **Furosemide ≥40 mg/day** | | | | | |
| --- | --- | --- | --- | --- | --- | --- | --- | --- | --- | --- | --- | --- |
|  | **Before IPTW** | | | **After IPTW** | | | **Before IPTW** | | | **After IPTW** | | |
|  | **K+ = NO**  **(n=349,693)** | **K+ = YES**  **(n=96,085)** | **SDiff^†^** | **K+ = NO**  **(n=445,843)** | **K+ = YES**  **(n=445,340)** | **Weighted SDiff^†^** | **K+ = NO**  **(n=177,580)** | **K+ = YES**  **(n=93,186)** | **SDiff^†^** | **K+ = NO**  **(n=270,920)** | **K+ = YES**  **(n=270,494)** | **Weighted SDiff^†^** |
| **Propensity score, mean (SD)** | 0.21 (0.08) | 0.24 (0.08) | **0.48** | 0.22 (0.09) | 0.22 (0.17) | 0.00 | 0.33 (0.11) | 0.38 (0.11) | **0.50** | 0.34 (0.14) | 0.34 (0.19) | 0.00 |
| **Demographic** | | | | | | | | | | | | |
| Age in years, median (IQR) | 72.19  (60.54-80.92) | 73.02  (61.57-81.33) | 0.05 | 72.40  (60.79-81.01) | 72.27  (60.56-80.97) | -0.00 | 70.63  (59.62-79.39) | 71.53  (60.44-79.91) | 0.05 | 70.88  (59.96-79.57) | 70.94  (59.70-79.63) | -0.00 |
| Age groups, % col |  |  | 0.06 |  |  | 0.00 |  |  | 0.07 |  |  | 0.04 |
| <35 years | 2.3% | 1.8% |  | 2.3% | 1.8% |  | 2.1% | 1.6% |  | 2.1% | 1.7% |  |
| 35-44 years | 5.1% | 4.7% |  | 5.0% | 5.1% |  | 4.9% | 4.5% |  | 4.7% | 5.0% |  |
| 45-54 years | 9.9% | 9.5% |  | 9.7% | 10.1% |  | 10.7% | 10.3%) |  | 10.4% | 10.9% |  |
| 55-64 years | 15.0% | 14.6% |  | 14.8% | 15.2% |  | 17.5% | 16.8% |  | 17.3% | 17.2% |  |
| 65-74 years | 25.4% | 24.8% |  | 25.3% | 25.2% |  | 27.2% | 26.7% |  | 27.2% | 26.8% |  |
| 75-84 years | 29.2% | 31.1% |  | 29.6% | 29.5% |  | 27.1% | 29.5% |  | 27.8% | 28.1% |  |
| 85+ years | 13.2% | 13.5% |  | 13.3% | 13.0% |  | 10.4% | 10.7% |  | 10.6% | 10.4% |  |
| Female sex, %col | 61.7% | 62.0% | 0.06 | 61.7% | 61.8% | 0.00 | 51.7% | 52.1% | 0.01 | 51.8% | 51.9% | 0.00 |
| Race, %col |  |  | 0.07 |  |  | 0.00 |  |  | 0.07 |  |  | 0.00 |
| White | 66.74% | 68.91% |  | 67.2% | 67.2% |  | 65.7% | 68.6% |  | 66.7% | 66.7% |  |
| Black | 11.6% | 10.1% |  | 11.3% | 11.3% |  | 13.7% | 11.5% |  | 12.9% | 12.9% |  |
| Asian | 2.4% | 2.3% |  | 2.4% | 2.4% |  | 1.9% | 1.8% |  | 1.9% | 1.9% |  |
| Hispanic | 10.4% | 9.8% |  | 10.3% | 10.2% |  | 9.6% | 9.2% |  | 9.4% | 9.4% |  |
| Unknown | 8.9% | 8.8% |  | 8.8% | 8.9% |  | 9.1% | 8.8% |  | 9.0% | 9.1% |  |
| Medicare Advantage enrollment, %col | 67.4% | 68.7% | 0.03 | 67.7% | 67.6% | -0.00 | 64.6% | 66.1% | 0.03 | 65.1% | 65.1% | 0.00 |
| Residence in long-term care or hospitalization on index date, %col | 8.9% | 10.2% | 0.05 | 9.2% | 9.2% | 0.00 | 16.3% | 18.0% | 0.05 | 16.9% | 17.0% | 0.00 |
| **Diseases and frailty in one year prior to index date, %col** | | | | | | | | | | | | |
| Atrial fibrillation | 7.7% | 7.5% | -0.01 | 7.6% | 7.7% | 0.00 | 8.4% | 8.0% | -0.02 | 8.3% | 8.3% | 0.00 |
| SCA/VA | 0.5% | 0.6% | 0.01 | 0.5% | 0.5% | 0.00 | 0.8% | 0.9% | 0.01 | 0.9% | 0.9% | 0.00 |
| Heart failure | 13.7% | 14.9% | 0.03 | 14.0% | 14.2% | 0.01 | 23.7% | 24.7% | 0.02 | 24.2% | 24.4% | 0.00 |
| Hypertension | 66.1% | 65.5% | -0.01 | 66.0% | 66.1% | 0.00 | 68.5% | 68.2% | -0.01 | 68.5% | 68.5% | 0.00 |
| CKD | 20.9% | 18.4% | -0.06 | 20.4% | 20.5% | 0.00 | 23.9% | 20.5% | -0.08 | 22.8% | 22.9% | 0.00 |
| Stroke | 4.9% | 5.1% | 0.01 | 5.0% | 5.1% | 0.00 | 5.0% | 5.4% | 0.01 | 5.2% | 5.2% | 0.00 |
| Cirrhosis | 1.2% | 0.8% | -0.04 | 1.1% | 1.2% | 0.00 | 1.6% | 0.9% | -0.06 | 1.4% | 1.4% | 0.00 |
| Ascites | 1.6% | 1.4% | -0.02 | 1.6% | 1.6% | -0.00 | 2.4% | 1.8% | -0.04 | 2.2% | 2.2% | -0.00 |
| Diabetes insipidus | 0.1% | 0.1% | 0.00 | 0.1% | 0.0% | -0.00 | 0.1% | 0.1% | -0.00 | 0.1% | 0.1% | -0.00 |
| Edema | 25.0% | 26.5% | 0.03 | 25.3% | 25.4% | 0.00 | 25.2% | 25.9% | 0.02 | 25.5% | 25.6% | 0.00 |
| Glaucoma | 8.5% | 8.2% | -0.01 | 8.4% | 8.4% | -0.00 | 7.6% | 7.5% | -0.01 | 7.6% | 7.5% | -0.00 |
| Nocturia | 2.8% | 2.7% | -0.00 | 2.8% | 2.8% | 0.00 | 2.7% | 2.6% | -0.00 | 2.6% | 2.7% | 0.00 |
| Osteoporosis | 5.8% | 6.3% | 0.02 | 5.9% | 5.9% | 0.00 | 4.2% | 4.6% | 0.02 | 4.4% | 4.4% | 0.00 |
| Pulmonary congestion and hypostasis or pulmonary edema | 4.1% | 4.8% | 0.04 | 4.2% | 4.3% | 0.00 | 7.2% | 8.0% | 0.03 | 7.6% | 7.7% | 0.00 |
| Nephrolithiasis | 3.0% | 2.8% | -0.01 | 2.9% | 3.0% | 0.00 | 2.9% | 2.9% | -0.01 | 2.9% | 3.0% | 0.00 |
| Metabolic alkalosis | 0.2% | 0.3% | 0.00 | 0.2% | 0.2% | 0.00 | 0.4% | 0.4% | 0.00 | 0.4% | 0.4% | 0.00 |
| Cushing’s syndrome | 0.1% | 0.1% | 0.05 | 0.1% | 0.1% | 0.00 | 0.1% | 0.1% | 0.00 | 0.1% | 0.1% | 0.00 |
| Hyperaldosteronism | 0.1% | 0.0% | -0.00 | 0.1% | 0.1% | -0.00 | 0.1% | 0.0% | -0.01 | 0.1% | 0.1% | 0.00 |
| Adrenogenital disorders | 0.0% | 0.0% | -0.00 | 0.0% | 0.0% | -0.00 | 0.0% | 0.0% | 0.00 | 0.0% | 0.0% | 0.00 |
| Other corticoadrenal overactivity or ACTH-producing bronchogenic tumor | 0.0% | 0.0% | -0.00 | 0.0% | 0.0% | 0.00 | 0.0% | 0.0% | 0.00 | 0.0% | 0.0% | -0.00 |
| Hyperthyroidism | 1.4% | 1.5% | 0.00 | 1.43% | 1.4% | -0.00 | 1.3% | 1.4% | 0.05 | 1.4% | 1.4% | -0.00 |
| Pyloric stenosis | 0.1% | 0.1% | -0.00 | 0.1% | 0.1% | 0.00 | 0.1% | 0.1% | 0.03 | 0.1% | 0.1% | -0.00 |
| Alcoholism or delirium tremens | 1.7% | 1.6% | -0.01 | 1.7% | 1.7% | 0.00 | 2.4% | 2.1% | -0.02 | 2.3% | 2.3% | 0.00 |
| Leukemia | 0.5% | 0.6% | 0.00 | 0.5% | 0.6% | 0.00 | 0.5% | 0.5% | -0.00 | 0.5% | 0.5% | 0.00 |
| Systemic lupus erythematosus | 0.7% | 0.7% | 0.00 | 0.7% | 0.7% | 0.00 | 0.7% | 0.6% | -0.00 | 0.7% | 0.7% | 0.00 |
| Amyloidosis | 0.1% | 0.1% | -0.01 | 0.1% | 0.1% | 0.00 | 0.1% | 0.1% | -0.00 | 0.1% | 0.1% | -0.00 |
| Corticoadrenal insufficiency | 0.3% | 0.3% | 0.00 | 0.3% | 0.3% | 0.00 | 0.3% | 0.3% | 0.00 | 0.3% | 0.3% | 0.00 |
| Hyperosmolality | 0.7% | 0.7% | 0.00 | 0.7% | 0.7% | -0.00 | 0.8% | 0.8% | -0.00 | 0.8% | 0.8% | 0.00 |
| Acidosis | 1.9% | 1.9% | 0.00 | 1.9% | 1.9% | 0.00 | 2.7% | 2.5% | -0.01 | 2.6% | 2.7% | 0.00 |
| Obstructive uropathy | 0.6% | 0.6% | -0.00 | 0.6% | 0.6% | 0.00 | 0.7% | 0.6% | -0.00 | 0.7% | 0.7% | 0.00 |
| Sickle cell disease | 0.0% | 0.0% | -0.01 | 0.0% | 0.0% | 0.00 | 0.0% | 0.0% | -0.00 | 0.0% | 0.0% | 0.00 |
| HIV/AIDS | 0.2% | 0.2% | -0.01 | 0.2% | 0.2% | -0.00 | 0.2% | 0.2% | -0.01 | 0.2% | 0.2% | -0.00 |
| Renal transplantation | 0.1% | 0.0% | -0.01 | 0.1% | 0.1% | 0.00 | 0.0% | 0.0% | -0.01 | 0.0% | 0.0% | -0.00 |
| Periodic paralysis | 0.0% | 0.0% | -0.00 | 0.0% | 0.0% | 0.00 | 0.0% | 0.0% | -0.00 | 0.0% | 0.0% | 0.00 |
| Disorders of magnesium metabolism | 1.5% | 1.5% | 0.00 | 1.5% | 1.6% | 0.00 | 1.7% | 1.6% | -0.01 | 1.7% | 1.7% | 0.00 |
| Claims-based frailty index, mean (SD) | 0.18 (0.07) | 0.19 (0.07) | 0.05 | 0.19 (0.08) | 0.19 (0.14) | 0.01 | 0.19 (0.07) | 0.19 (0.07) | 0.03 | 0.19 (0.08) | 0.19 (0.11) | 0.00 |
| **Drug markers of diseases in one year prior to index date, %col** | | | | | | | | | | | | |
| ACEI/ARB | 51.8% | 49.3% | -0.05 | 51.3% | 51.3% | 0.00 | 52.3% | 51.1% | -0.03 | 51.9% | 51.9% | -0.00 |
| Aliskiren | 0.2% | 0.2% | -0.00 | 0.2% | 0.2% | -0.00 | 0.3% | 0.3% | -0.00 | 0.3% | 0.3% | 0.00 |
| Potassium-sparing diuretics | 4.7% | 5.7% | 0.04 | 5.0% | 5.0% | 0.00 | 5.2% | 6.8% | 0.07 | 5.8% | 5.8% | 0.00 |
| Aldosterone antagonists | 2.4% | 1.7% | -0.05 | 2.3% | 2.3% | 0.00 | 3.1% | 2.0% | -0.07 | 2.7% | 2.7% | 0.00 |
| Beta-2 agonists | 21.4% | 22.2% | 0.02 | 21.6% | 21.7% | 0.00 | 21.5% | 22.3% | 0.02 | 21.8% | 21.9% | 0.00 |
| Anorexiants/antiobesity agents | 0.1% | 0.1% | -0.00 | 0.1% | 0.1% | -0.00 | 0.1% | 0.1% | -0.00 | 0.1% | 0.1% | 0.00 |
| Antiadrenergic agents | 16.0% | 15.8% | -0.00 | 16.0% | 16.0% | -0.00 | 16.9% | 16.8% | -0.00 | 16.9% | 17.0% | 0.00 |
| Antiarrhythmics, type I, except lidocaine and phenytoin | 0.9% | 1.1% | 0.02 | 1.0% | 1.0% | 0.00 | 0.9% | 1.1% | 0.02 | 1.0% | 1.0% | -0.00 |
| Antiarrhythmics, type III | 3.1% | 3.7% | 0.03 | 3.3% | 3.3% | 0.00 | 3.5% | 4.3% | 0.04 | 3.8% | 3.9% | 0.00 |
| Beta blockers, systemic | 39.9% | 39.7% | -0.00 | 39.8% | 39.8% | -0.00 | 41.0% | 41.2% | 0.00 | 41.1% | 41.0% | -0.00 |
| Calcium channel blockers, dihydropyridines | 25.6% | 24.0% | -0.04 | 25.2% | 25.3% | 0.00 | 24.9% | 23.7% | -0.03 | 24.5% | 24.5% | -0.00 |
| Calcium channel blocker, non-dihydropyridines | 7.2% | 8.0% | 0.03 | 7.4% | 7.5% | 0.00 | 7.6% | 8.6% | 0.04 | 7.9% | 7.9% | -0.00 |
| Antidiabetic agents | 21.3% | 19.7% | -0.04 | 20.9% | 20.9% | -0.00 | 24.5% | 22.9% | -0.04 | 24.0% | 24.0% | 0.00 |
| Insulin | 7.8% | 6.8% | -0.04 | 7.6% | 7.6% | 0.00 | 10.2% | 8.6% | -0.06 | 9.6% | 9.7% | 0.00 |
| Warfarin | 8.2% | 9.4% | 0.04 | 8.5% | 8.5% | 0.00 | 9.1% | 10.4% | 0.04 | 9.6% | 9.6% | 0.00 |
| DOAC | 5.0% | 5.0% | -0.00 | 5.0% | 5.0% | 0.00 | 4.7% | 4.7% | 0.00 | 4.7% | 4.7% | -0.00 |
| Corticosteroids, inhaled | 19.0% | 19.2% | 0.01 | 19.1% | 19.1% | 0.00 | 17.5% | 17.9% | 0.01 | 17.6% | 17.7% | 0.00 |
| Corticosteroids, oral | 26.4% | 27.0% | 0.01 | 26.5% | 26.7% | 0.00 | 24.6% | 25.5% | 0.02 | 25.0% | 25.1% | 0.00 |
| Digoxin, oral | 2.7% | 3.2% | 0.03 | 2.8% | 2.8% | 0.00 | 3.2% | 3.7% | 0.02 | 3.4% | 3.4% | 0.00 |
| Immunosuppressants for organ transplant | 1.6% | 1.5% | -0.01 | 1.6% | 1.6% | -0.00 | 1.4% | 1.2% | -0.01 | 1.3% | 1.3% | 0.00 |
| Lipid-lowering agents | 48.5% | 47.5% | -0.02 | 48.3% | 48.3% | 0.00 | 47.3% | 47.5% | 0.00 | 47.4% | 47.4% | 0.00 |
| Nitrates | 7.0% | 7.3% | 0.01 | 7.0% | 7.1% | 0.00 | 7.7% | 8.1% | 0.02 | 7.8% | 7.8% | -0.00 |
| Vasodilators, non-nitrates | 3.2% | 2.9% | -0.02 | 3.1% | 3.1% | 0.00 | 3.8% | 3.2% | -0.03 | 3.6% | 3.6% | 0.00 |
| Thyroid hormones | 18.9% | 19.7% | 0.02 | 19.1% | 19.1% | -0.00 | 16.1% | 16.8% | 0.02 | 16.3% | 16.3% | 0.00 |
| Xanthine oxidase inhibitors | 4.1% | 3.7% | -0.02 | 4.0% | 4.0% | 0.00 | 4.7% | 4.0% | -0.03 | 4.5% | 4.5% | -0.00 |
| Antiglaucoma agents, ophthalmic | 6.5% | 6.5% | -0.00 | 6.5% | 6.5% | -0.00 | 5.7% | 5.8% | 0.00 | 5.7% | 5.7% | -0.00 |
| Antiglaucoma agents, oral | 0.3% | 0.3% | -0.00 | 0.3% | 0.3% | 0.00 | 0.3% | 0.3% | -0.00 | 0.3% | 0.3% | -0.00 |
| Bone protective drugs | 6.0% | 6.9% | 0.04 | 6.2% | 6.1% | -0.00 | 4.4% | 5.3% | 0.04 | 4.7% | 4.7% | -0.00 |
| **Potassium laboratory tests in 30 days prior to index date, %col** | 31.5% | 30.9% | -0.01 | 31.4% | 31.4% | 0.00 | 29.7% | 29.2% | -0.01 | 29.5% | 29.5% | -0.00 |
| **Hospitalization in 30 days prior to index date, %col** | 16.6% | 18. 9% | 0.06 | 17.1% | 17.3% | 0.00 | 25.6% | 28.4% | 0.06 | 26.6% | 26.8% | 0.00 |
| ACEI/ARB: Angiotensin converting enzyme inhibitor/angiotensin II receptor antagonist; ACTH: adrenocorticotropic hormone; CKD: chronic kidney disease; DOAC: direct-acting oral anticoagulant; HIV/AIDS: human immunodeficiency virus/Acquired immunodeficiency syndrome; K+: potassium; PS: propensity score; SD: standard deviation; SDiff: standardized difference  ^†^ Bolded values indicate meaningful imbalance (\|standardized difference\| >0.1) | | | | | | | | | | | | |
