# Supplementary material for: Impact of empiric potassium supplementation on mortality, sudden cardiac arrest and stroke in furosemide initiators
Source: Br J Clin Pharmacol. 2026 May 3;92(8):2924–36. doi: 10.1002/bcp.70584 (PMC13421057; doi:10.1002/bcp.70584)
Supplement: Supplementary file 14 — Table S7. Baseline characteristics among trimmed individuals (<2.5th percentile or >97.5th percentile) vs. full remaining cohort. [file BCP-92-2924-s012.docx]

**Table S7. Baseline characteristics among trimmed individuals (<2.5^th^ percentile or >97.5^th^ percentile) vs. full remaining cohort**

| **Characteristics** | **Furosemide <40 mg/day** | | | | | | **Furosemide ≥40 mg/day** | | | | | |
| --- | --- | --- | --- | --- | --- | --- | --- | --- | --- | --- | --- | --- |
|  | **<2.5th percentile vs. full** | | | **>97.5th percentile vs. full** | | | **<2.5th percentile vs. full** | | | **>97.5th percentile vs. full** | | |
|  | **Trimmed-out cohort**  **(n=44,557)** | **Full remaining cohort**  **(n=445,778)** | **SDiff^†^** | **Trimmed-out cohort**  **(n=21,127)** | **Full remaining cohort (n=445,778)** | **SDiff^†^** | **Trimmed-out cohort**  **(n=27,524)** | **Full remaining cohort (n=270,766)** | **SDiff^†^** | **Trimmed-out cohort**  **(n=22,413)** | **Full remaining cohort (n=270,766)** | **SDiff^†^** |
| Empiric potassium, n (% col) | 6.2% | 21.6% | **-0.46** | 52.5% | 21.6% | **0.68** | 10.3% | 34.4% | **-0.61** | 15.6% | 34.4% | **0.94** |
| Propensity score, mean (SD) | 0.07 (0.02) | 0.21 (0.08) | **-2.68** | 0.54 (0.10) | 0.21 (0.08) | **2.56** | 0.11 (0.03) | 0.34 (0.11) | **-2.68** | 0.77 (0.09) | 0.34 (0.11) | **2.64** |
| **Demographic** | | | | | | | | | | | | |
| Age groups, % col |  |  | **0.20** |  |  | **0.22** |  |  | **0.20** |  |  | **0.40** |
| <35 years | 5.0% | 2.2% |  | 0.8% | 2.2% |  | 4.9% | 1.9% |  | 0.7% | 1.9% |  |
| 35-44 years | 4.1% | 5.0% |  | 2.3% | 5.0% |  | 3.6% | 4.8% |  | 2.3% | 4.8% |  |
| 45-54 years | 7.9% | 9.8% |  | 8.0% | 9.8% |  | 8.8% | 10.6% |  | 9.9% | 10.6% |  |
| 55-64 years | 13.4% | 14.9% |  | 17.3% | 14.9% |  | 18.3% | 17.3% |  | 23.7% | 17.3% |  |
| 65-74 years | 27.8% | 25.3% |  | 28.3% | 25.3% |  | 30.0% | 27.1% |  | 35.1% | 27.1% |  |
| 75-84 years | 28.0% | 29.6% |  | 32.7% | 29.6% |  | 24.9% | 27.9% |  | 25.0% | 27.9% |  |
| 85+ years | 13.8% | 13.3% |  | 10.6% | 13.3% |  | 9.5% | 10.5% |  | 3.3% | 10.5% |  |
| Female sex, %col | 56.9% | 61.7% | -0.10 | 45.4% | 61.7% | **-0.33** | 43.7% | 51.8% | **-0.16** | 30.2% | 51.8% | **-0.45** |
| Race, %col |  |  | **0.15** |  |  | **0.34** |  |  | **0.21** |  |  | **0.29** |
| White | 64.2% | 67.2% |  | 79.5% | 67.2% |  | 57.6% | 66.7% |  | 77.5% | 66.7% |  |
| Black | 13.0% | 11.3% |  | 4.3% | 11.3% |  | 19.5% | 12.9% |  | 6.4% | 12.9% |  |
| Asian | 3.64% | 2.4% |  | 2.7% | 2.4% |  | 3.1% | 1.9% |  | 1.9% | 1.9% |  |
| Hispanic | 11.3% | 10.3% |  | 6.3% | 10.3% |  | 11.2% | 9.4% |  | 6.0% | 9.4% |  |
| Unknown | 7.9% | 8.9% |  | 7.3% | 8.9% |  | 8.7% | 9.0% |  | 8.2% | 9.0% |  |
| Medicare Advantage enrollment, %col | 70.2% | 67.7% | 0.05 | 67.2% | 67.7% | -0.01 | 68.1% | 65.1% | 0.06 | 57.5% | 65.1% | **-0.16** |
| Residence in long-term care or hospitalization on index date, %col | 5.4% | 9.2% | **-0.15** | 55.0% | 9.2% | **1.13** | 15.1% | 16.9% | -0.05 | 74.7% | 16.9% | **1.43** |
| **Diseases and frailty in one year prior to index date, %col** | | | | | | | | | | | | |
| Atrial fibrillation | 8.4% | 7.6% | 0.03 | 11.4% | 7.6% | **0.13** | 9.6% | 8.3% | 0.05 | 14.6% | 8.3% | **0.20** |
| SCA/VA | 0.5% | 0.5% | 0.00 | 1.8% | 0.5% | **0.12** | 1.1% | 0.9% | 0.02 | 2.3% | 0.9% | **0.11** |
| Heart failure | 15.2% | 14.0% | 0.03 | 31.6% | 14.0% | **0.43** | 30.8% | 24.1% | **0.15** | 33.4% | 24.1% | **0.21** |
| Hypertension | 73.5% | 66.0% | **0.16** | 77.2% | 66.0% | **0.25** | 80.7% | 68.4% | **0.28** | 85.7% | 68.4% | **0.42** |
| CKD | 38.0% | 20.4% | **0.40** | 18.7% | 20.4% | -0.04 | 59.0% | 22.7% | **0.79** | 24.3% | 22.7% | 0.04 |
| Stroke | 5.8% | 5.0% | 0.04 | 7.6% | 5.0% | **0.11** | 15.9% | 5.2% | 0.03 | 7.5% | 5.2% | 0.10 |
| Cirrhosis | 5.7% | 1.1% | **0.25** | 0.2% | 1.1% | **-0.12** | 13.1% | 1.4% | **0.46** | 0.3% | 1.4% | **-0.12** |
| Ascites | 4.6% | 1.6% | **0.18** | 1.0% | 1.6% | -0.06 | 12.5% | 2.2% | **0.40** | 0.4% | 2.2% | **-0.16** |
| Diabetes insipidus | 0.1% | 0.1% | 0.01 | 0.0% | 0.1% | -0.01 | 0.1% | 0.1% | 0.03 | 0.0% | 0.1% | -0.01 |
| Edema | 20.8% | 25.3% | **-0.11** | 29.6% | 25.3% | **0.10** | 28.0% | 25.4% | 0.06 | 21.3% | 25.4% | -0.10 |
| Glaucoma | 11.0% | 8.4% | 0.09 | 6.8% | 8.4% | -0.06 | 9.6% | 7.6% | 0.07 | 7.0% | 7.6% | -0.02 |
| Nocturia | 3.3% | 2.8% | 0.03 | 2.9% | 2.8% | 0.01 | 3.4% | 2.6% | 0.04 | 3.1% | 2.6% | 0.03 |
| Osteoporosis | 4.8% | 5.9% | -0.05 | 7.4% | 5.9% | 0.06 | 3.5% | 4.4% | -0.04 | 3.9% | 4.4% | -0.02 |
| Pulmonary congestion and hypostasis or pulmonary edema | 3.6% | 4.2% | -0.03 | 17.2% | 4.2% | **0.43** | 8.3% | 7.5% | 0.03 | 21.3% | 7.5% | **0.40** |
| Nephrolithiasis | 3.8% | 2.9% | 0.05 | 3.1% | 2.9% | 0.01 | 4.8% | 2.9% | 0.10 | 3.5% | 2.9% | 0.03 |
| Metabolic alkalosis | 0.3% | 0.2% | 0.02 | 0.7% | 0.2% | 0.07 | 0.7% | 0.4% | 0.04 | 0.8% | 0.4% | 0.04 |
| Cushing’s syndrome | 0.0% | 0.1% | -0.01 | 0.2% | 0.1% | 0.03 | 0.1% | 0.1% | -0.01 | 0.1% | 0.1% | -0.01 |
| Hyperaldosteronism | 0.1% | 0.1% | 0.03 | 0.0% | 0.1% | -0.01 | 0.3% | 0.1% | 0.05 | 0.0% | 0.1% | -0.02 |
| Adrenogenital disorders | 0.0% | 0.0% | -0.00 | 0.0% | 0.0% | 0.00 | 0.0% | 0.0% | -0.01 | 0.0% | 0.0% | 0.00 |
| Other corticoadrenal overactivity or ACTH-producing bronchogenic tumor | 0.0% | 0.0% | -0.01 | 0.0% | 0.0% | 0.01 | 0.0% | 0.0% | 0.00 | 0.0% | 0.0% | -0.01 |
| Hyperthyroidism | 1.7% | 1.4% | 0.02 | 1.6% | 1.4% | 0.01 | 1.5% | 1.4% | 0.01 | 1.4% | 1.4% | 0.00 |
| Pyloric stenosis | 0.1% | 0.1% | 0.00 | 0.1% | 0.1% | 0.01 | 0.1% | 0.1% | 0.01 | 0.0% | 0.1% | -0.02 |
| Alcoholism or delirium tremens | 3.2% | 1.7% | 0.10 | 2.4% | 1.7% | 0.05 | 7.2% | 2.3% | **0.23** | 2.7% | 2.3% | 0.03 |
| Leukemia | 0.5% | 0.5% | -0.00 | 0.8% | 0.5% | 0.04 | 0.6% | 0.5% | 0.01 | 0.5% | 0.5% | -0.00 |
| Systemic lupus erythematosus | 0.7% | 0.7% | 0.01 | 0.7% | 0.7% | -0.00 | 1.0% | 0.7% | 0.04 | 0.3% | 0.7% | -0.05 |
| Amyloidosis | 0.3% | 0.1% | 0.04 | 0.0% | 0.1% | -0.02 | 0.3% | 0.1% | 0.05 | 0.0% | 0.1% | -0.02 |
| Corticoadrenal insufficiency | 0.3% | 0.3% | 0.00 | 0.4% | 0.3% | 0.02 | 0.4% | 0.3% | 0.01 | 0.0% | 0.3% | 0.01 |
| Hyperosmolality | 0.9% | 0.7% | 0.03 | 1.6% | 0.7% | 0.08 | 1.5% | 0.8% | 0.06 | 1.5% | 0.8% | 0.07 |
| Acidosis | 3.2% | 1.9% | 0.08 | 4.3% | 1.9% | **0.14** | 8.0% | 2.6% | **0.24** | 4.9% | 2.6% | **0.12** |
| Obstructive uropathy | 0.8% | 0.6% | 0.02 | 0.7% | 0.6% | 0.01 | 1.4% | 0.7% | 0.08 | 0.6% | 0.7% | -0.01 |
| Sickle cell disease | 0.1% | 0.0% | 0.02 | 0.0% | 0.0% | -0.01 | 0.1% | 0.0% | 0.02 | 0.0% | 0.0% | 0.00 |
| HIV/AIDS | 0.4% | 0.2% | 0.03 | 0.2% | 0.2% | 0.00 | 0.7% | 0.2% | 0.07 | 0.2% | 0.2% | -0.01 |
| Renal transplantation | 1.5% | 0.1% | **0.17** | 0.0% | 0.1% | -0.02 | 3.2% | 0.0% | **0.25** | 0.0% | 0.0% | -0.02 |
| Periodic paralysis | 0.0% | 0.0% | 0.01 | 0.0% | 0.0% | -0.01 | 0.0% | 0.0%) | 0.02 | 0.0% | 0.0% | -0.00 |
| Disorders of magnesium metabolism | 1.9% | 1.5% | 0.03 | 2.9% | 1.5% | 0.09 | 3.7% | 1.7% | **0.13** | 2.3% | 1.7% | 0.05 |
| Claims-based frailty index, mean (SD) | 0.18 (0.07) | 0.19 (0.07) | -0.01 | 0.20 (0.07) | 0.19 (0.07) | **0.32** | 0.20 (0.07) | 0.19 (0.07) | **0.17** | 0.19 (0.05) | 0.19 (0.07) | 0.04 |
| **Drug markers of diseases in one year prior to index date, %col** | | | | | | | | | | | | |
| ACEI/ARB | 61.9% | 51.3% | **0.22** | 44.4% | 51.3% | **-0.14** | 59.5% | 51.9% | **0.15** | 54.8% | 51.9% | 0.06 |
| Aliskiren | 0.3% | 0.2% | 0.01 | 0.1% | 0.2% | -0.04 | 0.4% | 0.3% | 0.01 | 0.2% | 0.3% | -0.03 |
| Potassium-sparing diuretics | 1.9% | 5.0% | **-0.17** | 6.7% | 5.0% | 0.07 | 1.8% | 5.8% | **-0.21** | 5.1% | 5.8% | -0.03 |
| Aldosterone antagonists | 10.7% | 2.3% | **0.35** | 1.0% | 2.3% | **-0.15** | 15.9% | 2.7% | **0.47** | 0.7% | 2.7% | **-0.15** |
| Beta-2 agonists | 19.8% | 21.6% | -0.04 | 19.8% | 21.6% | -0.04 | 18.5% | 21.8% | -0.08 | 14.6% | 21.8% | **-0.19** |
| Anorexiants/antiobesity agents | 0.2% | 0.1% | 0.02 | 0.0% | 0.1% | -0.03 | 0.1% | 0.1% | -0.00 | 0.0% | 0.1% | -0.03 |
| Antiadrenergic agents | 16.6% | 16.0% | 0.02 | 14.2% | 16.0% | -0.05 | 21.1% | 16.9% | **0.11** | 14.9% | 16.9% | -0.06 |
| Antiarrhythmics, type I, except lidocaine and phenytoin | 0.5% | 1.0% | -0.06 | 2.3% | 1.0% | **0.11** | 0.3% | 1.0% | -0.09 | 1.5% | 1.0% | 0.04 |
| Antiarrhythmics, type III | 2.6% | 3.2% | -0.04 | 8.8% | 3.2% | **0.23** | 2.8% | 3.8% | -0.06 | 9.9% | 3.8% | **0.24** |
| Beta blockers, systemic | 47.9% | 39.8% | **0.16** | 48.2% | 39.8% | **0.17** | 52.0% | 41.0% | **0.22** | 54.6% | 41.0% | **0.27** |
| Calcium channel blockers, dihydropyridines | 32.8% | 25.2% | **0.17** | 18.1% | 25.2% | **-0.17** | 37.4% | 24.5% | **0.28** | 19.8% | 24.5% | **-0.11** |
| Calcium channel blocker, non-dihydropyridines | 6.2% | 7.4% | -0.05 | 9.6% | 7.4% | 0.08 | 6.2% | 7.9% | -0.07 | 7.8% | 7.9% | -0.01 |
| Antidiabetic agents | 27.1% | 20.9% | **0.14** | 17.6% | 20.9% | -0.08 | 31.0% | 23.9% | **0.16** | 23.9% | 23.9% | -0.00 |
| Insulin | 13.8% | 7.6% | **0.20** | 5.4% | 7.6% | -0.09 | 22.1% | 9.6% | **0.34** | 8.0% | 9.6% | -0.06 |
| Warfarin | 7.1% | 8.5% | -0.05 | 14.5% | 8.5% | **0.19** | 7.6% | 9.5% | -0.07 | 10.2% | 9.5% | 0.02 |
| DOAC | 4.8% | 5.0% | -0.01 | 4.4% | 5.0% | -0.03 | 4.3% | 4.7% | -0.02 | 3.8% | 4.7% | -0.04 |
| Corticosteroids, inhaled | 18.3% | 19.1% | -0.02 | 17.1% | 19.1% | -0.05 | 15.6% | 17.6% | -0.05 | 14.5% | 17.6% | -0.09 |
| Corticosteroids, oral | 22.1% | 26.5% | -0.10 | 24.5% | 26.5% | -0.05 | 21.3% | 24.9% | -0.09 | 20.0% | 24.9% | **-0.12** |
| Digoxin, oral | 2.1% | 2.8% | 0.05 | 4.7% | 2.8% | **0.10** | 2.6% | 3.4% | -0.05 | 3.1% | 3.4% | -0.02 |
| Immunosuppressants for organ transplant | 3.3% | 1.6% | **0.11** | 1.2% | 1.6% | -0.03 | 4.9% | 1.3% | **0.21** | 0.7% | 1.3% | -0.07 |
| Lipid-lowering agents | 55.3% | 48.2% | **0.14** | 54.7% | 48.2% | **0.13** | 52.5% | 47.4% | **0.10** | 64.8% | 47.4% | **0.36** |
| Nitrates | 7.2% | 7.0% | 0.00 | 16.1% | 7.0% | **0.29** | 9.0% | 7.8% | 0.04 | 22.5% | 7.8% | **0.42** |
| Vasodilators, non-nitrates | 4.7% | 3.1% | 0.08 | 1.9% | 3.1% | -0.08 | 10.0% | 3.6% | **0.26** | 2.0% | 3.6% | **-0.10** |
| Thyroid hormones | 16.0% | 19.1% | -0.08 | 17.5% | 19.1% | -0.04 | 14.3% | 16.3% | -0.06 | 13.1% | 16.3% | -0.09 |
| Xanthine oxidase inhibitors | 7.3% | 4.0% | **0.14** | 3.4% | 4.0% | -0.03 | 10.6% | 4.5% | **0.23** | 4.0% | 4.5% | -0.02 |
| Antiglaucoma agents, ophthalmic | 7.5% | 6.5% | 0.04 | 5.3% | 6.5% | -0.05 | 6.8% | 5.7% | 0.04 | 4.4% | 5.7% | -0.06 |
| Antiglaucoma agents, oral | 0.3% | 0.3% | 0.01 | 0.2% | 0.3% | -0.03 | 0.3% | 0.3% | 0.00 | 0.2% | 0.3% | -0.03 |
| Bone protective drugs | 4.1% | 6.2% | -0.09 | 7.1% | 6.2% | 0.04 | 2.7% | 4.7% | **-0.11** | 4.0% | 4.7% | -0.04 |
| **Potassium laboratory tests in 30 days prior to index date, %col** | 36.5% | 31.4% | **0.11** | 42.1% | 31.4% | **0.22** | 44.3% | 29.5% | **0.31** | 48.0% | 29.5% | **0.38** |
| **Hospitalization in 30 days prior to index date, %col** | 13.1% | 17.1% | **-0.11** | 71.7% | 17.1% | **1.31** | 28.0% | 26.6% | 0.03 | 93.4% | 26.6% | **1.87** |
| ACEI/ARB: Angiotensin converting enzyme inhibitor/angiotensin II receptor antagonist; ACTH: adrenocorticotropic hormone; CKD: chronic kidney disease; DOAC: direct-acting oral anticoagulant; HIV/AIDS: human immunodeficiency virus/Acquired immunodeficiency syndrome; K+: potassium; PS: propensity score; SD: standard deviation; SDiff: standardized difference  ^†^ Bolded values indicate meaningful imbalance (\|standardized difference\| >0.1) | | | | | | | | | | | | |
