# Supplementary material for: Impact of empiric potassium supplementation on mortality, sudden cardiac arrest and stroke in furosemide initiators
Source: Br J Clin Pharmacol. 2026 May 3;92(8):2924–36. doi: 10.1002/bcp.70584 (PMC13421057; doi:10.1002/bcp.70584)
Supplement: Supplementary file 15 — Table S8. IPTW Cox proportional hazards models for all outcomes among individuals initiating furosemide <40 mg/day or ≥40 mg/day with vs. without empiric potassium at six‐month and one‐year follow‐up†. [file BCP-92-2924-s016.docx]

**Table S8. IPTW Cox proportional hazards models for all outcomes among individuals initiating furosemide <40 mg/day or ≥40 mg/day with vs. without empiric potassium at six-month and one-year follow-up^†^**

| **Outcome** | **Analysis** | | **Furosemide <40 mg/day^‡^** | | **Furosemide ≥40 mg/day^‡^** | |
| --- | --- | --- | --- | --- | --- | --- |
|  |  |  | **Crude HR (95%CI)** | **IPTW-HR (95%CI)** | **Crude HR (95%CI)** | **IPTW-HR (95%CI)** |
| All-cause mortality | Six-month follow-up | As-started analysis | 1.07 (1.04-1.10) | 1.00 (0.97-1.03) | 0.89 (0.86-0.92) | 0.97 (0.94-1.00)^1^ |
|  |  | As-treated analysis | 1.08 (1.03-1.14) | 0.98 (0.93-1.04) | 0.95 (0.90-1.00)^2^ | 0.98 (0.92-1.03) |
|  | One-year follow-up | As-started analysis | 1.07 (1.04-1.09) | 1.02 (0.99-1.04) | 0.90 (0.88-0.92) | 0.98 (0.95-1.01) |
|  |  | As-treated analysis | 1.08 (1.03-1.13) | 0.98 (0.93-1.04) | 0.95 (0.90-0.99) | 0.97 (0.92-1.03) |
| SCA/VA | Six-month follow-up | As-started analysis | 1.04 (0.96-1.12) | 1.01 (0.92-1.11) | 0.91 (0.85-0.98) | 0.96 (0.88-1.04) |
|  |  | As-treated analysis | 1.08 (0.97-1.19) | 1.05 (0.92-1.19) | 0.96 (0.88-1.05) | 0.97 (0.87-1.08) |
|  | One-year follow-up | As-started analysis | 1.01 (0.95-1.08) | 1.01 (0.94-1.09) | 0.87 (0.82-0.92) | 0.93 (0.88-1.00)^3^ |
|  |  | As-treated analysis | 1.07 (0.97-1.18) | 1.05 (0.93-1.19) | 0.95 (0.87-1.04) | 0.96 (0.87-1.06) |
| Stroke | Six-month follow-up | As-started analysis | 1.04 (0.99-1.10) | 1.02 (0.96-1.08) | 1.04 (0.98-1.09) | 1.05 (0.991.12) |
|  |  | As-treated analysis | 1.07 (0.99-1.15) | 1.02 (0.94-1.12) | 1.13 (1.05-1.22) | 1.07 (0.99-1.16) |
|  | One-year follow-up | As-started analysis | 1.03 (0.99-1.07) | 1.02 (0.97-1.07) | 0.98 (0.94-1.03) | 1.02 (0.97-1.07) |
|  |  | As-treated analysis | 1.06 (0.99-1.13) | 1.06 (0.99-1.13) | 1.11 (1.04-1.19) | 1.07 (0.98-1.15) |
| CI: confidence interval; HR: hazard ratio; IPTW: inverse probability treatment weighting; SCA/VA: sudden cardiac arrest/ventricular arrhythmia  ^†^ Post-hoc analyses  ^‡^ Sample sizes n=511,532 for full furosemide <40 mg/day cohort and n=320,703 for full furosemide ≥40 mg/day cohort  1. p = 0.0663  2. p = 0.0397  3. p = 0.0522 | | | | | | |
