# Supplementary material for: Impact of empiric potassium supplementation on mortality, sudden cardiac arrest and stroke in furosemide initiators
Source: Br J Clin Pharmacol. 2026 May 3;92(8):2924–36. doi: 10.1002/bcp.70584 (PMC13421057; doi:10.1002/bcp.70584)
Supplement: Supplementary file 16 — Data S1. Supporting Information. [file BCP-92-2924-s006.docx]

**SUPPLEMENTAL METHODS**

**Data source**

Optum’s de-identified Clinformatics^®^ Data Mart (CDM) included sociodemographic information (e.g., age, sex, race/ethnicity, etc.), medical encounters (e.g., inpatient, emergency department [ED] visits), and pharmacy prescription dispensing claims, among others. CDM is representative of the commercially insured population in the U.S.,^1^ allowing for a large-scale and diverse study of chronic medication use.

**High-dimensional propensity score (hdPS) algorithm**^2-4^

Baseline covariates among 17 data dimensions (e.g., diagnostic, procedural, laboratory, and dispensed medications) were included in the algorithm, and up to 200 most prevalent covariates from each dimension were considered. Covariates were ranked based on their potential for bias using the Bross formula and up to the top 500 empiric covariates^2-4^ across dimensions were used to estimate PSs (**Table S1**). In addition to high-dimensional propensity score (hdPS)-identified covariates, the PS model also included investigator-identified baseline covariates in the following categories: 1) demographics: age, sex, race, state of residence, calendar year of cohort entry, Medicare Advantage enrollment, long-term care residence, and hospitalization as of the index date; 2) diseases (e.g., atrial fibrillation, hypertension) and claims-based frailty index (CFI);^5^ and 3) drug markers of chronic diseases (e.g., antidiabetes agents, antihypertensive agents); 4) presence of a potassium laboratory test measured within 30 days prior to index; 5) hospital admission within 30 days prior to index; and 6) measures of intensity of healthcare utilization (**Table S2**).

**Ascertainment of outcomes**

The SCA/VA outcome was identified via ED or inpatient discharge claims having one of the International Classification of Diseases, 9^th^ edition, Clinical Modification (ICD-9-CM) diagnosis codes (positive predictive value [PPV] of 85%)^6^ or forward-mapped ICD-10-CM diagnosis codes^7, 8^ in a first-listed or principal position (**Table S3**). Similarly, the composite stroke outcome was identified via ED or inpatient discharge claims having one of the ICD-9-CM or ICD-10-CM diagnosis codes (PPVs: 82%-91% for ischemic stroke; 89-93% for hemorrhagic stroke)^9-12^ in a first-listed or principal position (**Table S3**).

**Statistical analyses**

***Sensitivity analyses***

We conducted a prespecified sensitivity analysis in which we trimmed extremes from the PS distribution. Since low PS subjects dispensed empiric potassium and high PS subjects not dispensed empiric potassium would have large weights causing instability, we also performed analyses limiting to people retained after trimming of subjects on empiric potassium whose PS were above the 97.5^th^ percentile from the distribution among subjects without empiric potassium, and of subjects without empiric potassium whose PS were below the 2.5^th^ percentile from the distribution of subjects on empiric potassium.^13-16^ Similar Cox proportional hazards models were performed for secondary as-treated analyses.

***Subgroup analyses***

We conducted pre-specified subgroup analyses to examine potential effect modification by (1) age: <35, 35-44, 45-54, 55-64, 65-74, 75-84, 85+ years; (2) history of comorbidity in the year prior to furosemide course: atrial fibrillation, SCA/VA, heart failure, hypertension, chronic kidney disease (CKD), stroke, and cirrhosis; (3) oral medication use as of index date: digoxin, angiotensin-converting enzyme inhibitors/angiotensin II receptor antagonists/aliskiren, other potassium-sparing diuretics or aldosterone antagonists; and (4) initial median empiric potassium dose in each furosemide dose cohort (≤10 vs. >10 mEq/day for the <40 mg/day furosemide cohort, and ≤20 vs. >20 mEq/day for the ≥40 mg/day cohort).

***Post-hoc analyses***

We performed post-hoc analyses restricted to six months and one year of follow-up for both the “as-started” and “as-treated” analyses to limit potential time-varying confounding and competing risks associated with longer follow-up periods.

***Instrumental variable (IV) analysis***

Among individuals with prescribers’ data, we performed IV analyses to examine the effect of empiric potassium use on the outcomes of interest, with physician’s preference as the instrument. This statistical approach can be used to obtain a treatment effect estimation in the presence of unmeasured confounders.^17-20^ An instrument is a variable deemed to be associated with the treatment (i.e., empiric potassium supplementation), but unrelated to the confounders, and unrelated to the outcome other than through its association with the actual treatment.^17-20^ We identified the prescriber for the initial furosemide dispensing and the index date for each specific person in the dataset using the National Provider Identifier (NPI), then classified that prescriber’s prescribing preference as “empiric potassium preferred” vs. “empiric potassium *not* preferred” based on whether an empiric potassium dispensing was accompanying their most recent furosemide dispensing that was before the index date for another person in the dataset. We then estimated the risk difference for each outcome of interest due to use of empiric potassium vs. no use, adjusting for aforementioned pre-defined demographic, disease, drug use, laboratory, and hospitalization baseline covariates. We also performed tests to assess IV assumptions and IV strength.^21^ We assessed the strength of the IV in two ways—by calculating the proportion of compliers via estimating the difference of the empiric potassium supplementation assignment rate among subjects across the values of the IV, and by calculating the F-statistic with 1 degree of freedom for the IV in a regression model that included empiric potassium as dependent variable and the IV and measured demographic, disease, drug use, laboratory, and hospitalization baseline covariates as independent variables.^20^ We also assessed the independence of the IV and unmeasured confounders by measuring imbalances between the measured demographic, disease, drug use, laboratory, and hospitalization baseline covariates among patients with different treatments (i.e., with and without empiric potassium supplementation) and physician’s preference IVs; we then regressed each imbalanced covariate across IV groups on the IV and other covariates^20^ to evaluate whether there were measured and unmeasured confounders that were associated with the IV.

**REFERENCES**

**1.** OptumTM. Real world health care experiences from over 150 million unique individuals since 1993. Available at: <https://www.optum.com/content/dam/optum/resources/productSheets/5302_Data_Assets_Chart_Sheet_ISPOR.pdf>.

**2.** Rassen JA, Blin P, Kloss S, et al. High-dimensional propensity scores for empirical covariate selection in secondary database studies: Planning, implementation, and reporting. *Pharmacoepidemiol Drug Saf.* 2023;32(2): 93-106.

**3.** Rassen JA, Glynn RJ, Brookhart MA, Schneeweiss S. Covariate selection in high-dimensional propensity score analyses of treatment effects in small samples. *Am J Epidemiol.* 2011;173(12): 1404-1413.

**4.** Schneeweiss S, Eddings W, Glynn RJ, Patorno E, Rassen J, Franklin JM. Variable Selection for Confounding Adjustment in High-dimensional Covariate Spaces When Analyzing Healthcare Databases. *Epidemiology.* 2017;28(2): 237-248.

**5.** Kim DH, Schneeweiss S, Glynn RJ, Lipsitz LA, Rockwood K, Avorn J. Measuring Frailty in Medicare Data: Development and Validation of a Claims-Based Frailty Index. *J Gerontol A Biol Sci Med Sci.* 2018;73(7): 980-987.

**6.** Hennessy S, Leonard CE, Freeman CP, et al. Validation of diagnostic codes for outpatient-originating sudden cardiac death and ventricular arrhythmia in Medicaid and Medicare claims data. *Pharmacoepidemiol Drug Saf.* 2010;19(6): 555-562.

**7.** Centers for Medicare and Medicaid Services. General Equivalence Mappings. Available at: <https://www.cms.gov/Medicare/Coding/ICD10/downloads/ICD-10_GEM_fact_sheet.pdf>.

**8.** Fung KW, Richesson R, Smerek M, et al. Preparing for the ICD-10-CM Transition: Automated Methods for Translating ICD Codes in Clinical Phenotype Definitions. *EGEMS (Wash DC).* 2016;4(1): 1211.

**9.** McCormick N, Bhole V, Lacaille D, Avina-Zubieta JA. Validity of Diagnostic Codes for Acute Stroke in Administrative Databases: A Systematic Review. *PLoS One.* 2015;10(8): e0135834.

**10.** Chronic Conditions Data Warehouse. Condition Categories. *Centers for Medicare & Medicaid Services*. Available at: <https://www.ccwdata.org/web/guest/condition-categories>. Accessed 2019, June 10.

**11.** Kumamaru H, Judd SE, Curtis JR, et al. Validity of claims-based stroke algorithms in contemporary Medicare data: reasons for geographic and racial differences in stroke (REGARDS) study linked with medicare claims. *Circ Cardiovasc Qual Outcomes.* 2014;7(4): 611-619.

**12.** Hsieh CY, Chen CH, Li CY, Lai ML. Validating the diagnosis of acute ischemic stroke in a National Health Insurance claims database. *J Formos Med Assoc.* 2015;114(3): 254-259.

**13.** Austin PC, Stuart EA. Moving towards best practice when using inverse probability of treatment weighting (IPTW) using the propensity score to estimate causal treatment effects in observational studies. *Stat Med.* 2015;34(28): 3661-3679.

**14.** Conover MM, Rothman KJ, Sturmer T, Ellis AR, Poole C, Jonsson Funk M. Propensity score trimming mitigates bias due to covariate measurement error in inverse probability of treatment weighted analyses: A plasmode simulation. *Stat Med.* 2021;40(9): 2101-2112.

**15.** Sturmer T, Rothman KJ, Avorn J, Glynn RJ. Treatment effects in the presence of unmeasured confounding: dealing with observations in the tails of the propensity score distribution--a simulation study. *Am J Epidemiol.* 2010;172(7): 843-854.

**16.** Sturmer T, Webster-Clark M, Lund JL, et al. Propensity Score Weighting and Trimming Strategies for Reducing Variance and Bias of Treatment Effect Estimates: A Simulation Study. *Am J Epidemiol.* 2021;190(8): 1659-1670.

**17.** Brookhart MA, Rassen JA, Schneeweiss S. Instrumental variable methods in comparative safety and effectiveness research. *Pharmacoepidemiol Drug Saf.* 2010;19(6): 537-554.

**18.** Brookhart MA, Schneeweiss S. Preference-based instrumental variable methods for the estimation of treatment effects: assessing validity and interpreting results. *Int J Biostat.* 2007;3(1): Article 14.

**19.** Rassen JA, Schneeweiss S, Glynn RJ, Mittleman MA, Brookhart MA. Instrumental variable analysis for estimation of treatment effects with dichotomous outcomes. *Am J Epidemiol.* 2009;169(3): 273-284.

**20.** Ertefaie A, Small DS, Flory JH, Hennessy S. A tutorial on the use of instrumental variables in pharmacoepidemiology. *Pharmacoepidemiol Drug Saf.* 2017;26(4): 357-367.

**21.** Ahmed A, Zannad F, Love TE, et al. A propensity-matched study of the association of low serum potassium levels and mortality in chronic heart failure. *Eur Heart J.* 2007;28(11): 1334-1343.
