# Supplementary material for: Impact of empiric potassium supplementation on mortality, sudden cardiac arrest and stroke in furosemide initiators
Source: Br J Clin Pharmacol. 2026 May 3;92(8):2924–36. doi: 10.1002/bcp.70584 (PMC13421057; doi:10.1002/bcp.70584)
Supplement: Supplementary file 17 — Data S2. Supporting Information. [file BCP-92-2924-s010.docx]

**SUPPLEMENTAL RESULTS**

**Results from the inverse probability of treatment weighting (IPTW) for the secondary outcomes**

***Sudden cardiac arrest/Ventricular arrhythmia (SCA/VA)***

Crude SCA/VA rates were 9.3 (95% CI 9.1–9.5) and 13.6 (13.3–13.8) per 1,000 person-years in those on <40 and ≥40 mg/day furosemide, respectively. As-started IPTW-adjusted Kaplan-Meier curves (**Figures S4-S5**) showed similar SCA/VA-free probability between people with and without empiric potassium throughout follow-up. Visual inspection of Kaplan-Meier curves did not suggest a departure from the proportional-hazards assumption. As-started IPTW-HRs for empiric potassium (vs. no potassium) were similar in both furosemide cohorts, i.e., 0.99 (0.94–1.04) for <40 mg/day and 0.98 (0.94–1.03) for ≥40 mg/day. Findings from the prespecified sensitivity analyses that trimmed the PS distribution were nearly identical (**Table 2**). HRs from the as-treated analyses were nearly identical to and had 95% CIs that overlapped the primary findings above (**Table 2**).

***Stroke***

Crude stroke rates were 25.9 (25.6–26.2) and 27.7 (27.4–28.1) per 1,000 person-years in those on <40 and ≥40 mg/day furosemide, respectively. As-started IPTW-adjusted Kaplan-Meier curves (**Figures S4-S5**) showed similar stroke-free probability between people with and without empiric potassium throughout follow-up. Visual inspection of Kaplan-Meier curves suggested no violation of the proportional-hazards assumption. As-started IPTW-HRs for empiric potassium (vs. no potassium) were similar in both furosemide cohorts, i.e., 1.03 (1.00–1.06, p-value = 0.0354) for <40 mg/day and 1.01 (0.98–1.04) for ≥40 mg/day. HRs from the prespecified sensitivity and secondary analyses were nearly identical to and had 95% CIs that overlapped the primary findings above (**Table 2**).

**Results from the IPTW subgroup analyses (Figures S6-S7)**

***Primary outcome: All-cause mortality***

We noted a statistically significant difference in the association between empiric potassium use and all-cause mortality in both furosemide dose cohorts within the subgroup of people with vs. without a history of congestive heart failure (p-values for difference 0.0001 in the <40 mg/day furosemide cohort and 0.0004 in the ≥40 mg/day furosemide cohort). We also observed statistically significant differences in the association between empiric potassium use and all-cause mortality in the <40 mg/day furosemide cohort within the subgroups of people with vs. without history of atrial fibrillation (p-value for difference = 0.0373) and with vs. without history of hypertension (p-value for difference = 0.031). In the ≥40 mg/day furosemide cohort, there were also statistically significant differences in the association between empiric potassium use and all-cause mortality within different age groups (p-value for difference = 0.0124), within subgroups of people with vs. without history of stroke p-value for difference = 0.0431) and having empiric potassium dose >20 mEq/day (yes vs. no; p-value for difference 0.0001).

***Secondary outcomes***

***SCA/VA***

We observed a statistically significant difference in the association between empiric potassium use and SCA/VA only in the ≥40 mg/day furosemide cohort across subgroups with vs. without history of congestive heart failure (p-value for difference = 0.0013), with vs. without history of hypertension (p-value for difference of p=0.002), with vs. without history of stroke (p-value for difference = 0.0334), and having empiric potassium dose >20 mEq/day (yes vs. no; p-value for difference = 0.0002).

***Stroke***

We observed a statistically significant difference by age groups in the association between empiric potassium use and stroke in both furosemide dose cohorts (p-values for difference of 0.0127 in the <40 mg/day and 0.0139 in the ≥40 mg/day furosemide cohorts, respectively). Additionally, there were also statistically significant differences in the association between empiric potassium use and stroke within the subgroups of people with vs. without history of SCA/VA (p-values for difference = 0.0001), and with vs. without history of congestive heart failure (p-value for difference = 0.0334).

While we noted a few statistically significant results above, the extremely modest magnitudes of association call into question the clinical import of such findings.

**Results from the IPTW post-hoc analyses restricting to six months and one year of follow-up (Table S8)**

We found no association between empiric potassium supplementation at furosemide initiation and all-cause mortality, SCA/VA, or stroke when limiting “as-started” and “as-treated” analyses to six months and one year of follow-up.

**Results from instrumental variable (IV) analyses and assessment of IV strength and assumptions**

***Primary outcome: All-cause mortality***

Discordant with the as-started and as-treated findings from the Cox proportional hazards models for the <40 mg/day furosemide cohort, the prespecified IV analyses identified a risk increase of 16 deaths (95% CI 7–25) per 1,000 persons empirically supplemented with potassium (vs. not)—after adjustment for demographic, disease, drug use, laboratory, hospitalization, and healthcare utilization measure baseline covariates.

***Secondary outcomes: SCA/VA and Stroke***

Discordant with the as-started and as-treated findings from the Cox proportional hazards models for the ≥40 mg/day furosemide cohort, the prespecified IV analyses identified a risk reduction of 13 SCA/VA (95% CI -17–-8) per 1,000 persons empirically supplemented with potassium (vs. not)—after adjustment for demographic, disease, drug use, laboratory, hospitalization, and healthcare utilization measure baseline covariates.

Alternatively, concordant with the as-started and as-treated findings from the Cox proportional hazards models, adjusted IV analyses did not suggest a risk increase or reduction of stroke in either furosemide dose cohort.

***Assessment of IV strength and assumptions***

In the <40 mg/day furosemide cohort, we found 34% compliers and an F-statistic of 388.94, compared to 38% compliers and an F-statistic of 301.06 in the ≥40 mg/day cohort, which showed a relatively strong IV.(1) For the <40 mg/day furosemide group, we identified race, residence in long-term care/hospitalization on index date, CFI, congestive heart failure, CKD, pulmonary congestion, antiarrhythmics (type III), calcium channel blockers (dihydropyridines), and nitrates use in one year prior to index date, hospitalization in 30 days prior to index date, and a measure of healthcare intensity as imbalanced covariates across IV groups. When regressing those variables on the IV and other variables, we found statistically significant estimates for the IV, except for heart failure, which means that these measured variables (except heart failure) could be confounders that were associated with the IV. On the other hand, for the ≥40 mg/day furosemide group, we identified sex, race, Medicare Advantage enrollment, residence in long-term care/hospitalization on index date, congestive heart failure, CKD, cirrhosis, pulmonary congestion, renal transplant, aldosterone antagonist, antiarrhythmics (type III), calcium channel blockers (dihydropyridines), insulin, non-nitrate vasodilators and nitrates use in one year prior to index, hospitalization in 30 days prior to index, and several healthcare intensity measures as imbalanced covariates across IV groups. When regressing those variables on the IV and other variables, we found statistically significant estimates for the IV, thus these measured variables could be confounders that were associated with the IV. There were possibly unmeasured confounders that were associated with the IV as well; therefore, the assumption about the independence of the IV and unmeasured confounders could be violated. Despite the advantages of the IV analyses, due to our inability to test all the assumptions related to the IV, IV findings should be interpreted with extreme caution, especially when the findings seem to be sensitive to the analytical approaches. IV analyses might not be the most optimal method to answer this research question.
